# A 41-marker 37-color full spectrum flow cytometry panel for the deep immunophenotyping of human peripheral and liver natural killer cells

**DOI:** 10.3389/fimmu.2025.1609732

**Published:** 2025-12-10

**Authors:** Alberta Gerarda Antonia Paul, Konrad H. H. Reichel, Juan J. Garcia-Vallejo, Lara R. Heij, Maria C. Jaimes, Yacine Kharraz

**Affiliations:** 1 Scientific Commercialization Team (SCT), Cytek Biosciences Inc, Fremont, CA, United States; 2 Department of Molecular Cell Biology and Immunology (MCBI), Amsterdam Infection and Immunity and Cancer Center Amsterdam, Amsterdam University Medical Centers, Free University of Amsterdam, Amsterdam, Netherlands; 3 Department of General, Visceral and Transplant Surgery, University Hospital Essen, Essen, Germany; 4 Department of Pathology, University Hospital Rotterdam, Rotterdam, Netherlands; 5 Institute of Pathology, University Hospital Essen, Essen, Germany

**Keywords:** Aurora, high dimensional, flow cytometry, spectral, human NK cell, PBMCs, liver, cancer

## Abstract

Natural killer cells (NK cells) are granular lymphocytes with cytotoxic activity that have a role in both innate and adaptive immune responses. NK cells consist of a diverse array of phenotypes with specific functions imposed by the microenvironment. Liver NK cells are an abundant lymphocyte population playing a key role in tuning immune responses under physiological and pathological conditions. For example, NK cell functional and phenotypic changes occur during liver cancer progression and correlate with disease prognosis. As liver cancer has the second-highest mortality rate among solid cancers, it is important to define the composition and the dynamics of the liver and peripheral NK cell compartment both in health and disease state. In-depth analysis of the phenotypes and functional status of NK cells and their frequencies will expand our knowledge on their role in maintaining immune tolerance, disease progression, and aid the development of novel treatments. We present here a 41-marker 37-color spectral flow cytometry panel for the in-depth phenotyping of human peripheral and liver NK cells. This paper describes the first spectral flow cytometry panel with 35 markers potentially co-expressed on one cell type (NK cells) including the panel design process, sample preparation, staining protocol, quality control metrics, acquisition protocol and workflows to analyze NK cells in the periphery and liver. NK cell subsets and phenotypes were distinguished by including markers of differentiation, maturation, tissue residency, migratory potential, functional status, key transcription factors, and immune checkpoint molecules. Liver-type ILC1s (Lt-ILC1s) could be identified by inclusion of additional markers and modification of published gating strategies. Furthermore, we describe the dynamics of peripheral and liver NK cells. Finally, we show the validity of markers included to indicate NK cell dysfunction in samples of patients with Hepatocellular Carcinoma (HCC). This high parameter high resolution panel provides a key tool for in-depth delineation of distinct NK cell subsets in the periphery and in liver, in health and disease state. It allows for the robust identification of NK cells subsets with low frequencies and can effectively be used for samples with limited cell numbers.

## 1 Introduction

In-depth NK cell phenotyping is crucial to identify NK subsets and phenotypes that exert positive or detrimental effector functions under different pathological conditions. Furthermore, NK cell phenotyping would benefit the development of NK-targeting therapies and chimeric antigen receptor (CAR-NK) cell-based therapies that have become a major focus of the pharmaceutical industry and academic research (1, 2). Peripheral NK cells are classically divided into three main subsets based upon the relative expression of CD56 and CD16, namely CD56^bright^CD16^-^ (early NK cells), CD56^dim^CD16^+^ (mature NK cells) and CD56^-^CD16^+^ (terminal NK cells). Early NK cells are limited in cytotoxic function, produce proinflammatory cytokines, and express different cytokines, chemokine, adhesion and NK cell receptors than mature NK cells (3–6). Mature NK cells have high cytolytic capacity, produce proinflammatory cytokines, and mediate antibody-dependent cytotoxicity (ADCC) (3–7). Terminal NK cells proliferate less than mature NK cells, have limited cytokine responsiveness and accumulate with age and during chronic viral infections (8–10). Besides the classical NK cell subsets defined by CD16 and CD56 expression levels, other functional states have been identified with distinct expression of inhibitory (CD159a, CD159c, CD85j, KIRs), activating (CD337), chemokine (CD183, CX3CR1) and cytokine receptors (CD195), adhesion molecules (CD49e) and proteins that indicate differentiation (CD57, CD161) by using a variety of technologies (7, 11–17). Recently, a new NK cell classification was proposed dividing NK cells into 6 major subsets resembling subsets of mature (NK1A-C), early-stage CD56^dim^ (NKint), early (NK2) and adaptive CD159c^+^ (NK3) NK cells respectively (16). However, liver NK cells have not yet been investigated and classified with the same level of detail.

The liver receives 80% of its blood supply from the portal vein that drains the gastrointestinal tract. Consequently, it is constantly exposed to foreign antigens. Therefore, the liver immune compartment must maintain a status of local homeostasis and prevent activation and inflammation while in parallel it must aid the efficient clearance of pathogens. NK cells are an abundant population of the liver immune compartment as compared to the periphery (they represent almost 50% of liver lymphocytes) and play a key role in maintaining immune homeostasis in health and disease state (18–22). Liver NK cells are generally divided into two main phenotypes: CD56^dim^CD16^bright^ and CD56^bright^CD16^-^ that are present in relatively equal proportions. This is in contrast to the periphery where most NK cells are CD56^dim^CD16^bright^ (18). Based upon the site of residency and origin, three types of hepatic NK cells can be further distinguished: long-lived liver tissue resident NK cells (tr-NK) that are CD56^bright^CD16^-^ CD69^+^CD186^+^CD195^+/-^CD183^+/-^CD49a^+/-^Eomes^Hi^Tbet^low^, short-lived circulating conventional NK cells (cNK; CD56^dim^CD16^bright^), and adaptive/memory-like NK cells (ml-NK; CD56^bright^CD16^-^). Tr-NK cells are believed to have a key function in controlling viral infections, local tolerance and tissue homeostasis due to their expression of molecules that enforce their unique location close to liver sinusoidal endothelial cells (LSECs) lining the hepatic sinusoids that receive blood from the portal vein (23–26). Ml-NK cells are prone to respond upon re-exposure to viral antigens and include a population of CD159c^+^ NK cells that expand upon human cytomegalovirus infection (HCMV) (18, 26). The prevalence of chronic liver diseases (CLD) and liver cancer is increasing with Hepatocellular Carcinoma (HCC) being the most frequent liver tumor type (27, 28). Besides HCC, liver metastasis are also common in colorectal cancer (CRC) and a major cause of death. As a crucial role has been attributed to NK cells in disease progression and survival rates, both in HCC and CRC liver metastasis (20, 22, 29, 30), a clear understanding of the dynamics of peripheral and liver NK cell subsets, phenotypes and functional status is key for the development and monitoring of NK-based therapies. This reported diversity of NK cells also suggests that discrete subpopulations and/or distinct molecules need to be targeted and can vary per individual, disease state, and tissue type. Thus, it emphasizes the need for in-depth immunophenotyping of NK cells in the periphery and in tissues.

We report here on the development of a comprehensive 41-marker 37-color spectral flow cytometry panel that allows the identification of distinct NK cell subsets and phenotypes in the periphery and in the liver. This panel was based on two high dimensional NK panels previously described for conventional flow cytometry platforms (11, 31). Taking advantage of the resolution and multiplexing capacity of spectral cytometry, we expanded the published panels to include 35 markers potentially expressed on NK cells. Additionally, we established for each marker the sensitivity to enzyme digestion that was needed to isolate cells from liver tissues. We included CD94, CD49a, CD161, CD158b, CD158a-h, Eomes, Tbet and CD127 to distinguish NK cells from ILCs (32). Markers of tissue homing/residency and co-stimulation (CX3CR1, CD49e, CD69, CD186, CD49a, CD103, CD183, CD195, CD2, PLZF) were incorporated to identify tr-NK cells subsets/phenotypes (23, 31–37). Furthermore, the panel includes HLA-DR, CD38, Ki-67, Granzyme B and Perforin to evaluate functional responses to inflammation, activation and cytotoxic potential. By incorporating CD27, CD11b, CD159a, CD159c and CD57, it also allows detailed characterization of NK cell maturation and memory status (9, 38–42). Finally, antibodies directed to the immune checkpoint molecules TIGIT and CD226 (DNAM-1), inhibitory receptors CD85j and CD161, and activating receptor CD314 were added as they modulate NK cell functionality and are potential targets of cancer immunotherapies (43–49). As such, we present a panel that can serve as a key tool for NK cell functional studies in health and disease. In this method paper, we describe the process of sample preparation, panel design, panel optimization, panel verification, and provide a detailed description of the staining protocol and methods for data quality control. Additionally, we provide a workflow for sample analysis and NK cell subset/phenotype annotation.

## 2 Materials

### 2.1 Biological samples

Human PBMC were isolated from buffy coats (Sanquin Blood Bank, Amsterdam, The Netherlands) by means of density centrifugation with Lymphoprep (Axis-Shield, Oslo, Norway). Cells were resuspended to 10-20x10^6^/ml in 20% DMSO in Fetal Bovine Serum (FBS) and 1ml aliquots were stored at -80 ^°^C for future use. Liver biopsies from healthy livers were obtained from liver transplant donors, and liver biopsies from patients with HCC were sampled from the tumor site. Liver cell suspensions were generated from liver biopsies as described in (50). Briefly, biopsies were mechanically disrupted into small pieces using a scalpel. The resulting pieces were transferred into a 15 mL conical tube, with 9 mL of complete RPMI (10% FBS, 1% Pen/Strep and 1 mM Glutamine) and 1 mL of 10× hyaluronidase/collagenase solution (StemCell, 07912, Vancouver, BC, Canada). The first round of tissue dissociation by enzymatic digestion was done at 37 °C for 30 min in a pre-warmed shaker. The supernatant was collected without disrupting the tissue and a fresh digestion media was added (10 ml complete RPMI containing 128 U/ml of collagenase IV (Lorne Laboratories, LS004194, Danhill, Berkshire, UK), 40 U/ml of DNaseI (Sigma, DN25, Gillingham, Dorset, UK) and 25 U/ml of universal nuclease (Pierce, 88702, Waltham, MA, USA) for an additional 30 min of digestion. The supernatant was combined with the one from the first digestion step and the remaining liver pieces were squeezed through a 70 µm tissue strainer and rinsed with 10 mL of complete RPMI. The supernatants from all digestion steps were combined and centrifuged for 10 min at 300 g. Red blood cells (RBCs) were removed with ACK lysing buffer (Gibco™, A10492-01, Paisley, UK). Isolated cells were resuspended in 20% DMSO in FBS and aliquots (2.2-5.6x10^6^ cells/vial) were stored at -80 ^°^C for future use. As the high parameter flow cytometry panel was designed to determine NK cell phenotypes both in the periphery and in liver cell suspensions, obtained after digesting liver biopsies, it was key to verify that the expression of the chosen markers was not affected by the enzyme digestion method used. To determine which markers were affected, freshly isolated PBMC were treated with the same protocol as used for obtaining liver cell suspensions, frozen and stored at -80 ^°^C until use.

### 2.2 Ancillaries and reagents for flow cytometric staining

- Falcon® FACS tubes 12x75 mm, 5 ml (Corning, catalogue #352063) or equivalent.
- 10, 200 and 1000 µl pipet tips (ThermoFisher Scientific, catalogue #9400310, 94300220, 9401030) or equivalent.
- 10, 20, 200 and 1000 µl pipettors (ThermoFisher Scientific, catalogue #4642030, 4642050, 4642080, 4642090) or equivalent.
- 1.5 ml eppendorf tube (ThermoFisher Scientific, catalogue #3451PK) or equivalent.
- RPMI 1640 (Sigma-Aldrich®, catalogue # R8758).
- FBS (Corning GmbH, catalogue #35-079-CV).
- PBS (Gibco™, catalogue #20012-027).
- BD Horizon™ Brilliant Stain Buffer Plus (BD Biosciences, catalogue #566385).
- True-Stain Monocyte Blocker™ (BioLegend, catalogue #426101).
- UltraComp eBeads™ Compensation Beads (ThermoFisher Scientific, catalogue #01-2222-41).
- eBioscience™ Foxp3/Transcription Factor Staining Buffer Set (ThermoFisher Scientific, catalogue #00-5523-00).
- 4% Paraformaldehyde (Santa Cruz Biotechnology, catalogue#30525-89-4) or equivalent.
- Thawing medium (RPMI 1640 supplemented with 10% FBS).
- Staining and washing buffer (PBS with 0.05% Bovine Serum Albumin).
- Vendor and catalogue numbers of antibody reagents used in the panel are listed in **Supplementary Table 1**.

### 2.3 Equipment

This panel was developed for a Cytek® Aurora (Cytek Biosciences Inc., Fremont, California) equipped with 5 lasers (355, 405, 488, 561, 640 nm) and 64 detectors. The complete configuration with laser power, number of detectors per laser module, center wavelength, bandwidth of the filters, is detailed in (51, 52).

## 3 Methods

**Figure 1:**
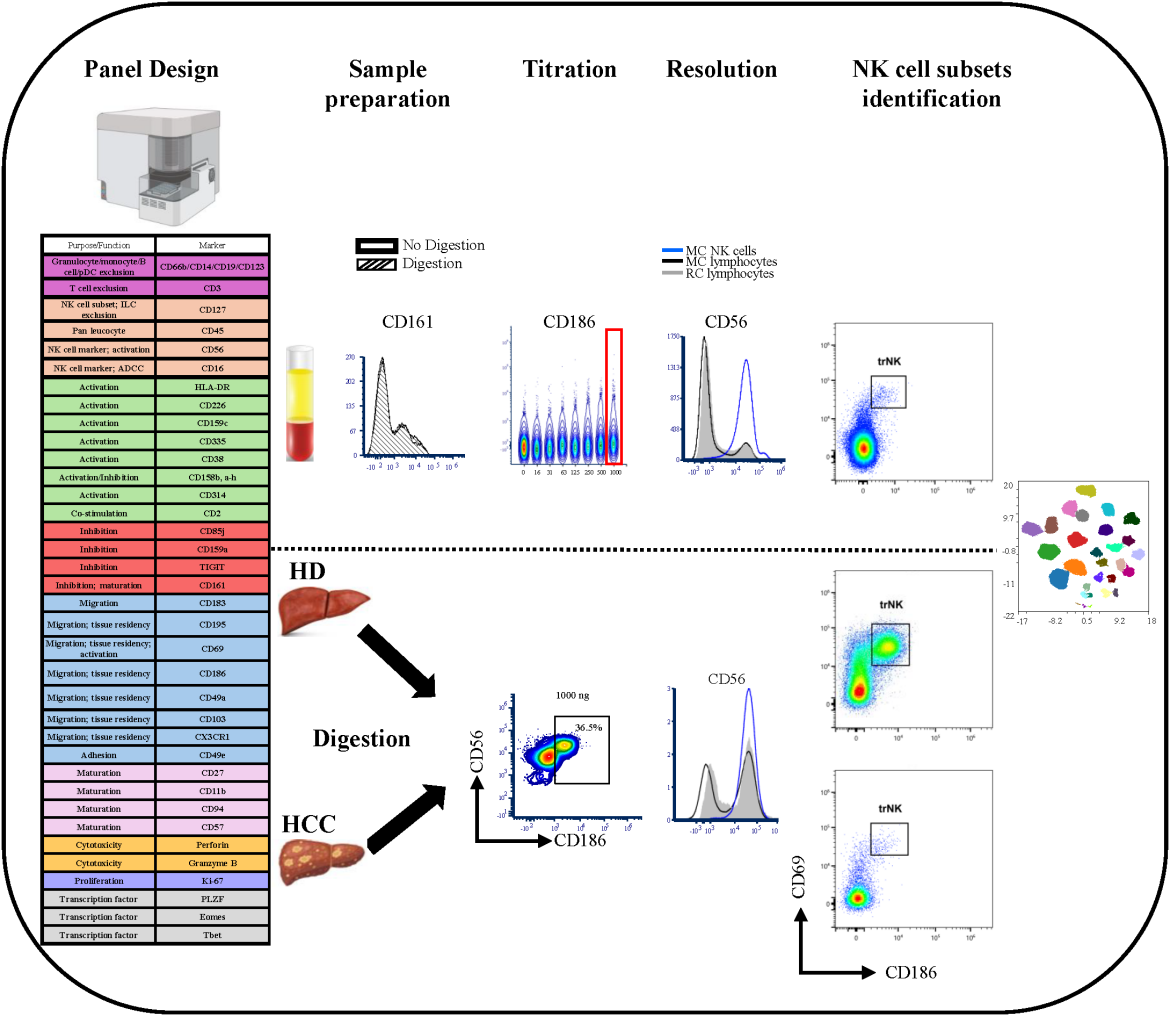
Summary of the major steps in the panel development and verification process. The major steps in panel development were as follows: First a panel design was generated using estabilshed metrics. Secondly preservation of the staining patterns/percentages of all markers was confirmed after sample preparation (digestion). Further optimization involved titration of all reagents, confirming preservation of resolution in the MC sample and establishing gating strategies for PBMCs and liver samples. Finally, manual gating and multidimensional analysis was performed for NK cell subset identification.

A summary of the major steps in the panel verification process that are addressed in this chapter is depicted in **Figure 1**.

### 3.1 Clone selection and panel design

The panel design (markers, clones, and fluorochromes) was based on two high dimensional panels for NK cells that were developed for conventional flow cytometry platforms (11, 31). We expanded those panels to accommodate additional markers while preserving the overall resolution. We followed the rules for panel redesign as described in (51, 52). First, we classified markers that were added as primary, secondary, and tertiary (53). Secondly, we assessed the level of co-expression on peripheral and liver NK cells based upon the literature. Thirdly, we used the Cytek® Cloud to select unique fluorochrome spectra to accommodate all the markers of the panel thereby avoiding fluorochromes with a high cosine similarity index and avoiding a high condition number as described in (51, 52). Finally, we limited the use of custom reagents. Based on the criteria stated above (51) we selected 37 fluorochromes (**Supplementary Figures 1A**, **C**). Of the 37 fluorochromes selected, and as depicted in **Supplementary Figure 1B**, the pairs with the highest Similarity™ indices were Vio® Bright B515 and Vio® Bright FITC (0.93), BV421 and Super Bright™ 436 (0.96) and PE and cFluor® YG584 (0.91). However, the condition number of the panel was low (16.82; **Supplementary Figure 1B****;** black arrow) and hence this metric gave us confidence that this fluorochrome combination was appropriate. We next generated the Spillover Spread Matrix (SSM; **Supplementary Figure 2C**) from PBMCs stained with anti-CD4 antibodies available for the selected fluorochromes, except for RealBlue™744 (RB744) and RealBlue™780 (RB780) which were CD3 and CD2 conjugates respectively. The SSM was used to predict areas of spread and to guide further marker-fluorochrome assignment when needed.

Despite the high Similarity™ indices between the combinations of fluorochromes stated above, these fluorochromes pairs could still be sufficiently discriminated from each other both in PBMCs (**Supplementary Figure 2A**) and in the liver (**Supplementary Figure 2B**) when stained with the final panel. Markers that were added to this panel as compared to (11, 31) were the exclusion markers CD123 (for pDCs) and CD66b (for granulocytes). Although this panel was run on frozen/thawed samples, the addition of CD66b allows to exclude granulocytes in case fresh samples are analyzed. Additionally, we added CD226, CD183 and CD94 as they indicate the functional status and/or distinguish specific NK cell phenotypes and can be used in the gating strategy to exclude ILCs (CD94^-^). Some markers needed to be reassigned to different fluorochromes and/or clones due to performance issues after initial testing. For example, CD161 was first assigned to BV605 and clone DX12 was chosen. As the ability of this reagent to discriminate CD161^+^ from CD161^-^ was suboptimal, we switched to clone HP-3G10 conjugated to cFluor® R720. Another example was CD195, originally assigned to BUV395, that was reassigned to the brighter fluorochrome BUV661 because of the dim expression of CD195 on NK cells. Final markers, clones and fluorochromes that were used in this panel are listed in **Table 1**.

**Table 1:** Markers included in the panel design (in alpha numeric order) and their specifics.

Specificity	Alternative name	Fluorochrome	Clone	Purpose/function
CD103	Integrin alpha E	BUV805	Ber-ACT8	Migration, tissue residency
CD11b	Integrin alpha M, ITGB2 subunit A	BV570	ICRF44	Maturation, NK cell subsets
CD123	IL-3 receptor alpha, IL-3RA	cFluor V450	6H6	Exclusion plasmacytoid dendritic cells
CD127	IL-7Ra	BV421	A019D5	Exclusion ILC, NK cell subsets
CD14	N.A.	cFluor V450	M5E2	Exclusion monocytes
CD158a-h	KIR2DL1, KIR2DS1	PE-Cy5.5	EB6 (11PB6)	Activating/inhibitory receptor
CD158b	KIR2DL2, KIR2DL3, KIR2DS2	PE-Cy5.5	GL183	Activating/inhibitory receptor
CD159a	NKG2A	PE-Vio 615	REA110	Inhibitory receptor
CD159c	NKG2C	BV480	134591	Activating receptor, NK subsets
CD16	FcgRIII	BUV496	3G8	NK subsets, Antibody Dependent Cellular Cytotoxicity (ADCC)
CD161	NKR-P1	cFluor R720	HP-3G10	Inhibitory receptor, maturation marker; NK cell subsets
CD183	CXCR3	PE-Cy5	1C6/CXCR3	Migration
CD186	CXCR6	BV711	13B1E5	Migration, tissue residency
CD19	N.A.	cFluor V450	HIB19	Exclusion B cells
CD195	CCR5	BUV661	2D7/CCR5	Migration, tissue residency
CD2	Cluster of differentiation 2	Super Bright 436	RPA-2.10	Co-stimulatory receptor
CD226	DNAM-1, DNAX accessory molecule	BUV615	DX11	Activating receptor
CD27	Tumor necrosis factor receptor superfamily 7	cFluor YG584	O323	Maturation, NK cell subsets
CD3	N.A.	RB744	UCHT1	Exclusion T cells
CD314	NKG2D, KLRK1	cFluor BYG750	1D11	Activating receptor
CD335	NKp46, NCR1	Vio Bright B515	REA808	Activating receptor
CD38	Cyclic ADP ribose hydrolase	APC-Fire 810	HIT2	Activation marker
CD45	Protein tyrosine phosphatase receptor C	PerCP	HI30	Pan leucocyte marker
CD49a	Integrin alpha 1, VLA-1, ITGA1, ITA1	BV750	SR84	Migration, tissue residency
CD49e	integrin α5	Vio Bright FITC	REA686	Cell adhesion
CD56	Neural cell adhesion molecule (NCAM)	BUV563	NCAM16.2	NK subsets, activation
CD57	HNK-2, Leu-7	APC-Vio 770	REA769	Maturation marker
CD66b	CEACAM8, CGM6, NCA-95	Pacific Blue	G10F5	Exclusion granulocytes
CD69	Cluster of differentiation 69	BUV737	FN50	Migration, tissue residency, activation
CD85j	ILT2, LIR-1	RB780	GHI/75	Inhibitory receptor
CD94	killer cell lectin-like receptor subfamily D member 1, KP43	PE-Cy7	DX22	Exclusion ILC, NK subsets
CX3CR1	Fractalkine receptor	BV650	2A9-1	Migration, tissue residency
Eomes	Eomesodermin, T-box brain protein 2 (Tbr2)	eFluor 660	WD1928	Transcription factor
Granzyme B	N.A.	BV510	GB11	Cytotoxic potential
HLA-DR	MHC Class II	BUV395	G46-6 (L243)	NK cell activation marker
Ki-67	N.A.	BV605	B56	Proliferation marker
Perforin	N.A.	PerCP-eFluor 710	dG9	Cytotoxic potential
PLZF	Zinc finger and BTB domain Domain containing 16	PE	R17-809	Transcription factor
Tbet	T-box transcription factor, Tbx21	BV785	4B10	Transcription factor
TIGIT	T-cell immunoreceptor with Ig and ITIM domains, WUCAM, VSTM3	APC	MBSA43	Checkpoint inhibitor molecule
Viability	N.A.	Live Dead UV Blue	NA	Viability

Antigens included in the panel annotated according to the CD classification (where applicable) with their alternative name, assigned fluorochrome, clone and the purpose/function in the panel.

### 3.2 Antibody titrations

All selected reagents were titrated using frozen PBMC from healthy donors (HD). An average of 1.10^6^ cells were used per test and subjected to fixation/permeabilization method as described in the staining protocol. 5 µl of True-Stain Monocyte Blocker was added before adding dilutions of antibodies. Antibodies, whether bottled at µg/test or ul/test, were tested starting at two-fold the manufacturer recommended titer, followed by seven serial dilutions (except PLZF; 6 serial dilutions). All titrations were done in a total volume of 250 µl for 25 minutes at RT. Optimal titers were selected based on highest staining index, saturation of percentage positives and signal intensities (Median Fluorescence Intensity, MFI) as described (54). Files were unmixed with autofluorescence (AF) extraction and concatenated for analysis using FCS Express™ version 7. Concatenated plots of titration results as well as the stain indices and the frequencies of the positive populations are depicted in **Supplementary Figures 3A, B** respectively. The selected titers for all reagents are listed in **Supplementary Table 1**. Two markers that were detected at very low frequency in PBMCs (CD49a and CD186) were additionally titrated on liver samples from a HD, confirming that the selected titer for PBMCs also applied to liver samples (data not shown). In addition, we carefully evaluated non-specific binding in parallel to the selection of the optimal titers based upon the criteria mentioned above. As expected, the use of True-Stain Monocyte Blocker™ significantly eliminated non-specific staining on monocytes for cyanine-based fluorochromes. Therefore, all titrations and multicolor staining were done with the inclusion of True-Stain Monocyte Blocker™. Of note, the inclusion of Fc-block had no effect on monocytes background staining and did not improve further the effect of adding True-Stain Monocyte Blocker™ (data not shown).

### 3.3 Effect of the digestion protocol on reagent performance

As liver samples preparation required digestion steps, it was important to verify that the expression of the markers in the panel was not affected by this treatment. We therefore assessed the impact of the dissociation protocol on the antigen integrity on PBMCs by comparing the percentage of positive populations and staining intensities to untreated PBMCs of the same donor after staining with the optimal titers. Although liver samples might be less sensitive to the digestion procedure, either because some cell epitopes can be hidden due to the association of cells to the extracellular matrix or because of the presence of tissue polyphenols that can partially inhibit digestive enzymes, PBMCs that undergo enzymatic digestion does provide important information on whether the antigens detected in the panel contain a peptide substrate for the digestion enzymes used in the protocol. **Supplementary Figure 4A** shows markers that were not affected by enzyme treatment. Pseudocolor plots were used for CD159c, CD69 and CD49a, as the frequency of these populations was low in the tested samples. CD159c showed no changes in MFI and frequency after digestion. On the other hand, CD69 and CD49a showed an increase in percentage positive events but similar staining intensities after digestion (**Supplementary Figure 4B**). We observed a decrease in the MFI of CD56 after digestion, as previously described (11). However, the percentage of CD56^+^ events remained the same (**Supplementary Figure 4C**) which confirmed that this marker can be used with confidence for NK cell phenotyping in digested tissues. **Supplementary Figures 4D, E** illustrate the loss of signal of NKp80 and CD337 (NKp30) after the digestion protocol, regardless of the antibody clone used to stain the cells. While we originally intended to target these antigens in our panel, we decided to exclude them based on these findings. Of note, loss of NKp80 and CD337 staining upon treatment of PBMCs with different commercially available digestion kits has been documented by the manufacturer (Miltenyi Biotech;

https://static.miltenyibiotec.com/asset/150655405641/document_a8tksr6uf95id9ohsujoh08q0o?content-disposition=inline,https://static.miltenyibiotec.com/asset/150655405641/document_p5pedude156p1dta8vo2aelc7s?content-disposition=inline).

### 3.4 Sequential staining

Sequential staining has been shown to be beneficial when working with high dimensional flow cytometry panels either due to effects of different staining volumes or steric hindrance of antibody-fluorochrome conjugates combinations (51, 52). Optimization of the staining protocol was therefore performed, and we concluded that the staining for CD161 and CD314 needed to be done as a first staining step (cocktail A). Similarly, addition of NK cell receptors antibodies in a separate staining step (cocktail B) improved the resolution for CD159a, CD335 and CD85j when compared with the staining including those antibodies in the master mix (cocktail C) (**Supplementary Figure 5A**). MFI and frequencies of positive populations are provided in **Supplementary Figure 5B**. Although NKG2c was not evaluated the decision was made to add this antibody at the same step as other NK cell receptors (cocktail B). Based on previous reports that the resolution of the chemokine receptors CD195, CX3CR1 and CD183 is negatively impacted when stained alongside other antigens (51, 52, 55), we decided to also add these antibodies sequentially. In order to respect the order of addition of these antibodies as described, CD183 was added to cocktail A while CX3CR1 and CD195 were added sequentially after cocktail B. As sequential staining leads to longer incubation times than the 25 min set for titrations, we also decided to rerun antibody titrations with incubation times matching those of the multicolor tube staining: 35 min for CD195, 45 min for CX3CR1, 55 min for antibodies of cocktail B and 65 min for antibodies of cocktail A. Although the stain indices were overall improved with longer times of incubation, no difference was observed in positive population frequencies or optimal titers (data not shown). The final order of addition is presented in the protocol and outlined in **Supplementary Figure 6E** and **Supplementary Table 1**.

### 3.5 Thawing PBMCs and liver samples

After thawing, cell recovery and viability must be assessed to ensure no artifacts are introduced in the data. The cell recoveries and viability of the samples used in this study, and the final number of NK cells acquired is provided in **Supplementary Table 2**.

Note: Handling of human biological components should be done in accordance with regional and institutional biosafety policies and/or requirements.

1. Pre-warm thawing medium at 37 °C for at least 30 minutes.
2. Thaw cells as quickly as possible.
3. Thaw cryo-vial in a 37 °C water bath, until only a small piece of ice remains.
4. Transfer the contents of cryo-vial to 50 ml conical tube.
5. Add 1 ml of warm thawing medium to the empty cryo-vial. Leave aside until step 8.
6. Drop-by-drop add 5 ml of thawing medium to the cells in the 50 ml tube. While adding, gently mix the 50 ml tube (with a pipette in one hand and in the other the 50 ml tube, add the thawing medium while you gently swirl the tube).
7. After the first 5 ml of thawing medium has been added, add the next 5 ml a little bit faster (a few drops at a time).
8. After 10 ml have been added, pour the 1 ml content of the cryo-vial into the 50 ml tube.
9. Add an additional volume of thawing medium to bring the total volume to 20 ml.
10. Spin at 400 g for 5 minutes.
11. Decant supernatant carefully without disturbing the pellet.
12. Gently resuspend the pellet in 2 ml of thawing medium. Take 5 µl for counting.
13. Complete to 20 ml.
14. Repeat steps 10 and 11.
15. Resuspend in staining buffer supplemented with 5 µl of True-Stain Monocyte Blocker/100µl of cell suspension to reach an approximate concentration of 10x10^6^/ml for PBMCs. Liver samples (0.4-1.5x10^6^ cells) were resuspended in 500 µl staining buffer supplemented with 5 µl True-Stain Monocyte Blocker™/100 µl cell suspension and filtered through a 70 µm tissue strainer before staining.
16. Once the staining has begun, samples need to be processed without delay.

### 3.6 Viability dye, antibody dilutions and multicolor (MC) antibody cocktails preparation

1. Thaw an aliquot of Live/Dead Fixable Blue viability dye (aliquoted according to manufacturer recommendations).
2. Prepare a 1:40 dilution in PBS by adding 5 µl of the viability dye to 195 µl PBS.
3. Keep in the dark until usage.
4. Prepare antibody dilutions as needed in staining buffer.
5. Prepare MC antibody cocktails A, B and C (surface) and D (intracellular) according to **Supplementary Table 1** by adding 10 µl Brilliant Stain Buffer Plus into an Eppendorf tube and adding each antibody at the determined titer. Mix gently after adding each antibody.
6. Add 10 µl of prediluted viability dye to the pellet, vortex gently and incubate for 15 minutes in the dark at RT.

### 3.7 Staining protocol for MC samples and reference controls 

A summary of the staining protocol is depicted in **Supplementary Figure 6**.

Samples were aliquoted over the different tubes before staining. For MC tubes 300 µl PBMCs ( ± 3x10^6^ PBMCs) and 400 µl liver cells ( ± 0.32 – 1.36 x10^6^ liver cells) were aliquoted and 100 ul for each RC ( ± 1x10^6^ cells PBMC). Additionally, 100 ul of each sample was aliquoted to be used as a sample specific unstained. For the RC on beads, the manufacturer recommended volume of beads was aliquoted per tube (1 drop per tube after vigorous vortexing of the stock vial) and washed once in staining buffer before addition of antibodies. Both the MC tubes and RC for viability staining should be washed with 3 ml PBS to remove any protein before viability staining.

1. Add 3 ml PBS and centrifuge at 400 g for 5 minutes at RT.
2. Decant supernatant and vortex gently.
3. Repeat step 1 and 2
4. Add 10 µl of prediluted viability dye to the pellet, vortex gently and incubate for 15 minutes in the dark at RT.
5. Wash the MC tubes and all the RC with 3 ml staining buffer
6. Centrifuge at 400 g for 5 minutes at RT
7. Decant supernatant and vortex gently.
8. In the meantime, stain the RC (cells or beads) for surface markers with the appropriate titers of antibodies: for the RC on cells, add 5 µl of True-Stain Monocyte Blocker to all RC tubes before adding the antibodies.
9. Add 300 µl of staining buffer to the viability RC and store in the dark.
10. Add 10 µl of Brilliant Stain Buffer Plus and 5 µl of True-Stain Monocyte Blocker™ to all the MC tubes and vortex gently.
11. Add MC antibody cocktail A and incubate for 10 minutes in the dark at RT; vortex gently.
12. Add the MC antibody cocktail B (see **Supplementary Table 1**, calculate how much volume is needed per sample based on the volumes of each of the individual antibodies), vortex gently and incubate for 10 minutes in the dark at RT.
13. Add anti-CX3CR1 BV650 and incubate for 10 minutes in the dark at RT; vortex gently.
14. Add anti-CD195 BUV661 and incubate for 5 minutes in the dark at RT; vortex gently.
15. Add the MC antibody cocktail C (see **Supplementary Table 1**, calculate how much volume is needed per sample based on the volumes of each of the antibodies) and incubate for 25 minutes in the dark; vortex gently.
16. Add 3 ml staining buffer.
17. Centrifuge at 400 g for 5 minutes at RT
18. Decant supernatant and vortex gently
19. Repeat steps 16, 17 and 18.
20. Add 250 µl of 4x prediluted fixative solution (eBioscience™ Foxp3/Transcription Factor Staining Buffer Set), prewarmed to RT, to all the MC tubes and RC and vortex gently. Incubate for 40 minutes at RT; repeat vortexing after 20 min. NOTE: if beads are used for intracellular markers, they should not be treated with fixative but only stained after washing with permeabilization buffer to ensure that the intracellular antibody reagent is exposed to the same condition/buffers as in the MC sample.
21. Add 2 ml of 10x prediluted permeabilization buffer (eBioscience™ Foxp3/Transcription Factor Staining Buffer Set), prewarmed to RT, to the MC tubes and all the RC.
22. Centrifuge at 800 g for 5 minutes (it is important to increase the centrifugation speed at this step and thereafter as cellular weight decreases after fixation and particularly permeabilization).
23. Decant supernatant and vortex gently.
24. Repeat step 21 and leave the tubes in the dark at RT for 5–10 minutes before proceeding with step 25 (waiting before centrifugation ensures that cells are permeabilized before adding the antibodies for intracellular staining). The RC on beads can be washed once.
25. Repeat step 22–23.
26. Add the MC antibody cocktail D (see **Supplementary Table 1**, calculate how much volume is needed per sample based on the volumes of each of the intracellular antibodies), vortex gently, and incubate for 30 minutes in the dark at RT. At the same time add the intracellular antibodies to the RC on cells or beads at the indicated titers.
27. Add 2 ml of 10x prediluted permeabilization buffer and leave the tubes in the dark at RT for 5-10 minutes before proceeding with step 28 (waiting before centrifugation helps reduce unspecific staining).
28. Centrifuge at 800 g for 5 minutes.
29. Decant supernatant and vortex gently.
30. steps 27, 28 and 29. *If samples are immediately acquired, follow the steps below*:
31. Wash with 2 ml of staining buffer.
32. Centrifuge at 800 g for 5 minutes.
33. Decant supernatant and vortex gently.
34. Add 150 µl of staining buffer and acquire using CytekAssaySetting (CAS) at medium flowrate. *If samples are acquired after 4 h follow the steps below:*
35. Add 200 µl of 1% paraformaldehyde (4% diluted to 1% with PBS) and incubate for 15 minutes in the dark at RT; vortex gently.
36. Wash with 2 ml of staining buffer.
37. Centrifuge at 800 g for 5 minutes.
38. Decant supernatant and vortex gently
39. Add 150 µl of staining buffer and acquire or store at 4 degrees in the dark until analysis within 24h (longer times until acquisition were not tested).

### 3.8 Instrument set-up and QC

Daily Instrument Quality Control (Daily QC) was run before each new experiment acquisition using SpectroFlo® QC beads, lot 2005. Settings provided by the manufacturer (referred to as CytekAssaySetting in SpectroFlo^®^ software) were used as a starting point for instrument setup with adjustments in FSC-A and SSC-A gains and FSC threshold settings for optimal visualization of lymphocytes vs. monocyte populations and reducing the collection of debris.

### 3.9 Determination of optimal controls for unmixing

Optimal single stained controls (named Reference Controls in SpectroFlo software, RC) are essential to ensure accurate unmixing. As a first step of RC optimization, determining if beads lead to accurate unmixing is essential. Indeed, the spectrum of antibody-conjugated fluorochromes can differ when binding to cells or beads. As these emission spectrum mismatches can lead to unmixing errors in an assay specific manner, the use of beads must be assessed empirically (51). To do so, we stained both beads and cells in parallel, applying the same protocol, reagents and titers as for the MC sample. Optimal RC were selected by the method described in (51). Description of optimal RC (cells or beads) and cell numbers to be acquired to ensure collection of enough positive events are listed in **Supplementary Table 3**. This panel also includes a combination of two markers in PE-Cy5.5, namely CD158b and CD158a-h. As PE-Cy5.5 is a tandem-dye, its spectrum can vary from lot to lot due to differences in energy transfer between donor and acceptor molecules. We therefore assessed whether the two spectra were different. We observed a perfect overlap between both signatures (data not shown) showing that both markers in PE-Cy5.5 have identical spectra and can therefore be used as RC indifferently.

### 3.10 Autofluorescence extraction and exclusion of RBCs

Cellular suspensions from digested solid tissues can be highly and heterogeneously autofluorescent. Several publications reported that including multiple autofluorescence (AF) spectra as reference controls to perform the unmixing might be indispensable to ensure that data is exempt of any artifact and leads to correct result interpretation (56–60). The liver being a highly AF tissue, we then wondered whether our samples needed the application of such approach. In the case of this study, although liver samples displayed a high and heterogenous AF (**Supplementary Figure 7A**), we found that applying multiple AF extraction was not required for accurate unmixing and resolution of the NK markers as lymphoid cells showed homogenous AF as opposed to cell types such as myeloid or mesenchymal cells. Despite a great variability in the morphology of the cells extracted from the liver, lymphocytes were easily identified by SSC and FSC. We found that gating tightly on the corresponding population was enough to clean up most of the irrelevant AF spectral signatures and obtain a well-defined AF spectrum (**Supplementary Figure 7B**). We therefore applied a default unmixing with AF extraction with the gate set on the lymphocytes. However, despite the successful cleaning of irrelevant AF using the SSC and FSC parameters, one a small population still appeared to be unproperly unmixed (red arrows in top and middle lanes, **Supplementary Figure 7C**) (61). This was evidenced by its super negative median fluorescence intensities (MFI) in several fluorescence parameters, suggesting that a specific cell lineage lying in the lymphocyte gate had a different AF. Because of their small FSC-SSC overlapping partially with lymphocytes, we hypothesized these could be RBCs. One of the advantages of the Aurora spectral analyzer used in this study is the possibility to measure light scattering using both the violet and the blue laser simultaneously. Because RBCs lack a nucleus, their absorbance of the light at these wavelengths is different from nucleated cells allowing for their easy discrimination as already reported in a previous publication (61). After further investigation, this population was identified as RBCs based on their scattering of the blue and violet laser light. We could therefore easily eliminate them with an appropriate gating strategy (**Supplementary Figure 7C** bottom lane). Once an accurate gating strategy was applied to the data, we found that a default AF extraction is highly beneficial, especially for those fluorochromes emitting in high AF area (**Supplementary Figure 7D**).

### 3.11 Evaluation of the unmixing accuracy of MC samples

MC samples were unmixed with the SpectroFlo® software V3.2.1. To check the unmixing accuracy of the MC tube steps were followed as in (51). Briefly, the data were cleaned up (cleaning gates included time, singlets, live, and aggregate exclusion when needed), and NxN permutations were displayed. The multicolor samples were screened for unmixing errors by visually inspecting the NxN plots of one fluorochrome versus all the other fluorochromes in the panel. Spillover corrections were only applied when the observed unmixing errors were between fluorochromes with known spillover and were guided based on well-characterized and described staining patterns. Unmixing accuracy was very high with 3–16 corrections below 5% in the 1722 combinations (including the AF parameter) except for BUV661 into BV605 for which corrections between 3.2 and 7.2 % were needed. The relatively high correction for BUV661 into BV605 was confirmed by Fluorescence Minus One controls (data not shown).

### 3.12 Panel resolution assessment

To ensure that the theoretical panel design resulted in limited areas of spread and, if spread occurred, this was in regions in which markers are not coexpressed or at dim levels, we first assessed spread by calculating the SSM of PBMC stained with the final panel reagents at established titers. Of the 1,369 possible combinations of fluorochromes, only 17 marker fluorochrome combinations had a spillover and spread value (SSV) above 6 (**Supplementary Figure 8A**). The limited number of combinations exhibiting relatively high spread confirmed the robustness of the fluorochrome selection and panel design. **Supplementary Figure 8B, C** show a few marker combinations with SSV above 6 for PBMCs **(B)** and liver from a HD **(C)**. The highest SSV was for the Granzyme BV510 and CD16 BUV496 combination, but its impact on data resolution was negligible.

Resolution loss can also occur because of interactions between reagents, such as steric hindrance. We therefore systematically compared the resolution of each marker in the MC tube with its corresponding RC, both on PBMCs and on liver. For the resolution comparison we overlayed the histograms of gated singlets/lymphocytes or singlets/monocytes (when applicable) of single stained samples (grey filled) with the MC sample (bold black line). Additionally, we overlayed the gated total NK cells (bold blue line; defined by sequentially gating on CD45^+^CD3^-^Lin- and subsequently gating out ILC’s (CD94^-^CD127^+^), CD56^-^HLA-DR^+^ and CD56^-^CD16^-^ events) for NK cell markers. Note that the single stained sample and the MC sample were obtained from the same donor and stained with the exact same protocol (incubation time, volume, fixation and permeabilization methods) in order to make an accurate comparison. **Supplementary Figures 9A, B** show a perfect match for all markers both in PBMCs and HCC liver. Importantly, the NK cell gated population demonstrated excellent resolution of all NK cell markers, therefore confirming appropriate panel design.

### 3.13 Multidimensional analysis

**Figure 2:**
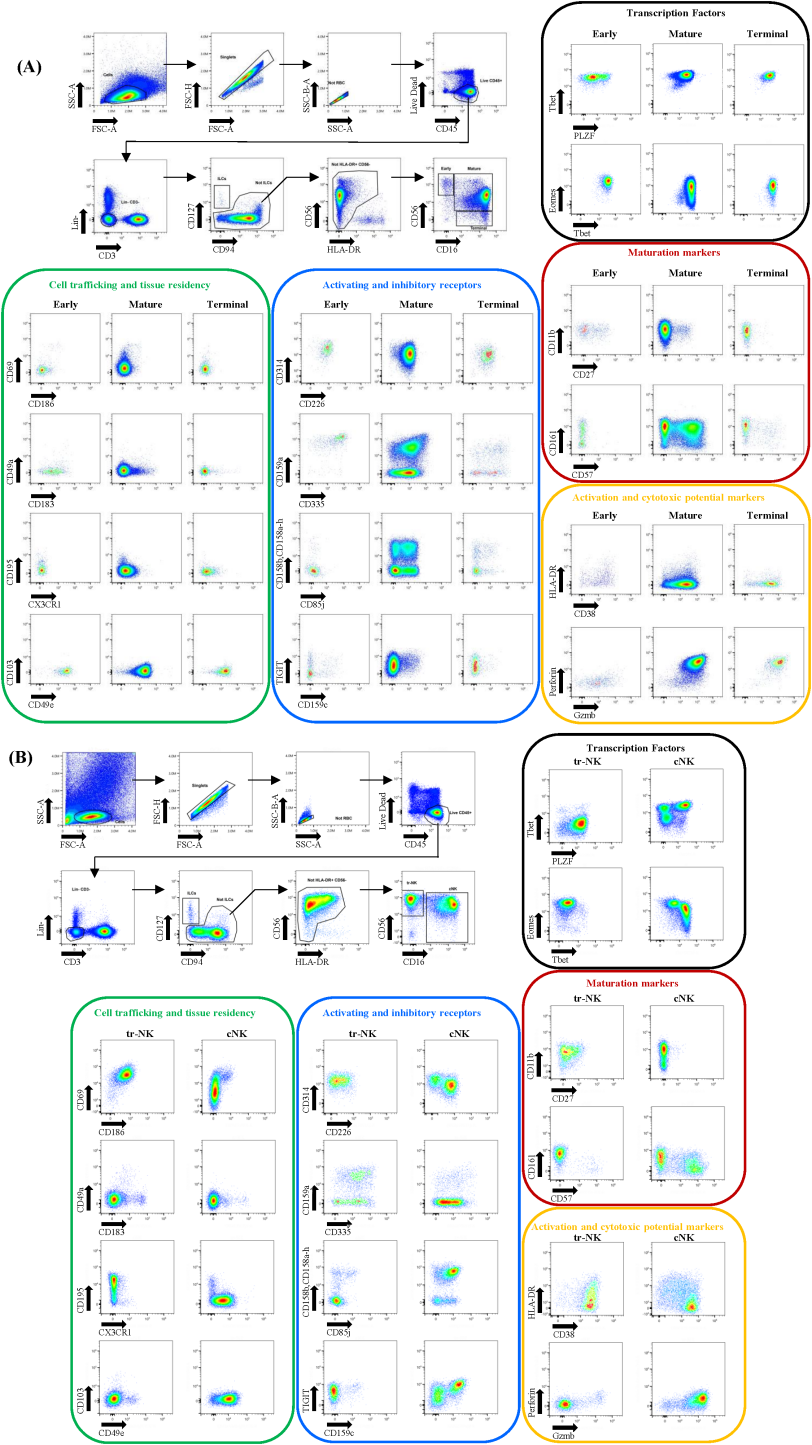
Gating strategy for NK cell identification in PBMCs and liver samples. **(A)** Gating strategy for NK cell identification in PBMCs. Three major NK cell subsets were gated: Early, Mature and Terminal NK cells. **(B)** Gating strategy for NK cell identification in HD liver. Two major NK cell subsets were gated: tr-NK and cNK cells. The expression levels of markers indicating cell trafficking/tissue residency, activation and inhibitory receptors, maturation, transcription factors and activation and cytotoxic potential are shown for each gated major subset.

After gating on lymphocytes using FSC-A/SSC-A, excluding the doublets by gating on FSC-A/FSC-H, excluding the RBCs by gating on SSC-A/SSC-B-A, gating on viable leukocytes (CD45^+^ Live Dead Blue^-^), getting rid of B-cells/monocytes/pDC/granulocytes (Lin^-^) and T-cells (CD3^-^), gated CD94^+/-^CD127^+/-^ events were exported (from SpectroFlo^®^; see gating strategy **Figures 2A, B**) into the OMIQ platform (https://www.omiq.ai/). Files were further analyzed using an OMIQ pipeline according to (52) with adjustments of using FlowCut (62) and Phenograph (63). After scaling optimization (Arcsinh), the FlowCut algorithm was applied to remove outlier events due to abnormal flow behaviors. All files were then further manually gated to exclude CD127^+^CD94^-^ and HLA-DR^+^CD56^-^ events (as in **Figures 2A, B**) and finally gated on total NK cells (CD56^+/-^CD16^+^ encompassing early, mature and terminal NK cells). Files were then concatenated before running the Phenograph clustering algorithm, Unified Manifold Approximation and Projection (UMAP) algorithm and heatmap generation. Clustering analysis of total NK cells with the PhenoGraph algorithm, included all markers (except Live Dead Blue, CD45, Lin, and CD3) with using 40 K Nearest Neighbors and Euclidean distance metrics. After PhenoGraph analysis, dimensionality was reduced by means of UMAP with the following parameters and settings: all markers as parameters (except Live Dead Blue, CD45, Lin and CD3) and including the PhenoGraph parameter, Neighbors 80, Minimum Distance 0.7, components 2, Euclidean metric, Learning rate 1, Epochs 250, Random Seed 5320 and spectral embedding initialization. PhenoGraph clusters were overlayed on the UMAP for visualization of the distribution of the clusters between sample types and individual samples. Following the UMAP analysis, a heatmap was generated by combining all files and hierarchically ordering the PhenoGraph clusters.

## 4 Results

### 4.1 Manual gating strategy to identify major NK cells subsets in the periphery and liver

The manual gating strategies used to define NK cells in PBMCs and liver samples are illustrated in **Figures 2A, B**. First several cleaning gates were applied: gating on lymphocytes using FSC-A/SSC-A, exclusion of doublets by gating on FSC-A/FSC-H, followed by RBC exclusion gate using the SSC-A and SSC-B-A parameters, gating on viable leucocytes and finally, exclusion of B cells/monocytes/pDCs/ granulocytes and T cells. Further gating was performed by gating on **1)** CD94^+/-^CD127^dim/-^ as described in (64, 65) and subsequently **2)** by including only CD56^+/-^HLA-DR^dim^ events and **3)** gating total NK cells based on CD56^+/-^CD16^+^ events.

### 4.2 Subsets and phenotypes of peripheral NK cells

The gated total NK cells in PBMCs were classified into 3 subsets by means of CD56 and CD16 expression levels. Using this gating strategy, we clearly identified the three classical subsets described in PBMCs, namely early, mature, and terminal NK cells as illustrated in **Figure 2A**. Further details of the expression profiles of NK cell markers in the three main subsets are also shown in **Figure 2A** in which the antigens are classified according to the functional properties of the markers. Early NK cells were enriched for expression of the inhibitory receptor CD159a, expressed high levels of the activating receptor CD335 and low levels of granzyme B and were negative for perforin. Expression of CD158b, a-h, as well as CD57 was absent or too low to be resolved, indicating the absence of clonal expansion and maturation respectively. The pattern of CD11b and CD27 expression showed enrichment of the CD11b^+^CD27^+^ population, a population described to be the most efficient in secreting cytokines in early NK cells (39). Furthermore, early NK cells were enriched for CD161 expression when compared to the other NK cell subsets, the engagement of which inhibits cytotoxicity and triggers IFN-ɣ production (66, 67). Adding another nuance to the definition of the functional properties of early NK cells was the detection of a subset expressing high levels of CD183. Interestingly, this phenotype has been described as producing higher levels of IFN-γ and having higher degranulation capacity (68).

In accordance with previous publications, mature NK cells expressed CD57 and CD158b, a_h, indicating maturation and clonal expansion, and higher levels of CX3CR1 as compared to early NK cells (5, 16, 40, 69). The cytotoxic molecules Granzyme B and Perforin were highly expressed as compared to early NK cells as described before (3, 5, 69). However, expression patterns of CD57/CD161, CD159a/CD335, TIGIT/CD158b, a_h/CD85j, CD11b/CD27, and TIGIT/CD159c could indicate the presence of different phenotypes within mature NK cells (**Figure 2A**). Additionally, some mature NK cells coexpressed CD186 and CD69 and were found in all 5 donors and were also observed in early NK cells. These CD69^+^CD186^+^ NK cells, detected in both the periphery and the liver, might represent a rare subset of NK cells with liver/tissue homing capacity (37, 70). The panel also captured a mature NK cell subset with enhanced effector responses and clonal expansion: the so-called adaptive NK cells that are CD56^dim^CD16^+^CD159c^+^CD159a^-^CD57^+^CD161^+^PLZF^low^CD85j^+^CD158b, a_h^+^, a phenotype enforced by HCMV exposure and detected at higher frequencies in HCMV-positive individuals (16, 41, 42, 71–74). The sample used as an example of the panel performance in **Figure 2A** shows the expression of CD159c in mature NK cells that, with the markers present in the panel, could potentially be further defined in terms of the expression of markers associated with adaptive NK cells. Terminal CD56^-^CD16^-^ NK cells are believed to be derived from mature NK cells with interchangeable phenotypes reflected by modulation of CD159a and KIRs expression (9). Those cells express higher levels of KIRs, CD57, CD85j, TIGIT, lower levels of the activating receptors CD314 and CD335 and expand with age and under chronic infection or lymphopenic conditions like hematopoietic stem cell transplantation (HSCT (75–78). Terminal NK cells, although dysfunctional in terms of expressing lower levels of cytotoxic molecules and presenting a lower responsiveness to cytokines, can still exert cytotoxicity through other mechanisms like ADCC or death-receptor mediated cytotoxicity. As expected, we detected terminal NK cells at low frequencies in the PBMC of all donors with variable expression patterns of CD159a, KIRs, CD57, CD314, CD335 as well as TIGIT and CD85j. In terms of transcription factors (TF), the expression of Eomes was higher in early NK cells as compared to mature and terminal NK cells related to the different roles these TFs have in the process of NK cell maturation (79).

### 4.3 Subsets and phenotypes of liver NK cells

Liver NK cells are divided into two main subsets based upon CD56 and CD16 expression, namely the CD56^bright^CD16^-^ and CD56^dim^CD16^+^ subset. In the healthy liver, the two subsets are present at equal frequencies in contrast to the distribution seen in PBMCs (18, 34). The CD56^bright^CD16^-^ subset has been further defined as CD56^bright^CD16^-^CD69^+^CD186^+^Eomes^Hi^Tbet^low^PLZF^Hi^TIGIT^+^CD49e^-^CX3CR1^-^ (23, 31, 33–37, 80, 81) and are called tr-NK cells. Tr-NK cells possibly originate from peripheral CD56^dim^CD16^+^CD69^+^CD186^+^Eomes^low^Tbet^hi^PLZF^hi^ NK cells (35, 37). The CD56^dim^CD16^+^ subset represents phenotypes very similar to peripheral mature NK cells as they are mainly NK cells recirculating from the periphery and annotated as transient conventional NK cells (cNK). The cNK subsets express CD49e high levels of T bet and low levels of Eomes in contrast to tr-NK cells (18, 31, 33). Within the tr-NK and cNK subsets, additional populations are distinguished based upon CD159c expression with CD159c^+^ NK cells representing adaptive NK cells prone to respond upon viral rechallenge (18, 26, 36). Given this information, we based the manual gating strategy upon expression levels of CD56 and CD16, after gating on CD94^+/-^CD127^+/-^CD56^+/-^HLA-DR^dim/-^ events. **Figure 2B** shows that liver NK cells can be clearly divided into the two described main populations of tr-NK and cNK with a near equal distribution in terms of frequencies. Most CD69^+^CD186^+^ liver NK cells are found in the CD56^bright^CD16^-^ subset, the latter also being predominantly Eomes^hi^Tbet^low^CD49e^-^CX3CR1^-^. In 2 out of 3 HD livers, we observed adaptive NK cells in both the CD56^bright^CD16^-^ and CD56^dim^CD16 subsets of which one example is shown in **Figure 2B**. In this example, the adaptive NK cells expressed high levels of the inhibitory receptor CD85j and checkpoint inhibitor TIGIT, as also observed in adaptive NK cells present in PBMCs (**Figure 2A**). Additional markers showed even more granularity within the three populations of tr-NK, cNK and liver adaptive NK cells. For example, CD49a and CD103 were detected at higher frequencies in the gated tr-NK subset which both are markers of tissue residency. Furthermore, CD11b^+^CD27^+^ NK cells were enriched in the tr-NK subset and displayed a dominant expression of the inhibitory receptors CD159a, CD161 and low to no expression of CD57 and CD158b, a_h as compared to cNK cells. As described previously (31, 36), liver NK cells (tr-NK, cNK and adaptive NK cells) expressed low levels of perforin as compared to peripheral NK cells and most tr-NK cells did not express granzyme B. Additionally, we confirmed the dominance of Eomes over Tbet expression and strong PLZF expression in tr-NK cells as compared to cNK (**Figure 2B****),** reflecting the role of Eomes and PLFZ in enforcing a tissue-resident phenotype (35, 37).

### 4.4 Additional phenotype information derived from multidimensional analysis

**Figure 3:**
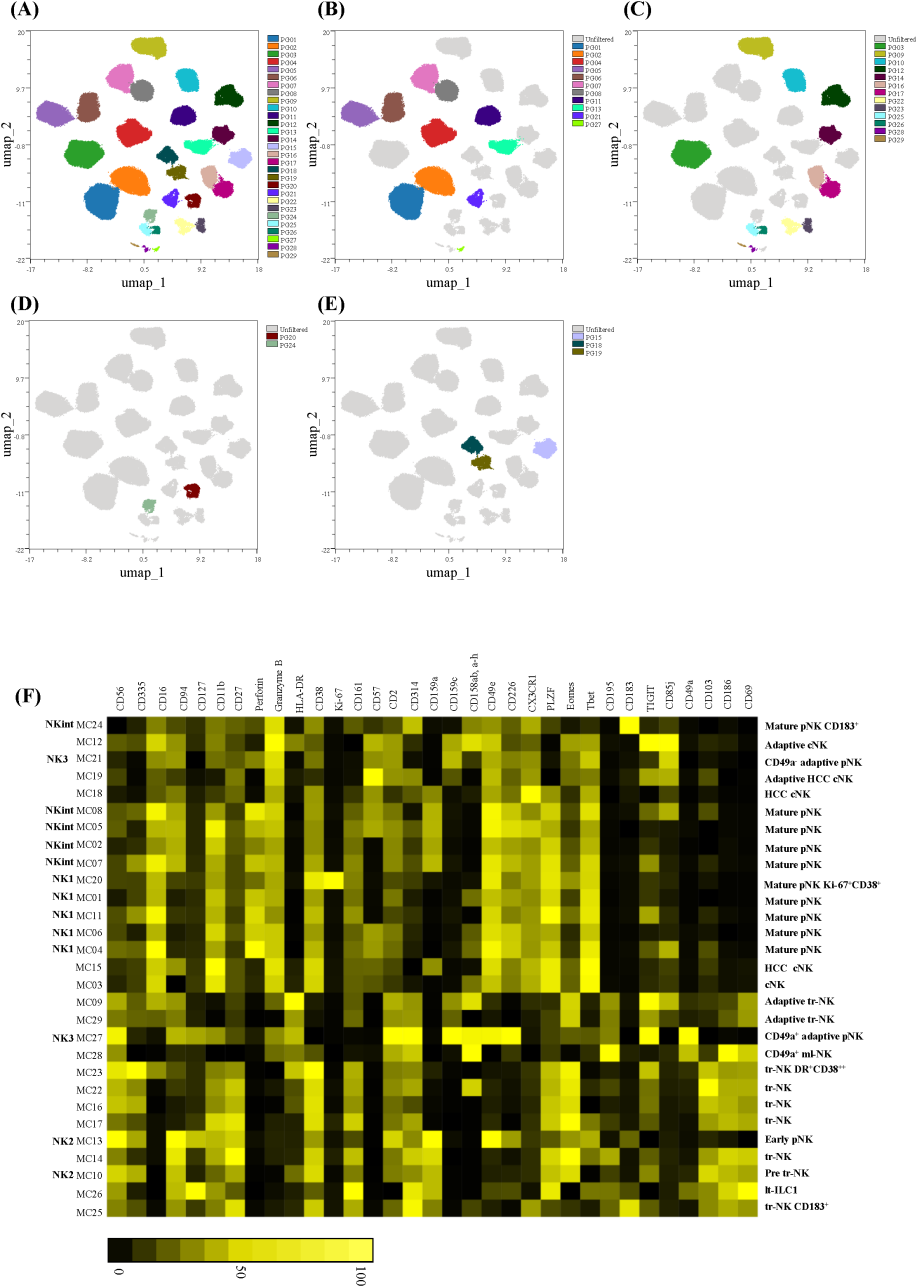
Multidimensional analysis of NK cells. Data were analyzed using the OMIQ platform with the analysis pipeline as described in the methods section. Phenograph clusters are overlayed on the concatenated UMAP of all samples for ease of visualization as in **(A)** all clusters identified, **(B)** clusters enriched in PBMCs, **(C)** clusters enriched in HD Liver, **(D)** clusters shared between PBMC and HD Liver and **(E)** clusters enriched in HCC Liver samples. **(F)** Heat-map generated of the different clusters and ordered hierarchically. The annotation of each metacluster (MC) is shown on the left with the annotation of NK1, NK2, NK3 and NKint. Annotation according to early, mature, adaptive peripheral (pNK; CD49a^-^ or CD49a^+^), pre tr-NK, tr-NK, CD49a^+^ ml-NK, adaptive cNK, cNK, and lt-ILC is indicated on the right with indication of a distinct feature of the cluster when applicable. Marker intensity is depicted from black (negative) to yellow (positive) on a scale from zero to 100 percentile based on intensity per column.

While manual gating is commonly used to demonstrate the ability to resolve populations of interest, it is generally not practical, time consuming, and can lead to a biassed and incomplete phenotyping of the samples when working with 37 parameters. Therefore, we proceeded with multidimensional analysis of total NK cells (early, mature and terminal) as outlined in **Figures 2A, B**, by applying the Phenograph clustering algorithm followed by the UMAP reduction algorithm. For the multidimensional analysis, we included two additional HCC liver samples. Including HCC liver samples allowed us to verify the use of the panel not only in health but also in disease state, in particular liver cancer. The gating strategy applied to define total NK cells from liver samples with HCC is the same as shown in **Figure 2B**. Applying the clustering algorithm to 5 PBMC donors, 3 HD liver and 2 HCC liver identified a total of 29 clusters. Before proceeding further with the analysis of cluster identity and dynamics, the significance of each cluster was verified by setting the following requirements: each cluster needed to consist of at least 100 events and/or be present in at least 3 samples, either in PBMC or liver. All clusters fulfilled these requirements (depicted in **Supplementary Table 4**), and we therefore proceeded with the assignment of clusters to phenotypes and/or known populations. The 29 identified clusters are illustrated in **Figure 3A** overlayed on the concatenated UMAP of all 10 samples.

### 4.5 Cluster tissue-prevalence

One of the purposes of the panel design was to be able to distinguish peripheral and tissue (liver) specific NK cells. We therefore first determined the prevalence of clusters in each sample type, as percentages of total NK cells. Our data indicated that certain clusters were either more prevalent in PBMCs as compared to HD liver (clusters 1, 2, 4-8, 11, 13, 21 and 27; **Figure 3B**) or in HD liver as compared to PBMCs (clusters 3, 9-10, 12, 14, 16-17, 22-23, 25-26, and 28-29; **Figure 3C**). Other clusters were shared between PBMCs and liver samples (clusters 20 and 24; **Figure 3D**), at equal percentages of total NK cells. Three clusters were prevalent in HCC liver (clusters 15, 18 and 19; **Figure 3E**) and a detailed analysis of the biological significance of these clusters is provided in section 4.6.

Given the reported dynamic phenotype of NK cells based upon environmental cues (vaccination/infection history, tissue health status) and genetics (12, 82–86) we determined donor-dependent prevalence of each cluster. The percentages of each cluster of total NK cells, per individual PBMC or liver donor is displayed in **Supplementary Figure 10** and events assigned to each cluster in **Supplementary Table 4**. This analysis shows the dominance of certain clusters in the periphery versus liver but also donor-dependent differences. For example, in the case of clusters 4 and 8 that are more prevalent in PBMCs, cluster 4 was nearly absent in PBMCs of donor 3 (1.3 %) and cluster 8 was detected at low percentages in PBMCs of donors 1, 2 and 3 (respectively 2.4%, 2.5%, and 1.4%). Clusters 9 and 10, two out of 13 clusters that were more prevalent in HD liver, represented more than 15% of total NK cells in donors 1 and 3 but were hardly detectable (<1%) in donor 2. Clusters that were prevalent in the HCC liver represented 46.5% of total NK cells for cluster 15 in HCC donor 1 (1.3% in HCC donor 2) and 44.6% for cluster 18 (1.2% in HCC donor 1) and 37.6% for cluster 19 in HCC donor 2 (0% in HCC donor 1). These three clusters were present at a low percentage in both HD liver and PBMC. Testing of the HCC liver samples was performed as proof of concept of the utility of this panel although we acknowledge the limitations of sample size.

### 4.6 Cluster assignment and verification

Next, we proceeded with displaying the identified clusters hierarchically by means of a heatmap for the purpose of cluster assignment and verification (**Figure 3F**). To facilitate further data exploration, UMAP color-continuous scatterplots of each NK cell marker in the panel are presented in **Supplementary Figure 11A**. The extended phenotype of each cluster is shown in **Supplementary Figure 11B** as overlayed scatter plots displaying the expression of each marker defining the metaclusters (left) and the position of the cluster on the UMAP (right). The final assignment of different NK cell clusters, elaborated in detail in the section below, is indicated on the left of the heatmap in **Figure 3F** and are annotated as peripheral early NK cells (early pNK), peripheral mature NK cells (mature pNK), peripheral adaptive NK cells (adaptive pNK either CD49a^+^ or CD49a^-^ ), liver tr-NK, adaptive/memory liver resident NK cells (ml-NK), liver circulating NK (cNK), liver circulating adaptive NK (adaptive cNK), tr-NK precursor (pre tr-NK), liver circulating NK cells or adaptive cNK prevalent in HCC (HCC cNK, adaptive HCC cNK) and Lt-ILC1s. A summary of peripheral and HD liver NK cell subsets described in the literature is given in **Supplementary Table 5** that lists core markers identifying the subsets, markers enriched or expressed on additional subsets/phenotypes, their reported frequencies and specific function/properties. The information listed was used as a guide for the final Phenograph cluster assignments which is also indicated in **Supplementary Table 5**.

From the heatmap it could be deduced that the three main NK cell subsets in the periphery were represented in cluster 13 for early NK cells (CD56^bright^CD16^-^CD69^+^CD186^+^CD335^bright^CD127^+^CD11b^+^CD27^+^CD183^+^CD159a^+^CD159c^-^CD161^low^CD57^-^CD158b, a_h^-^CD49e^+^CX3CR1^-^Eomes^low^Tbet^low^) and in clusters 1-2, 4-8, 11, 20 and 24 for mature non-adaptive NK cells (CD56^dim^CD16^-^CD69^-^CD186^-^Eomes^Int^Tbet^+^PLZF^+^CD127^-^CD11b^+^CD27^dim^CD49e^+^CX3CR1^+^CD226^+^). Mature NK cell clusters had variable expression levels of CD335, CD94, CD161, CD57, CD158b, a_h. Some non-adaptive mature NK cells were distinctive in expressing high levels of CD183 (cluster 24) or high levels of Ki-67 and CD38 (cluster 20). No specific cluster(s) could be assigned to terminal NK cells in agreement with their described highly variable phenotype (9). NK cells that corresponded to the described peripheral “classical” adaptive NK cells (CD49a^-^CD127^-^ CD56^dim^CD16^+^CD159c^+^CD159a^-^CD57^+^CD2^+^CD161^-^CD49e^+^CX3CR1^+^CD69^-^CD186^-^Eomes^+^Tbet^+^PLZF^low^CD158b, a_h^+^Perforin^+^GranzymeB^+^CD85j^+^) resided in cluster 21 and were detected in the PBMCs of donor 3 (24.8%) and donor 5 (2%).

Tr-NK cells, defined as CD56^+^CD16^-^CD57^-^CD69^+^CD186^+^Eomes^hi^Tbet^low^PLZF^bright^CD2^+^CD49e^-^CX3CR1^-^CD226^-^, were clearly distinguished from the rest of the clusters in the heatmap and resided in clusters 10, 14, 16-17, 22-23, 25. These clusters could be distinguished by different expression levels of CD94, absence or presence of CD159a, and differences in expression levels of CD335, CD195, HLA-DR. All tr-NK cells were enriched for TIGIT and CD103 expression as described previously (31, 35, 81). Additionally, all tr-NK clusters were CD11b^+^CD27^+^CD161^bright^CD38^bright^Perforin^-^GranzymeB^-^ indicating that tr-NK cells have less cytotoxic capacity but might have enhanced pro-inflammatory potential as described for peripheral CD11b^+^CD27^+^NK cells (39). The absence of CD49e, CX3CR1 and increased CD195 expression indicated tissue residency, as described for tr-NK cells (23, 31, 33). Overall, the cluster designations of tr-NK cells were in accordance with their previously described phenotype.

**Figure 4:**
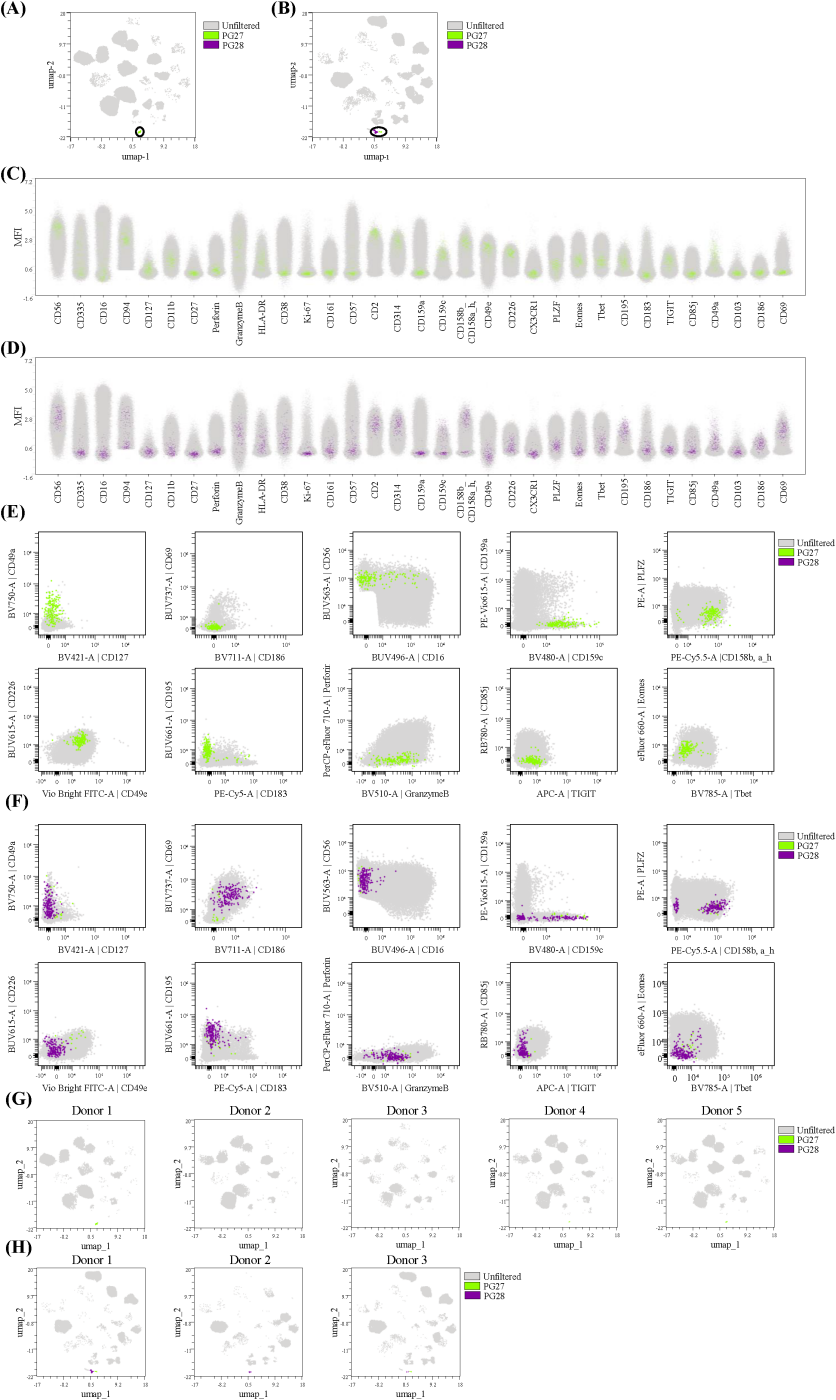
Detailed cluster verification. Verification of clusters 27 and 28 was performed by **(A)** superimposing the color-coded clusters (green; cluster 27, purple: cluster 28) on the concatenated UMAPs (grey; unfiltered) of PBMCs and **(B)** HD liver samples. Displaying the expression levels of all NK cell markers in **(C)** cluster 27 and **(D)** cluster 28 by superimposing the color-coded clusters on all concatenated files (grey; unfiltered). Generating biexponential plots of the color-coded clusters with markers unique and/or differentially expressed between the two clusters as indicated in the heatmap and superimposed on **(E)** concatenated PBMCs and **(F)** concatenated HD liver samples. Superimposing the color-coded clusters on the UMAP per individual donor in **(G)** PBMC and **(H)** HD liver samples to confirm tissue-specificity and donor-dependency of each cluster.

As CD49a has been described to identify a liver-specific NK cells subset (36) and because the frequency of intrahepatic CD49a^+^ NK cells has been shown to be associated with tumor progression and clinical outcome in HCC (87) we analyzed the CD49a^+^ clusters in further detail. Two clusters expressed high levels of CD49a, namely clusters 27 and 28, one being more prevalent in PBMCs (cluster 27) and the other one in HD liver (cluster 28). We then proceeded with a detailed cluster verification of cluster 27 and 28 in several manners. First, we visualized the prevalence of the clusters per sample type by superimposing the location of the 2 clusters on concatenated UMAPs for PBMC (**Figure 4A**) and HD liver samples (**Figure 4B**). Additionally, the expression of all NK markers in each cluster is illustrated as color-coded clusters superimposed on total NK cells (unfiltered) of all concatenated files in PBMCs (**Figure 4C**) and HD liver (**Figure 4D**). Furthermore, we generated bi-exponential plots of selected markers defining the clusters, either on total NK cells (unfiltered; grey) of concatenated PBMC (**Figure 4E**) or concatenated HD liver samples (**Figure 4F**) with the color-coded clusters superimposed. UMAPs of each PBMC donor (**Figure 4G**) or HD liver donor (**Figure 4H**) visualize the presence of the clusters per individual sample and donor-dependent variety. The phenotype of cluster 28 was confirmed as being CD56^bright^CD16^-^CD69^+^CD186^+^Eomes^low^Tbet^low^PLZF^low^CD159c^+^CD158b, a_h^+^CD49e^-^CX3CR1^-^CD226^-^CD195^+^CD183^-^TIGIT^-^CD85j^dim^ (**Figures 4C–F**) and represents the described CD49a^+^ liver specific ml-NK subset (36). The UMAPs in **Figures 4G, H** confirmed that cluster 28 is only present in HD liver. Cluster 27 resembled peripheral early NK cells being CD56^bright^CD16^-/low^CD127^low^CD69^-^CD186^-^Eomes^int^Tbet^int^PLZF^low^CD158b, a_h^+^CD49e^+^CX3CR1^-^CD226^+^CD195^+^CD183^-^TIGIT^+^CD85j^-^ (**Figures 4C–F**) but additionally expressed CD159c. As such, this cluster corresponded to the described CD56^bright^CD16^-^ adaptive NK cells, detectable in blood and tissues, with features of tissue-residency (88). Accordingly, cluster 27 was detected at low frequencies in both PBMC (3 out of 5 donors) and in HD livers (2 out of 3 donors) (**Figures 4C, D****).** We confirmed the presence of cluster 27 in three independent experiments in the PBMC of donor 1 (data not shown) for additional validation.

Additional verification of two clusters (clusters 9 and 10) with a similar phenotype as tr-NK cells is presented in **Supplementary Figure 12**. Both clusters were CD49a^-^ and CD69^+^CD186^+^Eomes^hi^Tbet^low/+^CD49e^-^CX3CR1^-^CD226^-^, with cluster 9 being CD159c^+^ and cluster 10 being CD159c^-^. **Supplementary Figures 12C-F** confirmed the differential expression of CD159c between the two clusters and high expression of CD158b, a_h, TIGIT, CD85j and HLA-DR and low expression of PLZF in cluster 9. Although the phenotype of cluster 9 was similar to the described peripheral CD56^dim^CD16^+^ adaptive NK cells this cluster displayed an expression profile reminiscent of tissue residency (CD69^+^CD186^+^Eomes^hi^Tbet^low^CD49e^-^CX3CR1^-^CD226^-^), expressed Granzyme B but not perforin, was negative for CD57, and was HLA-DR^hi^ thereby indicating activation. Cluster 9 was detected in all HD liver donors, but only at low percentage in 2 out 5 PBMCs (**Supplementary Figures 12G, H**). Given the expression of markers of tissue residency and despite the expression of CD16, we designated this cluster, as well as cluster 29, as adaptive tr-NK cells. Cluster 10 was further defined as CD56^+^CD16^-^CD57^-^CD69^+^CD186^+^Eomes^hi^Tbet^low^PLZF^bright^CD49e^-^CX3CR1^-^CD226 (**Supplementary Figure 12D-F**). Cluster 10 was also detected at low percentages in the PBMCs of all 5 donors (**Supplementary Figure 12G-H**) and resembles the described peripheral precursors of tr-NK cells (pre tr-NK) (35, 37, 70). We identified cluster 10 in PBMCs of three independent acquisitions in donor 3 (data not shown) which reinforced the validity of this cluster. Notably, cluster 9 and other liver adaptive NK cells (clusters 12 and 29) all expressed higher levels of TIGIT as well as lower levels of CD226 than CD49a^-^ adaptive NK present in PBMCs (cluster 21). The pattern of TIGIT and CD226 expression might contribute to a unique role of liver adaptive NK cells in maintaining immune homeostasis as previously suggested (81). Additionally, different expression levels of CD158b, a_h were observed among liver adaptive NK cells which might be due to differences in clonal expansion related to HCMV exposure (26).

An important adjustment to our panel design and gating strategy was to include CD127 and CD94. This adjustment was made to distinguish between NK cells (CD127^+/-^CD94^+^) and ILCs (CD127^+^CD94^-^). However, CD49a^+^ lt-ILC1 were recently described, with a phenotype similar to tr-NK cells, including CD94 expression (32). Lt-ILCs could be further identified by expression levels of NKp80 (negative on lt-ILC1s) and Eomes (negative to low on Lt-ILC1s) and being CD200R1^+^CD127^low^CD161^Bright^. Despite the absence of NKp80 in our panel, we were able to identify lt-ILCs in cluster 26. In line with the description of lt-ILC1s, cluster 26 was enriched for CD49a expression and was CD127^+/dim^CD56^bright^CD16^-^CD94^+^CD57^-^CD69^+^CD186^+^CD94^+^CD161^bright^CD158b, a_h^-^Eomes^low^Tbet^+^PLZF^bright^CD2^-^CD49e^low^CX3CR1^-^CD226^low^ (**Figure 3F**). Additionally, cells in cluster 26 were CD183^+^ and contained heterogenous expression of CD103^+^ cells as in (32) and expressed CD195, two markers of tissue residency. As CD183 plays a role in tissue homing of NK cells under homeostatic conditions and aids in recruiting NK cells to diverse tumor and inflammatory environments (89), this observation suggests that CD183 might also aid the localization  of lt-ILCs in combination with other tissue-homing receptors.

In HCC liver samples, derived from the tumor site, we found three clusters to be highly prevalent (clusters 15, 18, 19**;****Figure 3E**) as compared to HD liver and PBMCs. These clusters were CD56^dim^CD16^+^CD69^-^CD186^-^CD49e^+^CX3CR1^+^, and did not express markers of tissue residency, which indicated that these three clusters were cNKs. The HCC liver prevalent clusters represented respectively adaptive NK cells in cluster 19 (37.6% of total NK cells in HCC liver donor 2; CD56^dim^CD16^+^CD314^+^ CD159a^-^CD159c^+^CD158b, a_h^-^CD161^-^CD159a^-^CD57^Hi^GranzymeB^+^Perforin^-^TIGIT^+^CD85j^+^), and mature NK cells in both cluster 18 (44.6% of total NK cells in HCC liver donor 2; CD56^dim^CD16^+^CD159c^-^CD158b, a_h^-^CD161^+^CD314^+^CD159a^+^CD159c^-^CD57^+^GranzymeB^+^Perforin^-^TIGIT^-^CD85j^-/low^) and cluster 15 (46.5% of total NK cells in HCC liver donor 1; CD56^dim^CD16^+^CD159c^-^CD158b, a_h^-^CD161^+^CD314^-^CD159a^+^CD159c^-^CD57^+^GranzymeB^+^Perforin^-^TIGIT^-^CD85j^-^). Notably, cluster 15 was distinct from the clusters representing peripheral mature NK by their low expression of the NK cell activating receptors CD314 and CD226 and their high levels of the inhibitory receptor CD159a. This combination of NK cell receptors suggests that NK cells belonging to cluster 15 have impaired functionality, as already described in HCC (30, 48, 90). Cluster 19 was CD159c^+^ and distinct from adaptive NK cells prevalent in the periphery (cluster 21) or HD liver (cluster 12) as they expressed high levels of CD57 and CD314 and higher levels of the checkpoint inhibitory molecule TIGIT and the inhibitory receptor CD85j as compared to cluster 21. Cluster 18 was similar to the mature NK cells prevalent in PBMCs (cluster 8), with the distinction that cluster 18 was negative for perforin, TIGIT and CD85j, and express lower levels of CD226, CD335, and CD2 and higher levels of CD314. As such, cluster 15 and 18 expressed a mixture of molecules involved in either NK cell activation or dysfunction. We did not detect CD49a^+^ NK cells in the two HCC liver donors analyzed, in contrast to their reported increased frequency in HCC patients with poor prognosis (87, 91). This might be due to the limited number of samples included in the analysis.

Interestingly, NK cells were recently reclassified by means of scRNAseq and CITE-seq into 6 subsets, namely NK1A-C, NKint, NK2, and NK3 (16). These subsets represent mature NK cells with different metabolic activity (NK1A-C), early NK cells (NK2), adaptive NK cells (NK3) and a NKint subset that is transitional between NK2 and NK1C. These subsets were detected in different tissues and tumor samples, including HCC, although NK cells in healthy liver samples were not analyzed and markers ascribed to tr-NK cells were not included. With our panel, we were able to identify subpopulations resembling NK1, NK2, NK3, and NKint subsets in PBMCs based on expression levels of CD56, CD16, CD159C, CD159a, CD335, CD314, CD158b, a_h, CD183, PLZF and TIGIT. NKint were described as expressing lower levels of CD335, CD56, CD158_ah, intermediate levels of CD159a, TIGIT and high levels of CD183. Therefore, we annotated clusters 2, 5, 7–8 and 24 as NKint using a combination of these markers. NK2 were defined as being CD56^bright^CD16^-^CD127^+^CD159a^+^CD159c^-^CD183^dim^ and were assigned to clusters 10 and 13. NK1 were assigned to clusters 1, 4, 6, 11 and 20 based on the described absence of CD159a and CD159c as well as variable expression of CD158b, a_h and TIGIT. Finally, clusters 21 and 27 matched the phenotype of NK3, as they were CD159c^+^CD159a^-^TIGIT^+^PLZF^-^ as described (16). Designation of clusters according to this newly proposed classification in PBMCs is indicated on the right of the heatmap in **Figure 3F** and in **Supplementary Table 5**.

## 5 Discussion

We present in this method paper the development of a high dimensional full spectral flow cytometric human NK cell panel that can **1)** identify all the NK cell subsets that have been described as present in the periphery and liver; **2)** identify nuances in the different NK cell subsets described as present in the periphery and liver obtained from healthy donors; **3)** distinguish NK cells from ILCs and lt-ILCs; **4)** be used for samples with limited cell numbers and NK cell frequencies; **5)** identify NK cell phenotypes prevalent in health and disease and; **6)** resolve each marker optimally in this high dimensional application focused on one leucocyte lineage. As such, this panel is a valuable tool for NK cell phenotyping in the liver and in the periphery under different pathological conditions. It is likely that this panel can also be used for phenotyping of NK in other sample (tissue) types if the same digestion protocol is applied.

We based the design of this panel on two published panels designed for conventional platforms with key improvements to the gating strategy and panel design. Well established metrics for panel design were used, namely Similarity Index (51), SSM (92), and antigen classification (53). All reagents used in the panel were titrated to ensure optimal target identification and tested for digestion sensitivity. Additionally, we verified the final panel performance by confirming optimal resolution of each marker on both PBMC and liver samples using previously published strategies (51, 52). Despite the limited number of PBMCs and liver samples tested, we confirmed that our panel was able to identify described peripheral and liver (tr-NK and c-NK) NK cells, lt-ILC1s and clusters prevalent in the liver of HCC donors A key improvement that was made in this panel is the inclusion of CD94, HLA-DR and CD127. All NK cells are CD94^+^ which can distinguish them from peripheral CD94^-^ ILCs and can additionally define functional NK cell subsets (93). More importantly, CD127 combined with expression levels of CD94, Eomes and CD49a, allowed us to distinguish tr-NK cells from the recently described CD94^+^ lt-ILC1s (32). Although NKp80 has been designated as a key marker to distinguish NK cells from ILCs and lt-ILC1s (32, 94), we showed that NKp80 is sensitive to our enzyme digestion protocol and noted that this limitation has also been described for commercially available enzyme cocktails. Despite this shortcoming, our panel design provides an alternative gating strategy allowing the distinction between NK cells, ILCs and lt-ILC1s in case enzymatic digestion of samples is needed. Additionally, the inclusion of CD94, instead of using CD127 as unique exclusion marker for classical ILC subsets (11, 64, 65, 95, 96), allowed us to better define the phenotype of early NK cells as being CD56^bright^CD16^-^CD127^+^CD94^bright^CD183^+^, as described by a variety of technologies (65, 69, 97), and distinguishing them from the rare CD127^-^ peripheral precursors of tr-NKs (35, 37, 70). By using CD127 in combination with CD49a we also identified the CD49a^+^ adaptive NK cell subset present a low frequency in both liver and PBMCs. HLA-DR is often used as an exclusion marker when analyzing NK cells (11, 98, 99). However, it has now been clearly documented that HLA-DR^+^ NK cells represent NK cells with enhanced effector function (100, 101). By including the CD94^+^CD56^+^HLA-DR^dim^ population in our gating strategy, we confirmed that HLA-DR expression is confined to specific clusters, namely clusters representing adaptive cNK, adaptive tr-NK, CD49a^+^ adaptive pNK, CD49a^+^ ml-NK, pre-tr-NK and CD49a^-^ tr-NK cells. Possibly, these HLA-DR^+^ clusters exert enhanced effector functions like increased production of proinflammatory cytokines or other granzymes and/or enhanced antigen presentation (100).

Although we acknowledge that the number of HCC liver (n=2) and HD liver (n=3) samples used in this study is limited, it was important to confirm that the panel performed optimally on these sample types, as well as to validate the performance of markers associated with NK cell dysfunction. The fact that for HCC liver the cluster distribution was very different between the two donors is not surprising due to the diverse etiology of the disease. Additionally, cancer stage, specific tumor location sampled, treatment strategy, viral infection history, age, gender and other factors could influence the NK cell clusters found in each donor and would need expansion to a larger cohort with clear patient/sample stratification in order to correlate NK cell clusters with patient/sample specific features. However, the results of our study constitute proof of concept of the usefulness of all the markers included in our panel in the context of liver cancer. We incorporated several markers with clinical relevance that affect NK cell functional status including two immune checkpoint molecules, TIGIT and CD226. TIGIT is an inhibitory receptor that decreases NK cell cytotoxic capacity and is upregulated on liver tr-NK. CD226 is an activating receptor mediating anti-tumor responses through recognition of its ligands that are upregulated in tumor cells. Both TIGIT and CD226 share the same ligands, with TIGIT having a higher binding affinity. As such, the balance of TIGIT and CD226 expression levels can influence the function of NK cells. For example, dysfunctional NK cells that express TIGIT in combination with lower levels of CD226 have been observed in liver cancer, correlate with disease prognosis and are increased in the periphery of hepatitis B-related HCC patients (21, 43, 44, 81, 102–105). In addition, other molecules that have been linked to decreased NK cell function like CD85j, CD161, CD159a and CD314 were also included. CD85j is an inhibitory NK cell receptor of which one of the ligands is a viral ligand encoded by HCMV (106). CD85j identifies dysfunctional NK cells in chronic HBV and HCMV infection (72, 73, 107, 108), conditions often associated with the development of liver cancer. We detected CD85j on liver-specific adaptive NK cells, peripheral adaptive NK cells and subsets of peripheral mature NK cells, allowing to further define distinct functional subsets. CD161 is known to inhibit NK cytolytic function and is a potential target for immunotherapy of HCC (109, 110). CD314 is an activating NK cell receptor triggering NK cell cytotoxicity which downregulation has been correlated with a diminished anti-tumor response (48, 111–113). CD159a is an inhibitory NK cell receptor correlated with poor prognosis in liver cancer (21, 90). Notably, we found three NK cell clusters to be more prevalent in HCC liver samples with specific combinations and expression levels of markers associated with NK cell functionality. The clinical relevance of several markers included in this panel is further emphasized by a recent study in which the presence of NK cells with a specific expression profile (expressing higher levels of CD57, NKG2c, CD314 and CD335 and lower levels of TIGIT and NKG2a) could predict HCC recurrence risk and was related to the specific tumor location sampled (114).

Recently, subsets of NK cells were reclassified as NK1A-C, NK2, NK3 and NKint representing mature, early, adaptive and an intermediate stage between NK2 and NK1 cells (16). We started developing this panel before the publication of this NK cell reclassification and for this reason markers that could further distinguish between the different NK1 subsets (chemotaxis receptors; CXCR4, S1PR1, and SP1PR4) were not included in our panel. However, we were able to define NK1, NK2, NK3, NKint subsets based on the combination of expression levels of CD56, CD16, CD159c, CD159a, CD335, CD314, CD158b, a_h, CD183, PLZF and TIGIT. In summary, with this optimized panel we were able to accurately identify NK cell subsets previously defined in the literature in PBMCs of HDs and liver biopsies, as summarized in **Supplementary Table 5**. In the near future, it would be interesting to incorporate additional and newly described markers into this panel for a deeper characterization of newly defined subsets.

In conclusion, this is the first high dimensional spectral flow cytometric panel designed for in-depth characterization of NK cells, including 35 markers that can all potentially be coexpressed. We present data supporting the robustness and utility of this panel by providing data supporting the optimal performance of the panel and by showing the effective identification of human NK cell subsets/phenotypes previously described to be present in the circulation and in the liver. We believe that this panel could be a useful tool in studies aimed at understanding the dynamics of NK cells in health and disease states as well as at the development of NK cell-targeted immunotherapies.

## Data Availability

The original contributions presented in the study are included in the article/**Supplementary Material**. Further inquiries can be directed to the corresponding authors.
