# Supplementary material for: A 41-marker 37-color full spectrum flow cytometry panel for the deep immunophenotyping of human peripheral and liver natural killer cells
Source: Front Immunol. 2025 Dec 10;16:1609732. doi: 10.3389/fimmu.2025.1609732 (PMC12728072; doi:10.3389/fimmu.2025.1609732)
Supplement: Supplementary file 1 [file DataSheet1.pdf]

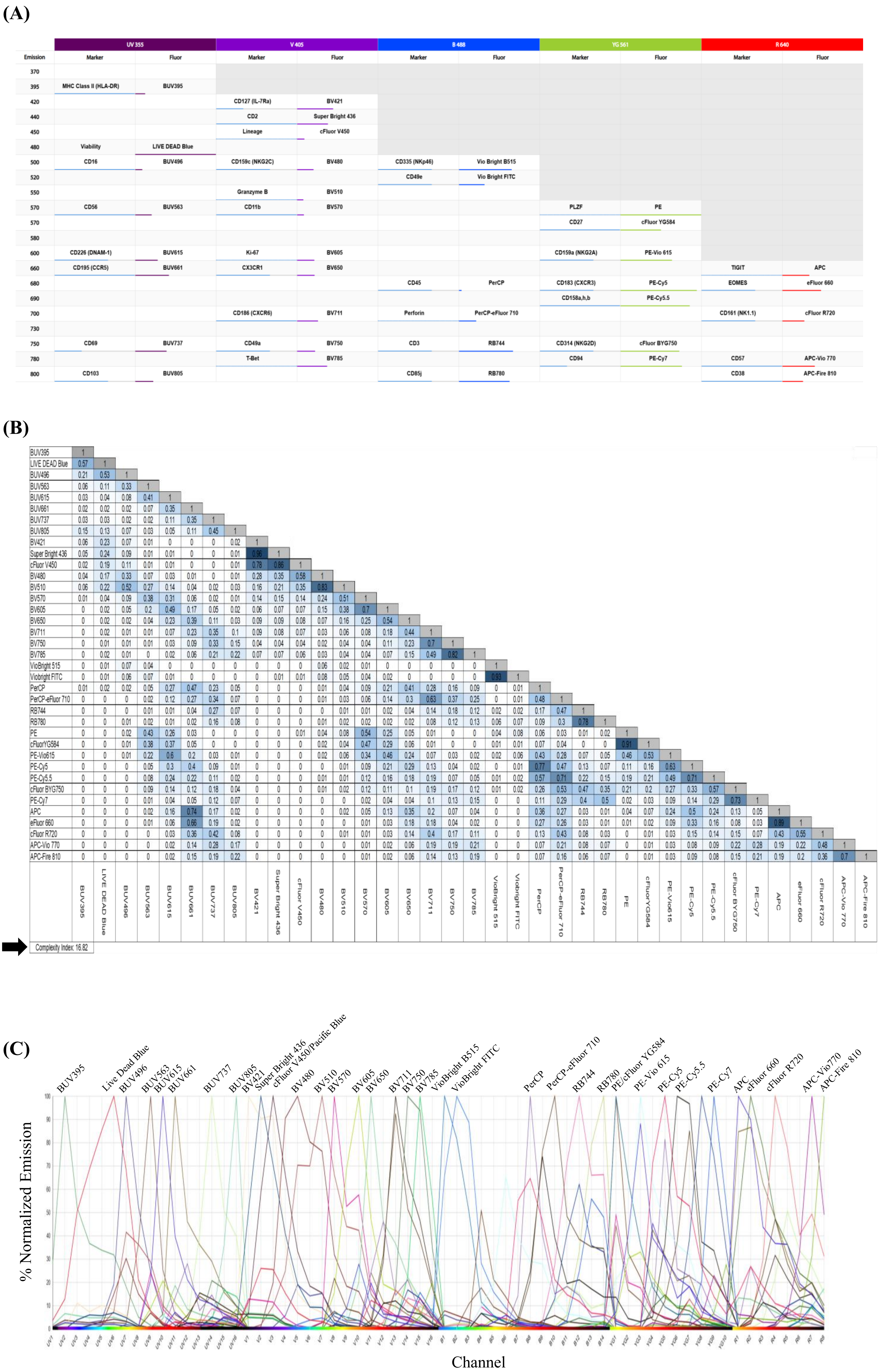

Supplemental Figure 1. Panel design.
(A) Fluorochrome and reagent optical layout. Assignment of fluorochromes to antigens was based on reagent availability, antigen density and co-expression levels. **(B)** The cosine similarity matrix of the panel that indicates how similar two spectra are to each other with values of "1" indicating no difference, while a value of "0" indicates that two fluorochromes are completely unique. The condition number (black arrow) of the panel indicates the complexity of the total combination of fluorochromes with the value 16.82. **(C)** Spectral signatures of the 37 fluorochromes visualized using the Cytek® Cloud Full Spectrum Viewer.

(A)

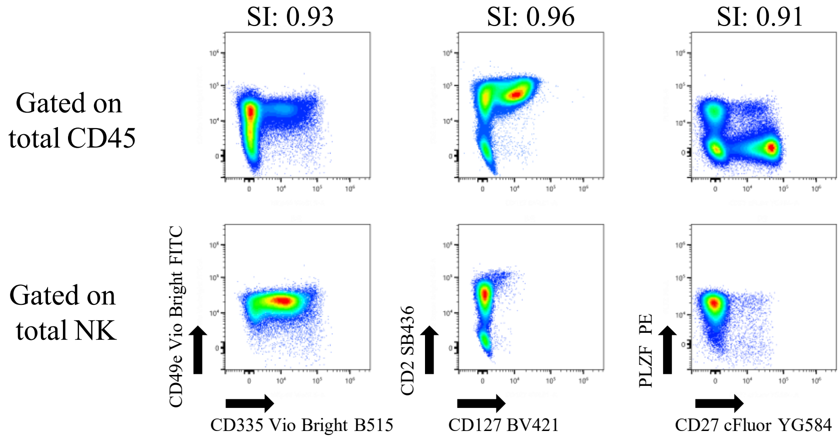

(B)

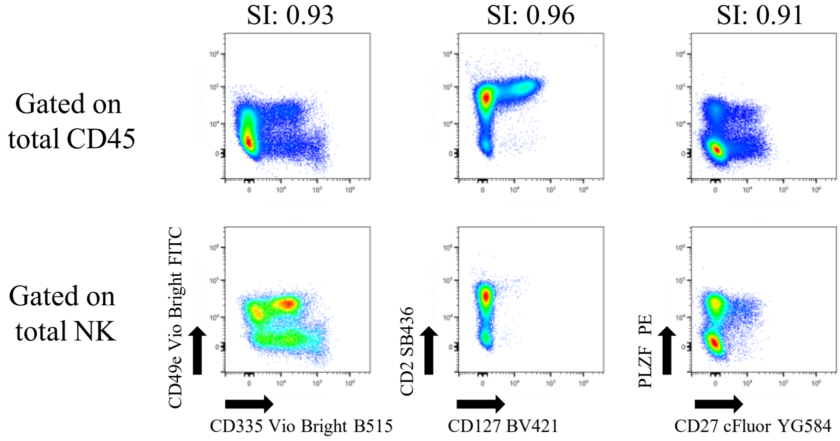

(C)

	BUV395	LIVE DEAD Blue	BUV496	BUV563	BUV615	BUV661	BUV737	BUV805	BV421	Super Bright 436	cFluor V450	BV480	BV510	BV570	BV605	BV650	BV711	BV750	BV785	Vio Bright B515	Vio Bright FITC	PerCP	PerCP-eFluor 710	RB744	RB780	PE	cFluor YG584	PE-Vio 615	PE-Cy5	PE-Cy5.5	cFluor BYG750	PE-Cy7	APC	eFluor 660	cFluor R720	APC-Vio 770	APC-Fire 810
BUV395	0.00	3.39	2.13	0.00	0.00	0.00	0.00	0.00	0.00	0.00	0.00	0.00	0.00	0.00	0.00	0.63	0.00	0.00	0.00	0.00	0.00	0.00	0.30	0.00	0.00	0.00	0.00	0.34	0.30	0.64	0.00	0.21	0.00	0.00	0.00	0.00	0.00
LIVE DEAD Blue	5.03	0.00	4.25	1.45	0.37	0.27	0.34	0.35	1.41	5.46	3.78	0.62	0.71	0.68	0.00	0.00	0.00	0.00	0.00	0.00	0.00	0.00	0.10	0.19	0.00	0.16	0.00	0.18	0.21	0.00	0.00	0.13	0.00	0.00	0.00	0.00	0.35
BUV496	0.55	0.51	0.00	1.95	0.82	0.44	0.45	0.46	0.26	0.00	0.00	0.87	1.21	0.90	0.46	0.50	0.00	0.38	0.39	1.15	0.87	0.13	0.14	0.00	0.00	1.18	0.19	0.37	0.21	0.00	0.00	0.00	0.30	0.00	0.13	0.00	0.15
BUV563	0.54	0.00	0.71	0.00	1.51	0.95	0.71	0.67	0.00	0.00	0.00	0.40	0.57	1.01	0.71	0.51	0.51	0.29	0.33	1.62	1.47	0.50	0.34	0.00	0.19	3.34	4.70	0.96	0.89	0.62	0.30	0.28	0.55	0.36	0.20	0.13	0.00
BUV615	0.55	0.00	0.00	1.67	0.00	2.24	2.11	1.85	0.36	0.00	0.00	0.17	0.00	0.78	2.23	0.84	1.28	0.58	0.60	0.59	0.52	1.31	1.35	0.77	0.56	5.91	5.29	3.04	2.07	2.11	1.10	1.00	1.34	0.92	0.77	0.22	0.39
BUV661	0.54	0.00	0.00	0.00	0.61	0.00	3.70	3.34	0.47	0.00	0.00	0.00	0.00	0.00	0.60	2.32	1.18	1.03	0.90	0.27	0.47	1.58	1.09	0.84	0.64	0.50	0.52	0.36	2.22	2.09	1.05	1.07	5.54	3.39	1.76	1.26	0.81
BUV737	0.69	0.31	0.00	0.00	0.17	1.02	0.00	7.58	0.36	0.58	0.00	0.00	0.00	0.00	0.24	0.48	1.62	2.17	1.60	0.00	0.50	0.52	1.35	2.36	1.86	0.43	0.00	0.00	0.60	0.97	0.70	0.86	1.38	1.43	3.94	2.05	1.32
BUV805	1.35	1.18	0.90	0.00	0.29	0.00	1.84	0.00	0.33	0.71	0.00	0.00	0.31	0.00	0.28	0.52	0.18	0.84	1.96	0.00	0.55	0.37	0.21	0.35	0.60	0.00	0.35	0.00	0.27	0.00	0.25	0.56	0.00	0.00	0.63	1.02	1.52
BV421	0.22	1.08	0.47	0.00	0.34	0.00	0.00	0.19	0.00	4.44	3.08	1.34	1.21	0.28	0.30	0.47	0.42	0.27	0.32	0.00	0.65	0.12	0.00	0.00	0.00	0.62	0.00	0.00	0.00	0.20	0.02	0.00	0.00	0.00	0.00	0.02	0.14
Super Bright 436	0.23	1.16	0.66	0.29	0.17	0.11	0.16	0.22	2.85	0.00	2.46	1.30	1.26	0.52	0.38	0.35	0.04	0.36	0.31	0.32	0.00	0.00	0.00	0.00	0.23	0.43	0.24	0.00	0.00	0.19	0.12	0.13	0.41	0.00	0.00	0.00	0.00
cFluor V450	0.00	0.55	1.05	0.00	0.31	0.21	0.00	0.45	2.22	3.88	0.00	1.36	1.63	0.81	0.78	0.70	0.55	0.27	0.48	0.00	0.57	0.21	0.00	0.00	0.25	0.00	0.00	0.00	0.24	0.00	0.00	0.00	0.28	0.00	0.00	0.33	0.00
BV480	0.00	0.73	4.16	1.02	0.47	0.35	0.16	0.18	0.71	1.29	1.10	0.00	2.77	1.73	1.26	0.75	0.40	0.43	0.43	1.35	1.12	0.12	0.18	0.00	0.23	0.76	0.35	0.25	0.13	0.00	0.00	0.13	0.34	0.44	0.00	0.00	0.19
BV510	0.00	0.00	5.16	2.79	0.97	0.71	0.83	0.82	0.00	0.97	0.44	1.38	0.00	2.22	1.84	1.02	0.77	0.78	0.67	0.00	0.51	0.24	0.00	0.18	0.44	1.18	0.75	0.59	0.53	0.00	0.00	0.00	0.00	0.42	0.24	0.27	0.00
BV570	0.00	0.00	0.00	2.20	0.80	0.51	0.46	0.46	1.03	1.04	0.47	0.49	0.00	0.00	2.04	1.37	1.06	0.54	0.78	0.00	0.80	0.63	0.00	0.00	0.37	2.56	3.52	0.79	1.05	0.85	0.57	0.41	0.00	0.47	0.19	0.00	0.42
BV605	0.00	0.00	0.00	1.08	2.69	1.14	1.02	1.06	0.63	0.96	0.31	0.26	0.00	1.11	0.00	1.63	1.41	1.15	1.09	0.51	0.84	1.07	0.81	0.63	0.32	4.74	5.35	1.81	1.60	1.48	0.88	0.77	0.60	0.64	0.37	0.02	0.30
BV650	0.00	0.33	0.00	0.00	0.64	2.34	1.28	1.22	0.84	1.01	0.45	0.00	0.90	0.33	0.92	0.00	1.89	1.50	1.47	0.00	0.66	1.11	0.77	0.55	0.44	0.79	0.67	0.42	1.78	1.47	0.75	0.67	1.51	1.24	0.75	0.45	0.43
BV711	0.00	0.00	0.00	0.00	0.12	0.73	2.75	2.49	1.03	1.31	0.44	0.31	0.50	0.00	0.23	0.64	0.00	2.73	3.04	0.00	0.36	0.57	0.78	0.87	0.69	0.48	0.00	0.00	0.46	1.08	0.85	0.91	1.42	1.77	4.59	1.22	0.81
BV750	0.14	0.00	0.00	0.00	0.24	0.19	3.75	3.05	0.91	1.02	0.00	0.00	0.00	0.00	0.00	0.59	1.12	0.00	4.03	0.00	0.34	0.28	0.37	0.91	0.87	0.00	0.00	0.24	0.27	0.32	0.51	0.66	0.41	0.47	0.94	1.59	0.77
BV785	0.33	0.00	0.00	0.00	0.00	0.11	1.69	4.38	0.90	0.82	0.33	0.00	0.00	0.06	0.05	0.40	0.59	1.99	0.00	0.35	0.38	0.23	0.00	0.43	0.54	0.32	0.00	0.00	0.00	0.00	0.32	0.52	0.30	0.29	0.50	1.90	0.87
Vio Bright B515	0.00	0.00	1.07	0.30	0.14	0.00	0.08	0.00	0.00	0.00	0.00	0.09	0.47	0.42	0.17	0.19	0.13	0.17	0.20	0.00	3.93	0.16	0.16	0.00	0.16	1.67	1.29	0.29	0.15	0.18	0.00	0.09	0.08	0.00	0.00	0.00	0.13
Vio Bright FITC	0.00	0.00	0.57	0.31	0.31	0.14	0.00	0.10	0.00	0.00	0.00	0.29	0.75	0.65	0.49	0.29	0.31	0.28	0.35	4.92	0.00	0.28	0.22	0.19	0.00	2.33	1.81	0.52	0.39	0.16	0.00	0.21	0.34	0.21	0.10	0.21	0.11
PerCP	1.19	0.00	0.00	0.00	0.00	1.87	2.90	2.30	1.04	0.00	0.00	0.00	0.00	0.82	1.48	2.68	2.79	1.67	1.56	2.03	3.11	0.00	1.98	2.01	1.85	0.00	0.00	0.00	1.62	2.89	1.72	1.67	2.01	0.00	1.19	0.60	0.00
PerCP-eFluor 710	0.15	0.00	0.00	0.00	0.00	1.05	2.13	2.20	0.00	0.00	0.00	0.00	0.00	0.00	0.07	0.98	4.32	2.60	2.59	0.00	0.53	1.51	0.00	2.95	3.81	1.59	0.00	0.31	0.99	4.53	3.42	2.54	2.02	2.27	5.85	1.64	1.11
RB744	0.10	0.00	0.00	0.00	0.24	0.30	2.46	1.98	0.48	0.00	0.00	0.00	0.40	0.00	0.26	0.44	1.53	1.87	1.39	0.60	0.98	0.19	1.98	0.00	6.96	0.61	0.42	0.14	0.11	1.25	0.72	1.06	0.31	0.30	1.19	1.50	0.60
RB780	0.13	0.00	0.62	0.31	0.27	0.13	1.07	2.29	0.18	0.33	0.00	0.00	0.00	0.51	0.39	0.67	0.99	0.57	1.77	1.05	1.43	0.26	1.10	3.20	0.00	0.97	0.57	0.14	0.33	0.86	0.68	0.98	0.48	0.23	0.42	1.28	0.52
PE	0.00	0.00	0.00	2.17	0.49	0.32	0.06	0.11	0.48	0.31	0.00	0.12	0.00	4.05	1.37	0.48	0.47	0.21	0.30	1.05	0.99	0.64	0.51	0.36	0.31	0.00	7.07	1.11	1.11	0.87	0.44	0.38	0.43	0.31	0.15	0.00	0.09
cFluor YG584	0.09	0.00	0.00	0.57	0.38	0.30	0.00	0.00	0.00	0.00	0.00	0.00	0.00	1.24	0.70	0.52	0.54	0.24	0.17	0.57	0.64	0.84	0.60	0.36	0.35	3.42	0.00	1.03	1.43	1.22	0.60	0.53	0.12	0.45	0.16	0.18	0.00
PE-Vio 615	0.00	0.00	0.00	0.57	3.47	0.84	0.64	0.61	0.23	0.00	0.00	0.00	0.32	0.76	2.24	1.03	2.15	0.59	0.55	0.43	0.63	2.15	2.13	1.30	1.11	5.10	10.4	0.00	3.43	3.58	1.63	1.37	1.61	1.28	0.64	0.35	0.26
PE-Cy5	0.00	0.00	0.48	0.52	2.37	1.87	0.81	0.69	0.09	0.00	0.00	0.20	0.00	0.21	0.83	1.92	6.97	1.16	0.75	0.10	0.36	3.86	4.48	1.78	1.85	2.98	3.53	1.38	0.00	9.98	3.88	2.14	4.04	2.86	1.79	0.73	0.66
PE-Cy5.5	0.00	0.00	0.00	0.34	0.50	2.02	1.05	0.87	0.00	0.00	0.00	0.00	0.21	0.54	0.37	0.93	3.89	1.13	0.84	0.30	0.58	2.52	5.77	2.26	2.40	4.62	3.75	0.86	2.02	0.00	5.62	2.76	2.20	3.48	3.37	0.74	0.66
cFluor BYG750	0.00	0.00	0.00	0.43	0.19	0.35	1.62	1.36	0.00	0.17	0.00	0.00	0.00	0.65	0.55	0.49	1.28	1.94	1.39	0.29	0.46	0.65	1.50	4.48	3.52	3.84	2.32	0.48	0.64	1.69	0.00	4.52	0.34	0.46	1.10	1.27	0.96
PE-Cy7	0.00	0.00	0.15	0.00	0.00	0.00	0.83	1.75	0.00	0.12	0.00	0.00	0.00	0.15	0.00	0.21	0.68	0.63	2.26	0.00	0.62	0.10	0.95	2.23	5.54	0.44	0.25	0.16	0.25	0.78	1.81	0.00	0.18	0.23	0.40	1.70	1.32
APC	0.00	0.00	0.00	0.07	0.15	1.06	0.98	0.91	0.00	0.00	0.00	0.00	0.00	0.00	0.76	3.16	1.17	0.93	0.91	0.48	0.00	1.64	0.93	0.68	0.68	0.40	0.64	0.14	2.69	1.89	1.17	1.12	0.00	3.05	1.53	1.38	0.79
eFluor 660	0.00	0.00	0.00	0.00	0.13	0.45	0.47	0.39	0.17	0.00	0.00	0.00	0.00	0.00	0.00	0.86	0.68	0.28	0.44	0.00	0.39	0.86	0.70	0.59	0.21	0.36	0.37	0.00	1.37	1.34	0.81	0.81	2.78	0.00	1.68	1.65	0.93
cFluor R720	0.00	0.00	0.00	0.00	0.20	0.45	1.31	1.38	0.33	0.34	0.00	0.00	0.00	0.00	0.38	0.60	2.68	1.12	1.04	0.00	0.52	0.48	0.45	0.58	0.60	0.37	0.00	0.20	0.54	0.78	0.79	1.15	1.30	1.66	0.00	2.71	2.20
APC-Vio 770	0.20	0.00	0.00	0.23	0.12	0.16	0.88	1.78	0.00	0.00	0.00	0.00	0.00	0.00	0.24	0.44	0.33	0.63	2.77	0.00	0.00	0.31	0.00	0.47	1.68	0.00	0.42	0.00	0.42	0.33	0.86	2.59	0.71	0.40	0.60	0.00	3.61
APC-Fire 810	0.00	0.00	0.59	0.00	0.27	0.30	0.46	2.44	0.43	0.39	0.00	0.22	0.00	0.00	0.20	0.72	0.59	1.57	2.94	0.00	0.55	0.38	0.24	0.30	0.96	0.26	0.00	0.00	0.50	0.72	0.54	1.86	1.09	0.93	0.52	2.16	0.00

0 6 12

Supplemental Figure 2. Fluorochrome pairs with high Similarity™ Indices and SSM values. Fluorochrome pairs with the highest Similarity™ Indices are depicted to visualize the impact on the distinction of each fluorochrome in (**A)** PBMCs and (**B)** HD liver gated on either the CD45-positive population or on total NK cells. Similarity™ Indices are indicated on top. **(C)** SSM generated with CD4-stained PBMCs. Spillover values are color-coded as follows: white <3, yellow 3-6, shades of red >6.

(A)

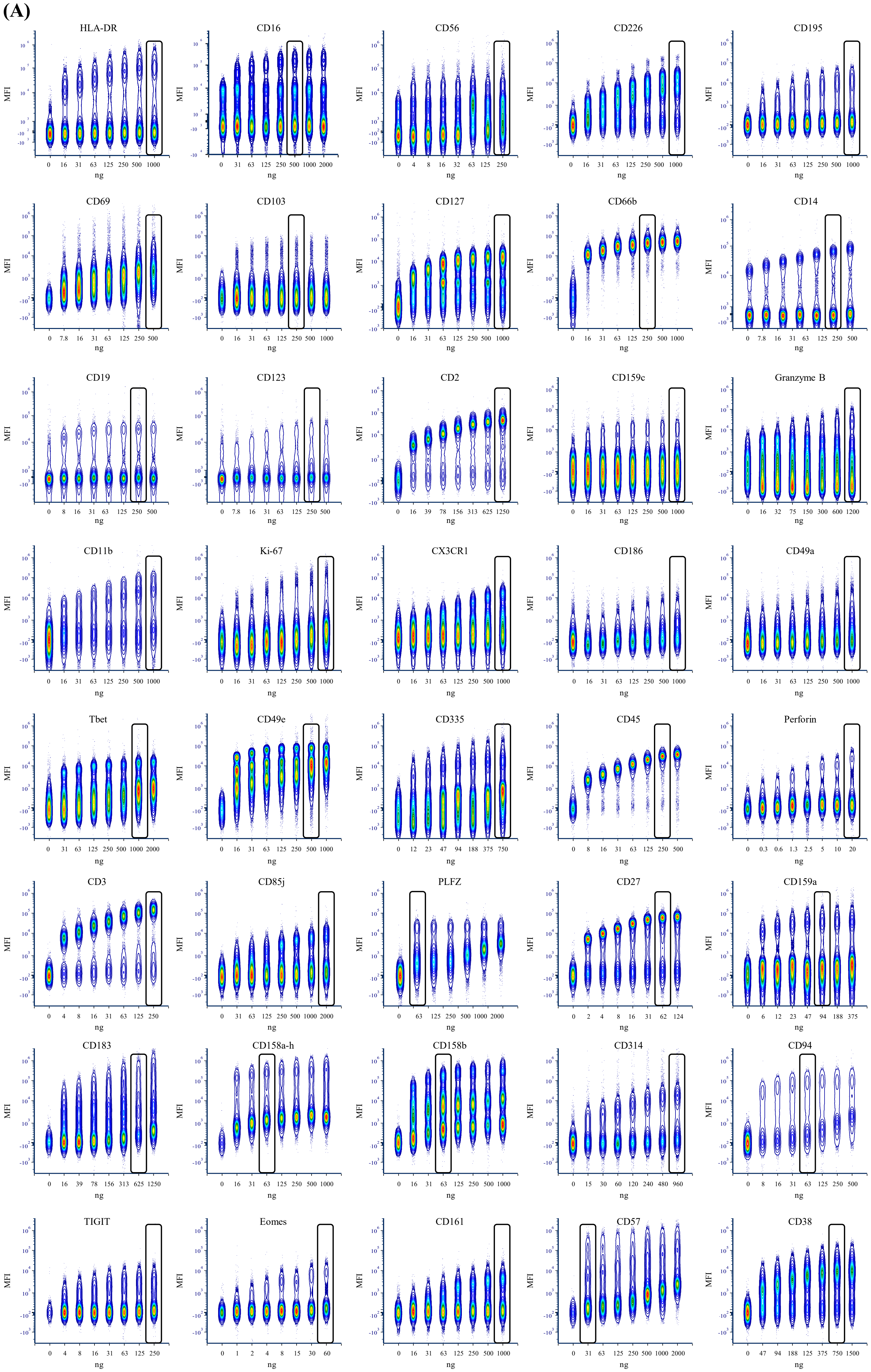
HLA-DR
0 16 31 63 125 250 500 1000
CD16
0 31 63 125 250 500 1000 2000
CD56
0 4 8 16 32 63 125 250
CD226
0 16 31 63 125 250 500 1000
CD195
0 16 31 63 125 250 500 1000
CD69
0 7.8 16 31 63 125 250 500
CD103
0 16 31 63 125 250 500 1000
CD127
0 16 31 63 125 250 500 1000
CD66b
0 16 31 63 125 250 500 1000
CD14
0 7.8 16 31 63 125 250 500
CD19
0 8 16 31 63 125 250 500
CD123
0 7.8 16 31 63 125 250 500
CD2
0 16 39 78 156 313 625 1250
CD159c
0 16 31 63 125 250 500 1000
Granzyme B
0 16 32 75 150 300 600 1200
CD11b
0 16 31 63 125 250 500 1000
Ki-67
0 16 31 63 125 250 500 1000
CX3CR1
0 16 31 63 125 250 500 1000
CD186
0 16 31 63 125 250 500 1000
CD49a
0 16 31 63 125 250 500 1000
Tbet
0 31 63 125 250 500 1000 2000
CD49e
0 16 31 63 125 250 500 1000
CD335
0 12 23 47 94 188 375 750
CD45
0 8 16 31 63 125 250 500
Perforin
0 0.3 0.6 1.3 2.5 5 10 20
CD3
0 4 8 16 31 63 125 250
CD85j
0 31 63 125 250 500 1000 2000
PLFZ
0 63 125 250 500 1000 2000
CD27
0 2 4 8 16 31 62 124
CD159a
0 6 12 23 47 94 188 375
CD183
0 16 39 78 156 313 625 1250
CD158a-h
0 16 31 63 125 250 500 1000
CD158b
0 16 31 63 125 250 500 1000
CD314
0 15 30 60 120 240 480 960
CD94
0 8 16 31 63 125 250 500
TIGIT
0 4 8 16 31 63 125 250
Eomes
0 1 2 4 8 15 30 60
CD161
0 16 31 63 125 250 500 1000
CD57
0 31 63 125 250 500 1000 2000
CD38
0 47 94 188 125 375 750 1500
MFI
ng

(B)

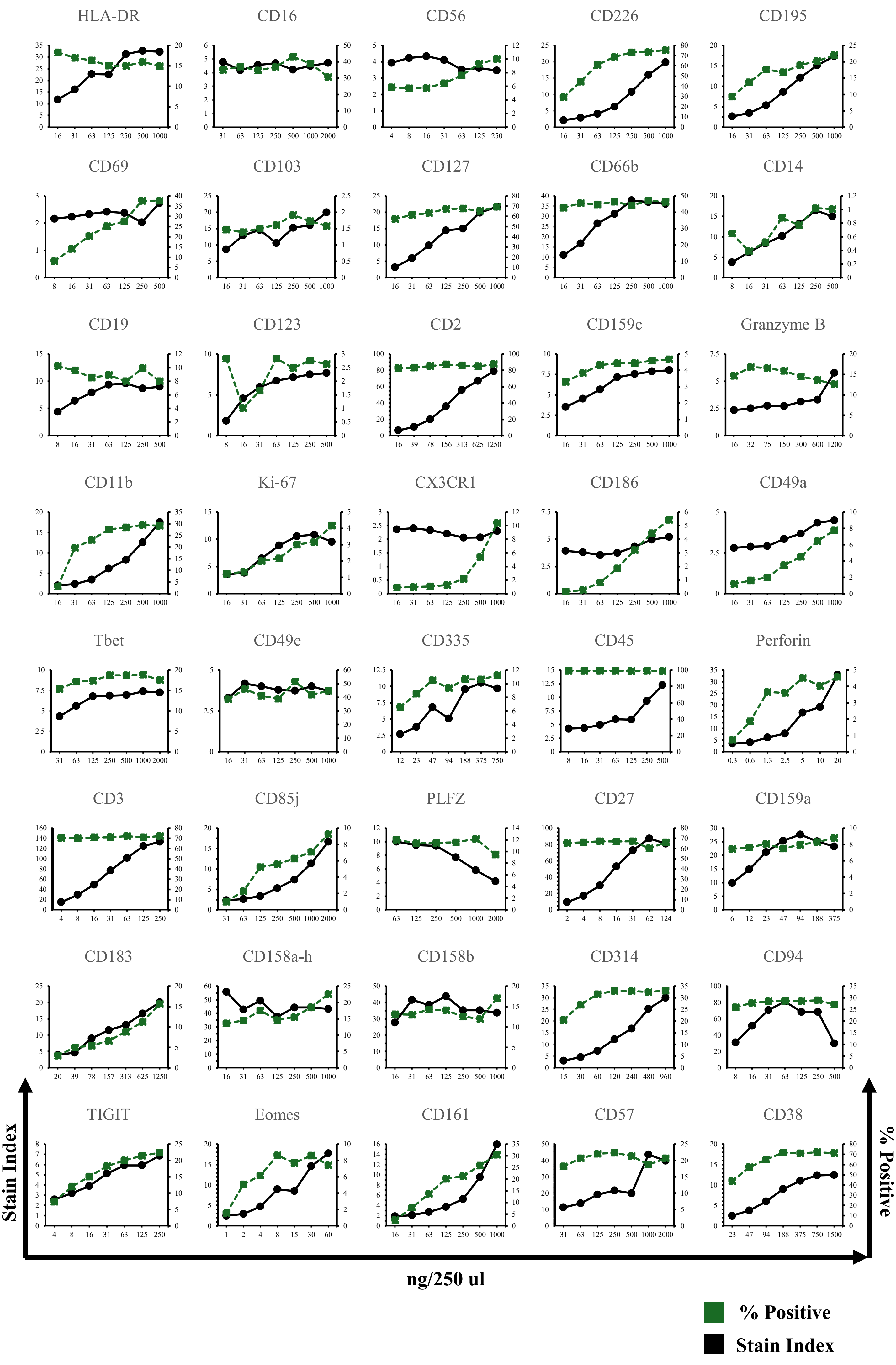

Supplemental Figure 3. Antibody titrations.
Two-fold dilutions of antibodies were tested in 7 serial dilutions, except for PLFZ (6 serial dilutions). Unmixed files were concatenated for analysis using FCS Express version 7 (De Novo Software). The titrations are depicted either as **(A)** concatenated files or as **(B)** graphs depicting the staining index and percentage positive with concentrations on the x-axis expressed as ng/250 µl. Selected titers of each reagent are listed in **Supplementary Table 1**.

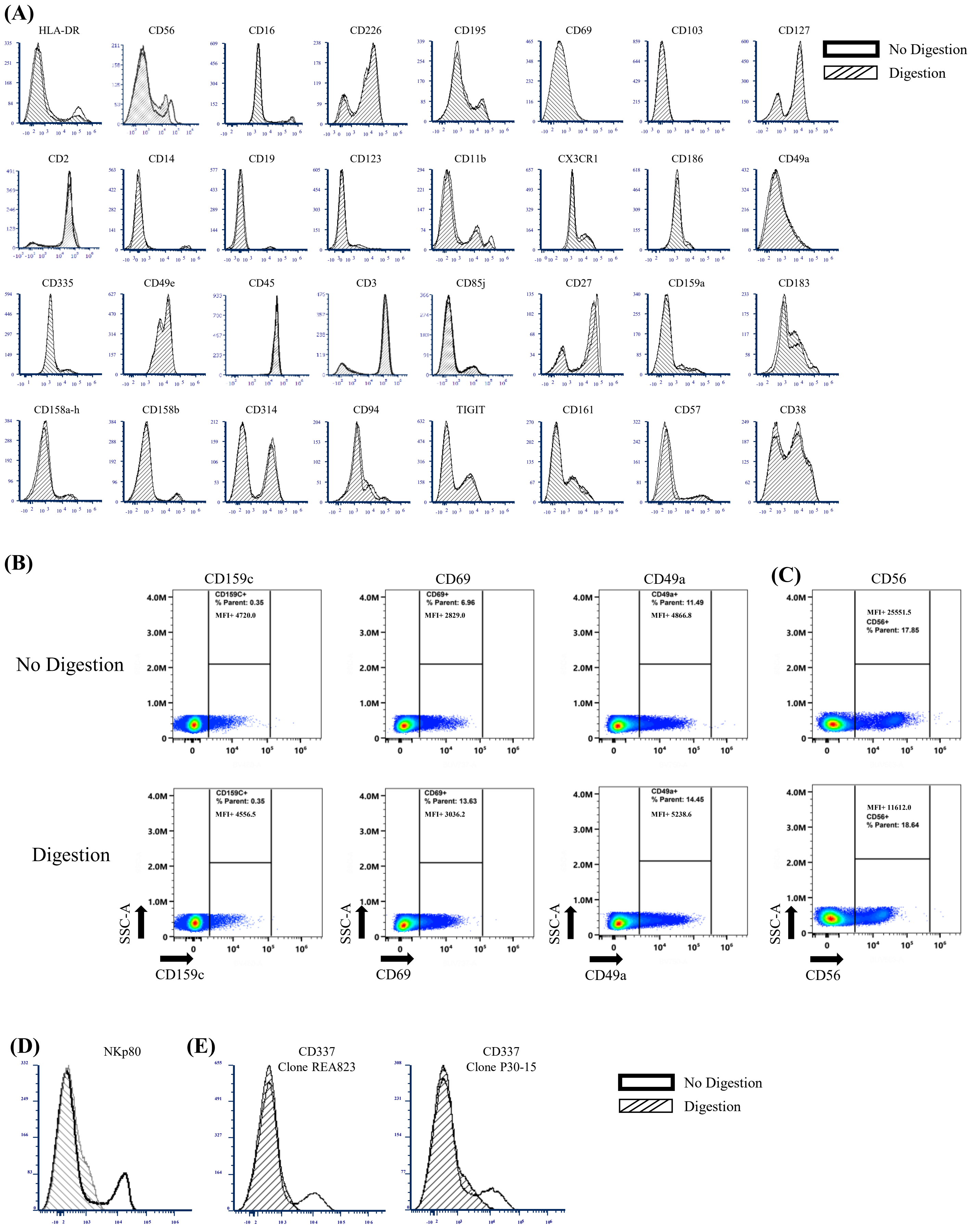

Supplemental Figure 4. Effect of digestion method on marker resolution.
The enzymatic digestion method used to isolate liver cells was evaluated for possible effects on the expression levels of surface markers. Therefore, PBMCs of the same donor were either non-treated or subjected to digestion and frozen until use. Subsequently, treated and non-treated PBMCs were stained with the optimal titer of each reagent, including a viability dye, and the staining patterns were compared by **(A)** histograms overlays or **(B)** pseudocolor plots for markers with a low frequency. **(C)** The reduction in CD56 staining intensity was still sufficient to identify all CD56-expressing leucocytes as percentage CD56-positives was equal between the two conditions. **(D)** and **(E)** depict markers excluded from the panel as digestion totally (**D**; NKp80) or dramatically reduced (**E**; CD337) the staining. All plots are gated on singlets/live and lymphocytes.

(A)

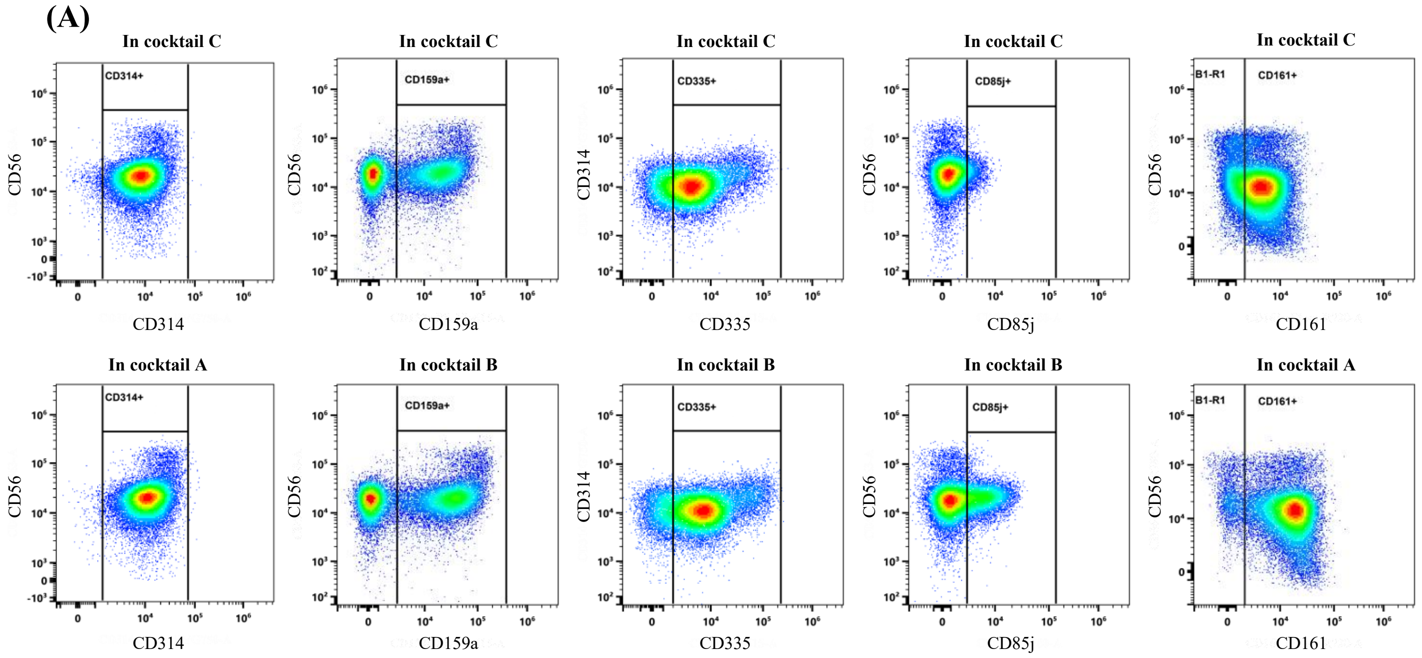

(B)

Marker	Order	Staining time (minutes)	% positive	MFI positive
CD314	Ab in cocktail C	65	97.98	7938.09
CD314	Ab in Cocktail A	65	99.15	10861.38
CD159a	Ab in Cocktail C	55	53.58	17475
CD159a	Ab in Cocktail A	55	55.65	28724.48
CD85j	Ab in Cocktail C	55	9.08	3954.26
CD85j	Ab in Cocktail A	55	33.98	6127.02
CD335	Ab in Cocktail C	55	76.69	5544.6
CD335	Ab in Cocktail A	55	81.52	7532.7

Supplemental Figure 5: Impact of sequential staining on antigen resolution.
(A) CD314, CD159a, CD335, CD85j and CD161 antibodies were either added to the master mix before staining (cocktail C; top lane) or added sequentially before cocktail C (either in cocktail A or B; bottom lane). Incubation time were adjusted so that the antibodies tested stained the sample for the same time regardless of the cocktail to which they were added. Incubation times, percentage and MFI of positive populations are shown in **(B)**.

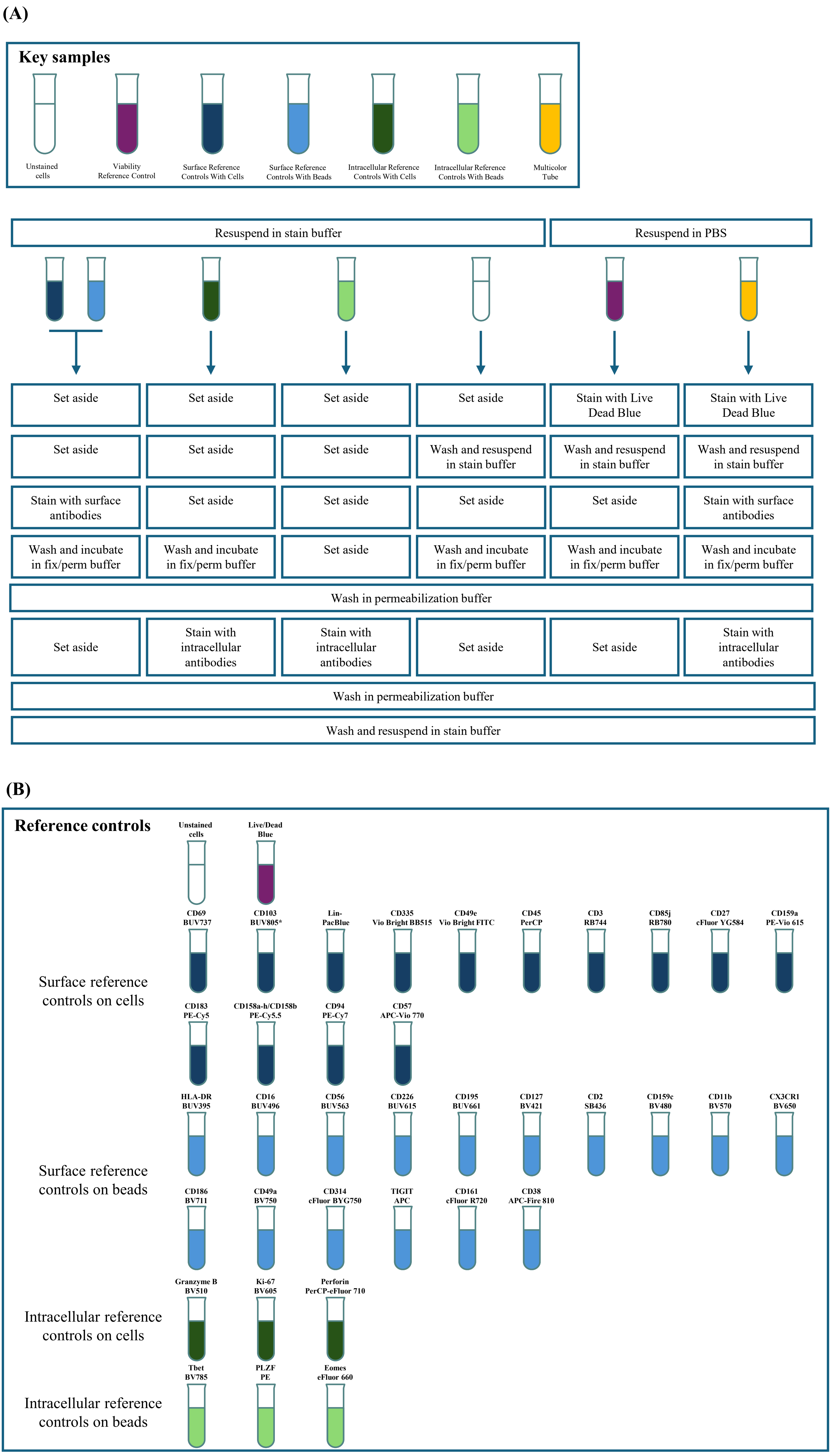
(A)
Key samples
Unstained cells
Viability Reference Control
Surface Reference Controls With Cells
Surface Reference Controls With Beads
Intracellular Reference Controls With Cells
Intracellular Reference Controls With Beads
Multicolor Tube
Resuspend in stain buffer
Resuspend in PBS
Set aside
Set aside
Set aside
Set aside
Stain with Live Dead Blue
Stain with Live Dead Blue
Set aside
Set aside
Set aside
Wash and resuspend in stain buffer
Wash and resuspend in stain buffer
Wash and resuspend in stain buffer
Stain with surface antibodies
Set aside
Set aside
Set aside
Set aside
Stain with surface antibodies
Wash and incubate in fix/perm buffer
Wash and incubate in fix/perm buffer
Set aside
Wash and incubate in fix/perm buffer
Wash and incubate in fix/perm buffer
Wash and incubate in fix/perm buffer
Wash in permeabilization buffer
Set aside
Stain with intracellular antibodies
Stain with intracellular antibodies
Set aside
Set aside
Stain with intracellular antibodies
Wash in permeabilization buffer
Wash and resuspend in stain buffer
(B)
Reference controls
Unstained cells
Live/Dead Blue
Surface reference controls on cells
CD69 BUV737
CD103 BUV805*
Lin- PacBlue
CD335 Vio Bright BB515
CD49e Vio Bright FITC
CD45 PerCP
CD3 RB744
CD85j RB780
CD27 cFluor YG584
CD159a PE-Vio 615
CD183 PE-Cy5
CD158a-h/CD158b PE-Cy5.5
CD94 PE-Cy7
CD57 APC-Vio 770
Surface reference controls on beads
HLA-DR BUV395
CD16 BUV496
CD56 BUV563
CD226 BUV615
CD195 BUV661
CD127 BV421
CD2 SB436
CD159c BV480
CD11b BV570
CX3CR1 BV650
CD186 BV711
CD49a BV750
CD314 cFluor BYG750
TIGIT APC
CD161 cFluor R720
CD38 APC-Fire 810
Intracellular reference controls on cells
Granzyme B BV510
Ki-67 BV605
Perforin PerCP-eFluor 710
Intracellular reference controls on beads
Tbet BV785
PLZF PE
Eomes eFluor 660

(C)

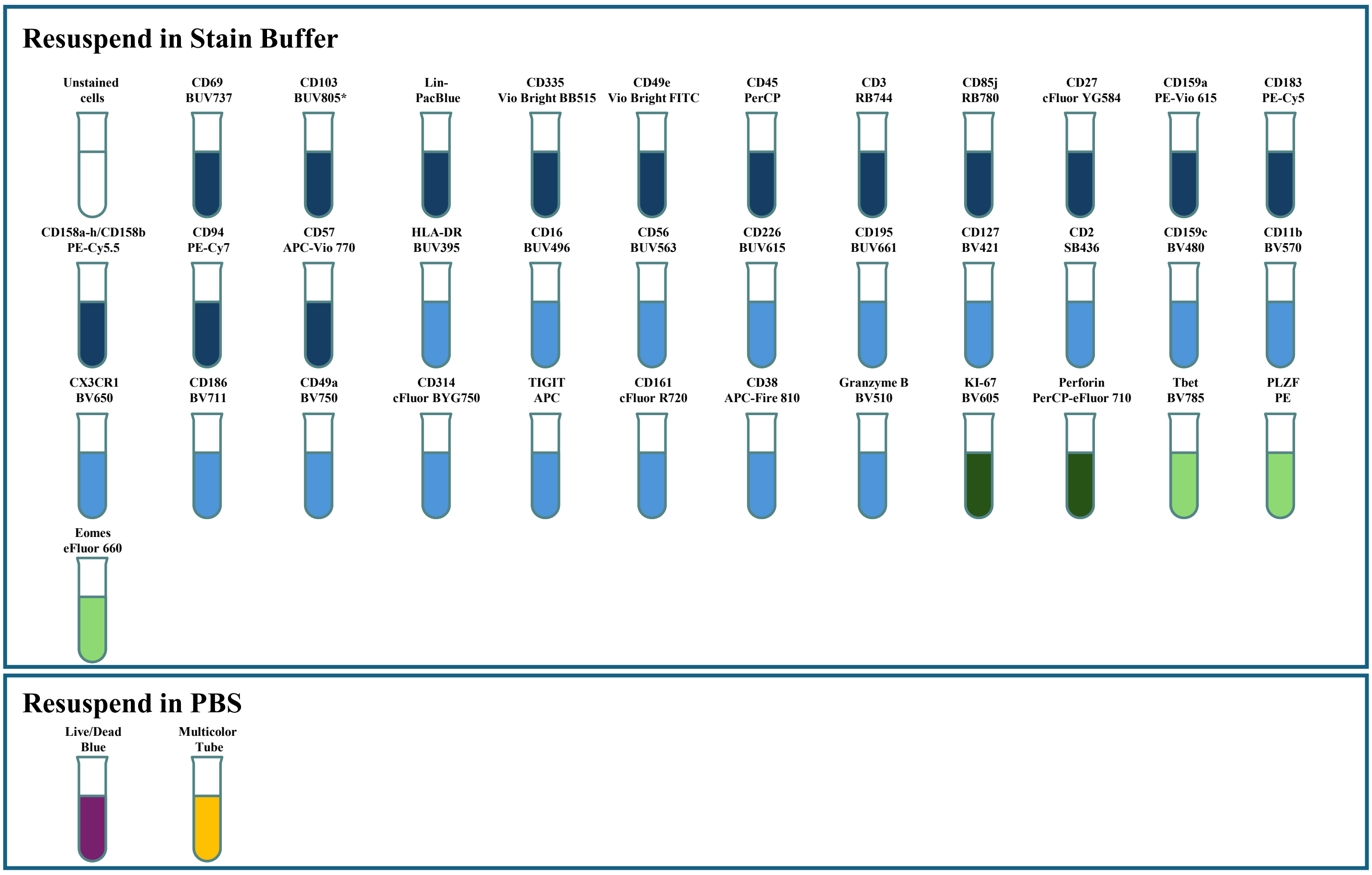
Resuspend in Stain Buffer
Unstained cells
CD69 BUV737
CD103 BUV805*
Lin- PacBlue
CD335 Vio Bright BB515
CD49e Vio Bright FITC
CD45 PerCP
CD3 RB744
CD85j RB780
CD27 cFluor YG584
CD159a PE-Vio 615
CD183 PE-Cy5
CD158a-h/CD158b PE-Cy5.5
CD94 PE-Cy7
CD57 APC-Vio 770
HLA-DR BUV395
CD16 BUV496
CD56 BUV563
CD226 BUV615
CD195 BUV661
CD127 BV421
CD2 SB436
CD159c BV480
CD11b BV570
CX3CR1 BV650
CD186 BV711
CD49a BV750
CD314 cFluor BYG750
TIGIT APC
CD161 cFluor R720
CD38 APC-Fire 810
Granzyme B BV510
KI-67 BV605
Perforin PerCP-eFluor 710
Tbet BV785
PLZF PE
Eomes eFluor 660
Resuspend in PBS
Live/Dead Blue
Multicolor Tube

(D)

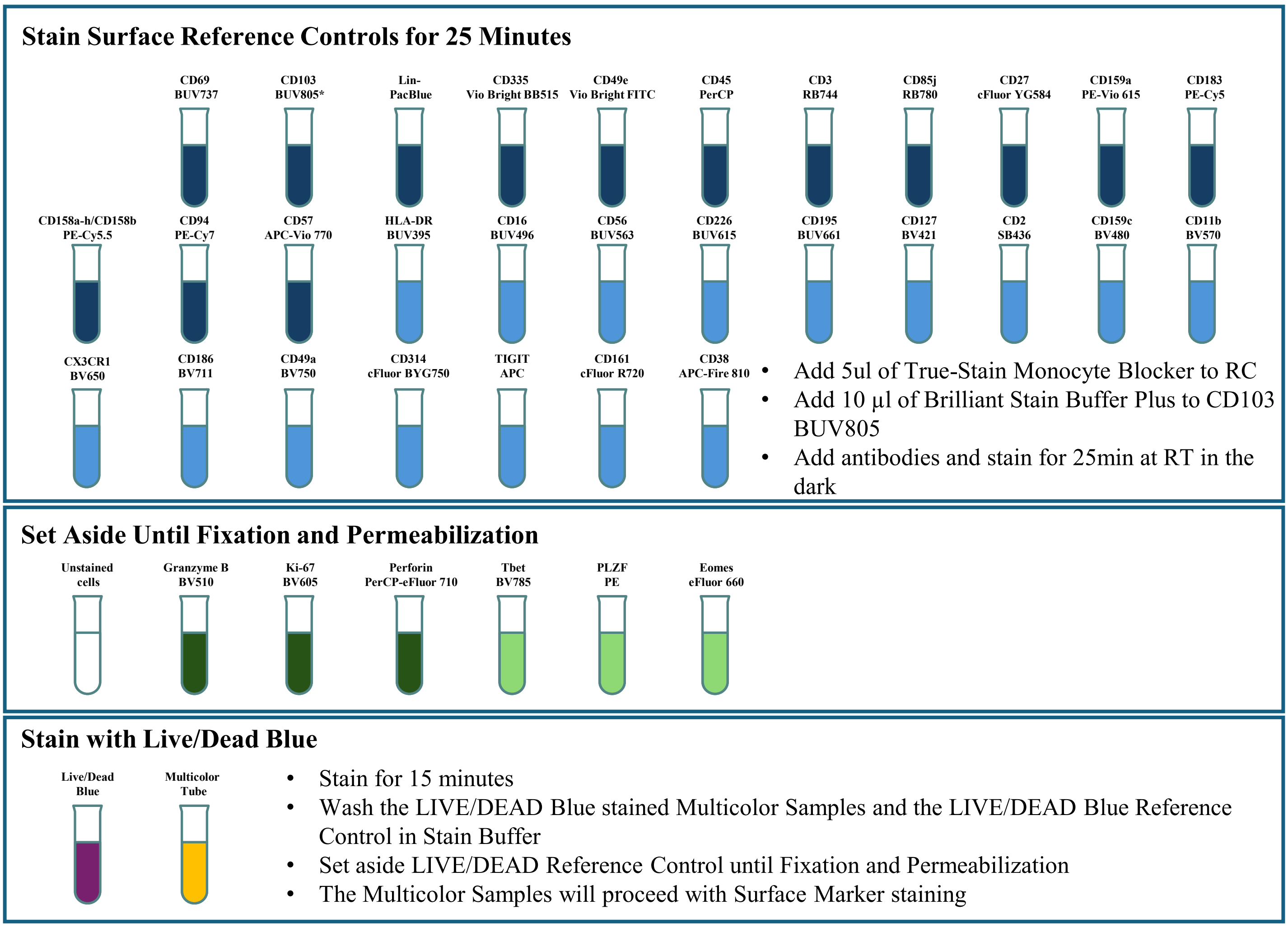
Stain Surface Reference Controls for 25 Minutes
CD69 BUV737
CD103 BUV805*
Lin- PacBlue
CD335 Vio Bright BB515
CD49e Vio Bright FITC
CD45 PerCP
CD3 RB744
CD85j RB780
CD27 cFluor YG584
CD159a PE-Vio 615
CD183 PE-Cy5
CD158a-h/CD158b PE-Cy5.5
CD94 PE-Cy7
CD57 APC-Vio 770
HLA-DR BUV395
CD16 BUV496
CD56 BUV563
CD226 BUV615
CD195 BUV661
CD127 BV421
CD2 SB436
CD159c BV480
CD11b BV570
CX3CR1 BV650
CD186 BV711
CD49a BV750
CD314 cFluor BYG750
TIGIT APC
CD161 cFluor R720
CD38 APC-Fire 810
• Add 5ul of True-Stain Monocyte Blocker to RC
• Add 10 µl of Brilliant Stain Buffer Plus to CD103 BUV805
• Add antibodies and stain for 25min at RT in the dark
Set Aside Until Fixation and Permeabilization
Unstained cells
Granzyme B BV510
Ki-67 BV605
Perforin PerCP-eFluor 710
Tbet BV785
PLZF PE
Eomes eFluor 660
Stain with Live/Dead Blue
Live/Dead Blue
Multicolor Tube
• Stain for 15 minutes
• Wash the LIVE/DEAD Blue stained Multicolor Samples and the LIVE/DEAD Blue Reference Control in Stain Buffer
• Set aside LIVE/DEAD Reference Control until Fixation and Permeabilization
• The Multicolor Samples will proceed with Surface Marker staining

(E)

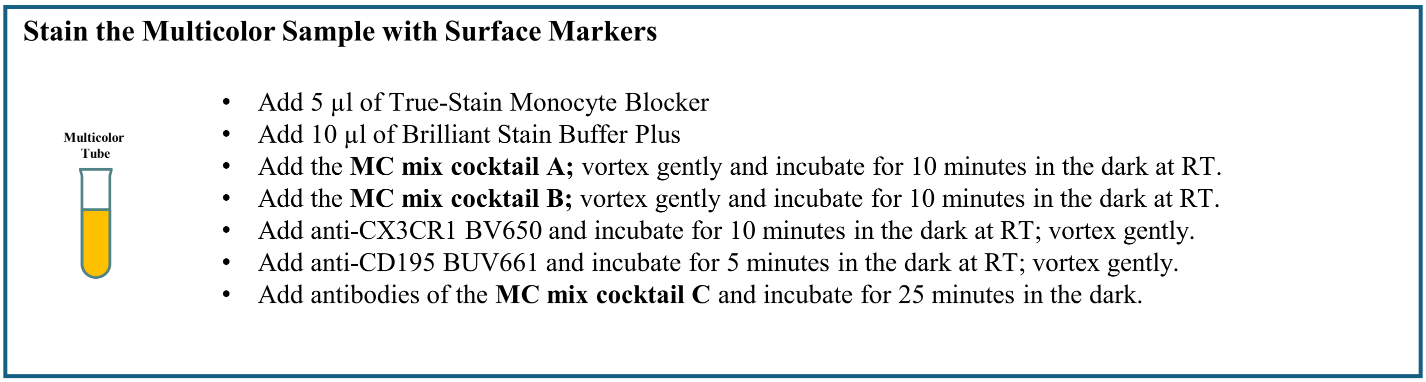
Stain the Multicolor Sample with Surface Markers
Multicolor Tube
• Add 5 µl of True-Stain Monocyte Blocker
• Add 10 µl of Brilliant Stain Buffer Plus
• Add the MC mix cocktail A; vortex gently and incubate for 10 minutes in the dark at RT.
• Add the MC mix cocktail B; vortex gently and incubate for 10 minutes in the dark at RT.
• Add anti-CX3CR1 BV650 and incubate for 10 minutes in the dark at RT; vortex gently.
• Add anti-CD195 BUV661 and incubate for 5 minutes in the dark at RT; vortex gently.
• Add antibodies of the MC mix cocktail C and incubate for 25 minutes in the dark.

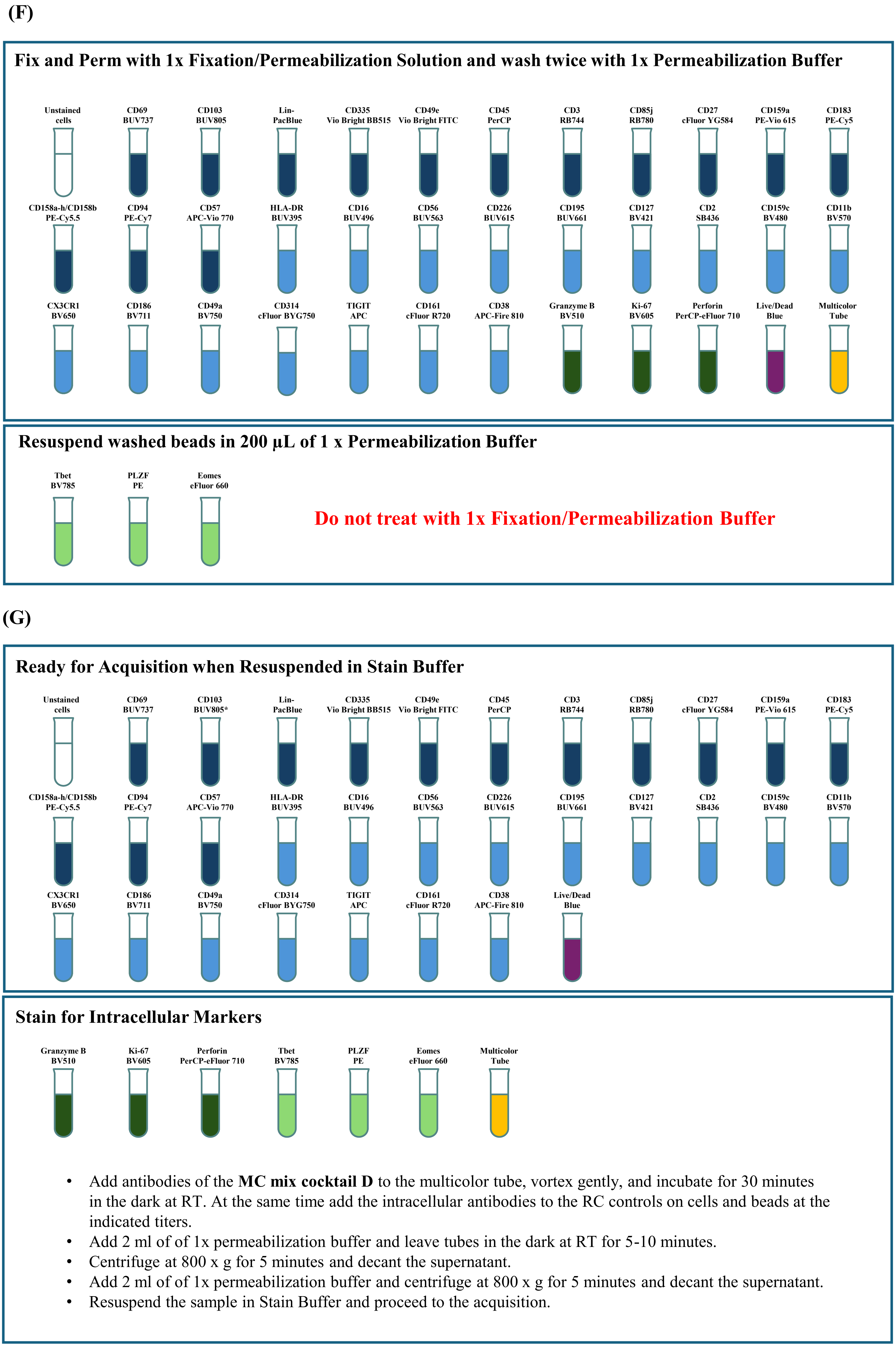

Supplemental Figure 6, Schematic overview of the staining protocol and recommended RCs
Scheme that depicts **(A)** complete staining workflow and key samples to include, **(B)** detail of reference controls, **(C)** different washing steps to follow, **(D)** surface staining step and steps that can be done in parallel, **(E)** staining of the MC tube and additional reagents to add before staining with antibodies, **(F)** fixation and permeabilization steps for cells versus beads and **(G)** ICC staining protocol and washing steps. The acquisition of RCs for surface markers can be done before or after ICC staining and after the fixation and permeabilization has been completed as depicted in **(H)**.

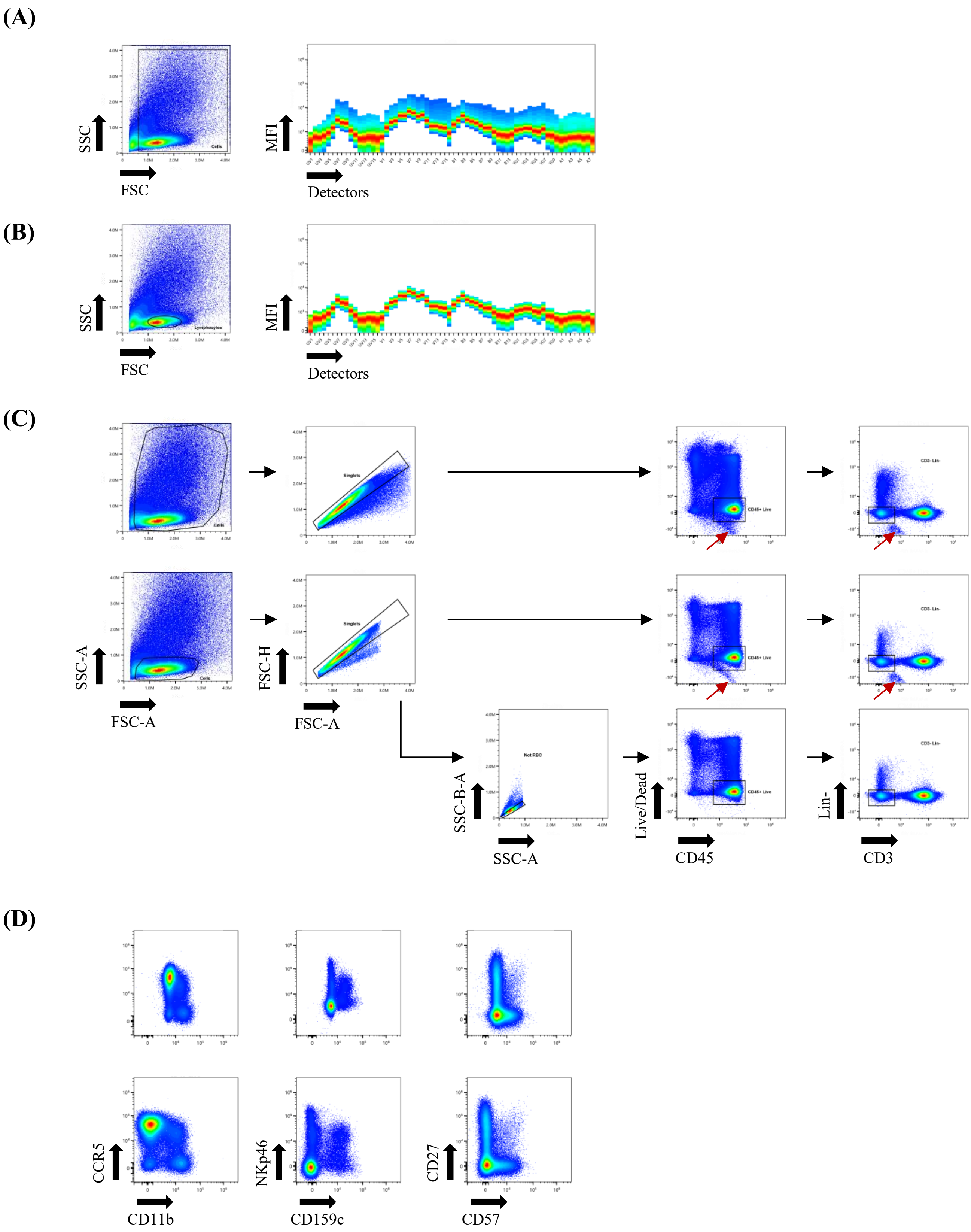

Supplemental Figure 7. (A) Spectral signature of the AF detected in healthy liver when the gate is set widely on a FSC-SSC dot plot. **(B)** Spectral signature of the AF detected in healthy liver when the gate is set tight on lymphoid cells. **(C)** Importance of the gating strategy. Top lane: Gating strategy including a wide gate on FSC-SSC dot plot and no red blood cell (RBC) exclusion. Middle lane: Gating strategy including a thigh gate on lymphoid cells and no exclusion of RBC. Bottom lane: Gating strategy including a thigh gate on lymphoid cells and exclusion of RBC by using SSC-A versus SSC-B-A. Artifactual populations are indicated by a red arrow. **(D)** Examples of markers that benefited from AF extraction by improving resolution. Top lane depicts a MC sample without AF extraction and lower lane with AF extraction.

(A)

	BUV395	LIVE DEAD Blue	BUV496	BUV563	BUV615	BUV661	BUV737	BUV805	BV421	Super Bright 436	cFluor V450	BV480	BV510	BV570	BV605	BV650	BV711	BV750	BV785	Vio Bright B515	Vio Bright FITC	PerCP	PerCP-eFluor 710	RB744	RB780	PE	cFluor YG584	PE-Vio 615	PE-Cy5	PE-Cy5.5	cFluor BYG750	PE-Cy7	APC	eFluor 660	cFluor R720	APC-Vio 770	APC-Fire 810
BUV395	0.00	6.82	2.67	0.71	0.12	0.19	0.19	0.30	0.80	1.16	1.40	0.52	2.66	0.69	0.46	0.25	0.00	0.00	0.44	0.23	1.94	0.00	0.45	0.29	0.00	1.55	0.62	0.63	0.18	0.03	0.00	0.18	0.28	0.36	0.19	0.17	0.00
LIVE DEAD Blue	1.76	0.00	5.53	3.29	0.50	1.24	1.20	0.00	0.00	0.00	2.41	1.40	6.43	0.29	0.00	0.79	0.00	0.00	1.02	2.54	2.77	0.00	1.85	0.00	0.00	2.14	0.00	1.87	1.22	0.99	1.05	0.00	0.54	1.37	0.00	0.00	0.00
BUV496	0.63	0.76	0.00	1.96	0.83	0.47	0.32	0.34	0.18	0.66	0.76	1.23	1.71	1.20	0.41	0.00	0.00	0.00	0.00	1.24	0.00	0.00	0.10	0.18	0.18	0.95	0.30	0.26	0.21	0.16	0.15	0.10	0.16	0.15	0.12	0.00	0.00
BUV563	0.57	0.62	0.00	0.00	1.80	1.02	1.00	0.71	0.86	0.85	0.97	1.21	1.79	1.21	0.62	0.00	0.00	0.00	0.00	1.82	1.86	0.77	0.20	0.50	0.22	4.18	4.71	0.98	0.95	0.95	0.55	0.60	0.23	0.54	0.00	0.00	0.22
BUV615	0.80	0.88	6.17	2.95	0.00	2.44	2.25	2.10	0.00	0.00	0.00	0.00	0.00	0.89	1.73	0.91	0.00	1.00	1.01	1.15	0.00	1.61	1.10	1.08	0.00	0.32	2.13	1.67	2.25	2.10	1.36	1.31	1.21	1.07	0.79	0.06	0.70
BUV661	1.11	1.36	4.58	2.03	0.78	0.00	3.56	3.51	1.75	4.90	2.31	0.23	6.80	1.44	1.11	2.37	0.98	1.17	0.00	1.77	4.96	1.83	1.28	1.12	0.08	4.77	2.09	2.21	2.61	2.46	1.12	0.96	4.18	3.05	1.62	1.50	0.73
BUV737	0.92	1.25	4.14	0.91	0.00	0.99	0.00	7.07	0.86	1.27	2.64	1.46	5.57	1.02	1.05	0.60	1.81	2.36	1.28	1.05	3.40	0.72	1.51	2.24	1.72	2.31	1.50	1.11	1.06	1.48	0.80	1.07	1.33	1.47	3.25	1.83	1.63
BUV805	0.90	0.69	0.00	0.00	0.00	0.38	2.03	0.00	0.28	0.00	0.00	0.00	0.00	0.00	0.00	0.00	0.00	1.12	1.93	0.00	0.00	0.45	0.00	0.70	0.19	0.00	0.00	0.00	0.06	0.00	0.03	0.72	0.00	0.41	0.33	1.49	1.87
BV421	0.00	0.74	0.00	0.00	0.93	0.70	0.00	0.00	0.00	5.06	3.28	0.00	0.00	0.54	0.95	1.47	0.99	0.56	1.48	0.75	2.03	0.00	0.50	0.00	0.00	0.00	1.37	0.00	0.98	0.00	0.61	0.00	0.00	0.00	0.00	0.60	0.00
Super Bright 436	0.18	1.28	1.47	0.69	0.26	0.00	0.03	0.26	2.97	0.00	2.76	1.42	1.33	0.95	0.50	0.71	0.59	0.21	0.79	0.00	0.88	0.00	0.00	0.00	0.48	0.00	0.00	0.05	0.35	0.00	0.19	0.00	0.00	0.21	0.00	0.00	0.00
cFluor V450	0.97	1.75	0.00	0.00	0.08	0.66	0.00	0.54	3.13	7.53	0.00	2.78	7.38	0.81	1.26	0.57	0.39	0.44	0.46	1.46	5.13	0.45	0.98	0.00	0.43	4.16	2.51	1.73	0.40	0.51	0.00	0.40	0.00	0.26	0.00	0.68	0.00
BV480	0.67	0.02	0.00	0.00	0.98	0.00	0.47	0.00	1.78	3.23	1.83	0.00	4.51	2.32	1.25	0.00	0.08	0.10	0.00	2.20	3.05	0.00	0.00	0.55	0.06	2.23	1.56	1.03	0.00	0.00	0.00	0.78	1.05	0.00	0.00	0.75	1.10
BV510	0.31	0.72	9.14	4.09	1.15	0.83	0.79	0.84	0.66	1.04	0.95	1.94	0.00	2.60	1.81	0.85	0.57	0.70	0.54	0.50	0.00	0.27	0.00	0.00	0.00	1.61	0.65	0.48	0.38	0.21	0.18	0.19	0.51	0.00	0.23	0.22	0.00
BV570	0.64	0.59	0.72	3.13	1.07	1.02	0.72	0.79	1.14	1.73	1.76	1.03	2.86	0.00	2.30	1.24	0.47	0.00	0.71	0.76	1.90	0.84	1.10	0.62	0.29	3.78	3.96	1.42	1.27	1.38	0.47	0.99	0.98	0.50	0.00	0.46	0.00
BV605	0.80	1.30	0.00	0.86	2.30	1.47	1.43	1.44	0.87	1.80	2.09	1.09	5.21	1.16	0.00	1.98	2.07	1.53	0.77	0.81	2.69	1.42	1.02	0.74	0.00	4.46	5.79	2.13	2.04	2.07	1.18	0.66	0.94	0.80	0.34	0.08	0.00
BV650	1.01	1.22	4.41	1.23	1.16	2.74	1.72	1.68	2.86	6.14	2.65	1.65	7.01	1.49	2.05	0.00	2.08	2.03	1.50	1.95	4.92	1.56	0.94	0.38	1.26	4.93	3.52	2.42	2.47	1.51	1.22	1.19	2.31	1.68	0.95	1.33	0.77
BV711	1.42	1.34	0.00	0.00	0.57	1.46	2.95	2.66	1.53	4.74	4.58	2.09	6.06	1.34	2.03	1.15	0.00	3.31	3.45	1.98	5.36	0.96	2.97	1.49	1.81	5.36	0.00	1.81	0.88	2.23	1.07	1.26	0.00	1.90	2.38	1.05	1.54
BV750	0.70	1.24	2.85	1.19	0.00	0.71	3.69	3.51	1.14	0.78	1.17	0.86	0.00	1.33	1.25	1.34	1.89	0.00	4.30	0.00	3.05	0.63	1.15	1.36	1.18	2.34	1.76	0.53	0.70	0.63	0.93	1.24	0.78	0.73	1.24	1.93	0.82
BV785	0.37	0.90	4.68	1.63	0.39	0.26	1.72	3.65	0.98	0.99	0.00	0.89	0.00	0.00	0.00	0.00	0.00	1.95	0.00	0.61	0.00	0.27	0.00	0.42	0.54	0.00	0.00	0.00	0.00	0.33	0.42	0.68	0.33	0.71	0.52	1.35	0.86
Vio Bright B515	0.26	0.00	0.00	0.00	0.00	0.65	0.00	0.00	1.40	0.00	0.00	0.55	0.00	0.40	0.00	0.00	0.00	0.84	0.00	0.00	2.72	0.27	0.00	0.00	0.33	0.00	0.00	0.00	0.70	0.00	0.00	0.30	0.00	0.00	0.26	0.51	0.00
Vio Bright FITC	0.00	0.04	2.45	1.05	0.00	0.00	0.05	0.32	0.41	0.66	0.00	0.00	0.00	0.54	0.00	0.00	0.00	0.00	0.00	4.65	0.00	0.31	0.36	0.00	0.07	2.07	1.40	0.35	0.00	0.53	0.00	0.00	0.00	0.54	0.30	0.05	0.00
PerCP	0.00	0.00	0.00	0.00	0.61	1.96	2.93	2.25	0.00	0.00	0.00	0.00	0.00	0.00	0.00	1.51	2.05	1.42	1.30	0.00	0.38	0.00	2.33	1.83	1.42	0.00	0.10	0.67	2.28	2.88	1.86	1.38	1.43	1.77	1.21	0.54	0.50
PerCP-eFluor 710	0.00	0.00	0.00	0.00	0.00	1.01	2.49	2.45	0.62	0.39	0.00	0.89	0.00	0.00	0.00	0.00	4.09	2.46	2.26	0.42	0.00	1.53	0.00	2.67	3.20	0.00	0.00	0.00	1.00	5.13	2.53	2.30	1.51	1.71	5.25	1.86	1.30
RB744	0.13	0.21	1.11	0.40	0.16	0.41	2.94	2.07	0.28	0.28	0.00	0.00	0.00	0.06	0.19	0.33	1.77	2.26	1.37	0.65	0.93	0.29	2.23	0.00	7.62	0.51	0.43	0.00	0.45	1.47	0.86	1.22	0.49	0.35	1.47	2.11	0.66
RB780	0.50	0.77	3.70	1.50	0.23	0.39	0.99	2.41	0.00	1.12	1.18	1.18	3.40	0.58	0.60	0.00	0.79	0.84	1.64	1.74	2.37	0.33	1.03	2.58	0.00	2.43	1.44	0.97	0.54	0.87	0.57	0.98	0.50	0.55	0.44	1.10	0.69
PE	0.00	0.00	0.00	0.00	0.00	0.43	0.43	0.67	0.74	0.99	0.00	0.92	0.00	1.85	0.00	0.00	0.00	0.68	0.00	0.83	0.00	0.55	0.35	0.49	0.00	0.00	3.92	0.74	1.11	0.97	0.36	0.51	0.55	0.00	0.00	0.00	0.00
cFluor YG584	0.00	0.00	0.00	0.00	0.17	0.24	0.00	0.18	0.00	0.00	0.00	0.00	0.00	1.37	0.92	0.95	0.88	0.60	0.62	0.00	1.92	0.88	0.79	0.27	0.19	3.38	0.00	0.99	1.44	1.33	0.55	0.56	0.00	0.00	0.29	0.37	0.00
PE-Vio 615	0.43	0.00	0.00	0.00	2.89	1.03	0.68	0.43	0.92	0.35	0.00	0.51	0.00	0.92	2.28	0.90	2.25	0.78	0.00	0.93	0.00	2.59	2.89	1.46	1.24	5.04	7.99	0.00	3.81	4.04	1.69	1.43	1.71	1.39	0.78	0.28	0.46
PE-Cy5	0.30	0.49	1.59	0.83	1.20	1.30	0.96	0.77	0.00	0.33	0.74	0.66	0.00	0.34	0.70	1.87	4.54	1.06	0.96	0.52	0.00	3.91	4.31	1.90	1.64	3.80	2.09	1.24	0.00	6.99	2.48	1.92	2.94	2.68	1.40	0.84	0.70
PE-Cy5.5	0.00	0.00	0.00	0.26	0.51	1.36	1.06	0.89	0.00	1.00	0.00	0.57	0.91	0.00	0.00	0.50	4.42	1.06	0.83	0.00	0.00	2.71	6.90	2.31	2.14	1.78	1.49	0.81	1.60	0.00	3.41	2.50	1.75	2.35	3.86	1.05	0.53
cFluor BYG750	0.04	0.70	4.59	1.42	0.43	0.75	1.66	1.26	0.55	0.52	0.00	0.00	0.00	0.00	0.00	0.00	0.65	1.87	1.14	0.38	0.00	0.29	1.30	4.60	3.18	0.00	0.00	0.00	0.33	1.84	0.00	3.81	0.00	0.00	0.64	1.34	0.98
PE-Cy7	0.00	0.00	0.50	0.00	0.12	0.00	0.99	1.85	0.44	0.00	0.00	0.31	0.00	0.00	0.00	0.00	0.76	0.85	2.46	0.00	0.00	0.12	1.10	2.41	6.28	0.00	0.00	0.00	0.00	0.99	1.94	0.00	0.16	0.00	0.48	2.05	1.28
APC	0.00	0.00	3.26	0.00	0.42	1.13	1.18	0.78	0.00	0.00	0.00	0.68	0.00	0.00	0.00	1.31	0.00	0.79	0.00	0.00	0.00	1.52	0.47	0.94	0.68	0.00	0.00	0.00	2.57	2.30	1.08	1.63	0.00	3.06	1.26	1.32	1.18
eFluor 660	0.00	0.00	6.23	1.63	0.00	0.00	0.00	0.00	0.62	0.00	0.00	0.72	0.00	0.00	0.00	0.00	0.00	0.00	0.00	0.00	0.00	0.65	0.00	0.71	0.00	0.00	0.00	0.00	1.31	1.13	1.01	1.03	2.62	0.00	1.49	1.58	1.26
cFluor R720	0.00	0.00	2.82	1.23	0.00	0.88	1.27	1.30	0.00	0.00	0.00	1.45	0.00	0.00	0.00	0.00	1.71	1.14	0.00	1.12	0.00	0.07	0.44	0.00	0.13	0.00	0.00	0.41	0.92	1.14	0.90	1.46	2.00	1.54	0.00	2.35	1.74
APC-Vio 770	0.20	0.29	2.52	0.81	0.15	0.35	0.94	2.04	0.53	0.54	0.14	0.43	0.00	0.00	0.13	0.49	0.18	0.63	5.17	0.12	0.00	0.36	0.29	0.54	1.30	0.00	0.00	0.00	0.51	0.58	1.05	3.87	1.61	0.68	0.67	0.00	4.66
APC-Fire 810	0.33	0.00	0.00	0.00	0.00	0.35	0.56	2.69	0.00	0.00	0.00	0.89	0.00	0.00	0.00	0.13	0.52	1.73	3.52	0.65	0.00	0.28	0.00	0.32	0.90	0.78	0.00	0.35	0.63	0.36	0.64	1.91	1.18	1.02	0.69	1.96	0.00

0 6 12

(B)

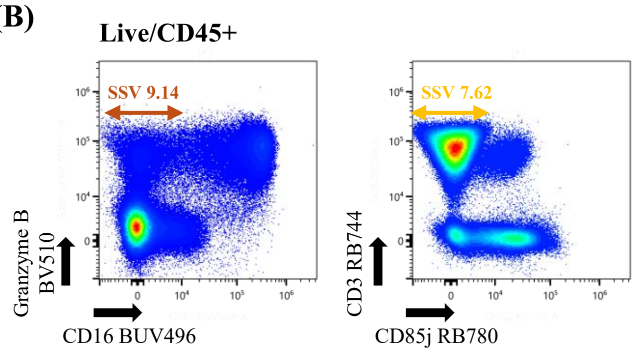

(C)

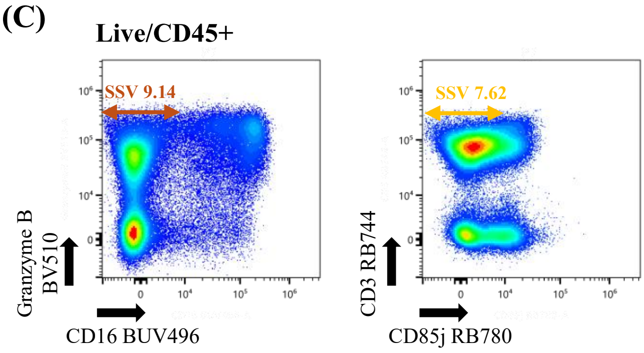

Supplemental Figure 8. **Antigen specific SSM and impact of high SSV on data resolution.**
SSM calculated using **(A)** antigen-specific stained PBMCs. (**B**) Visualization of the impact of high SSVs on data resolution in PBMCs and **(C)** in HD liver. Spillover values are color-coded as follows: white <3, yellow 3-6, shades of red >6.

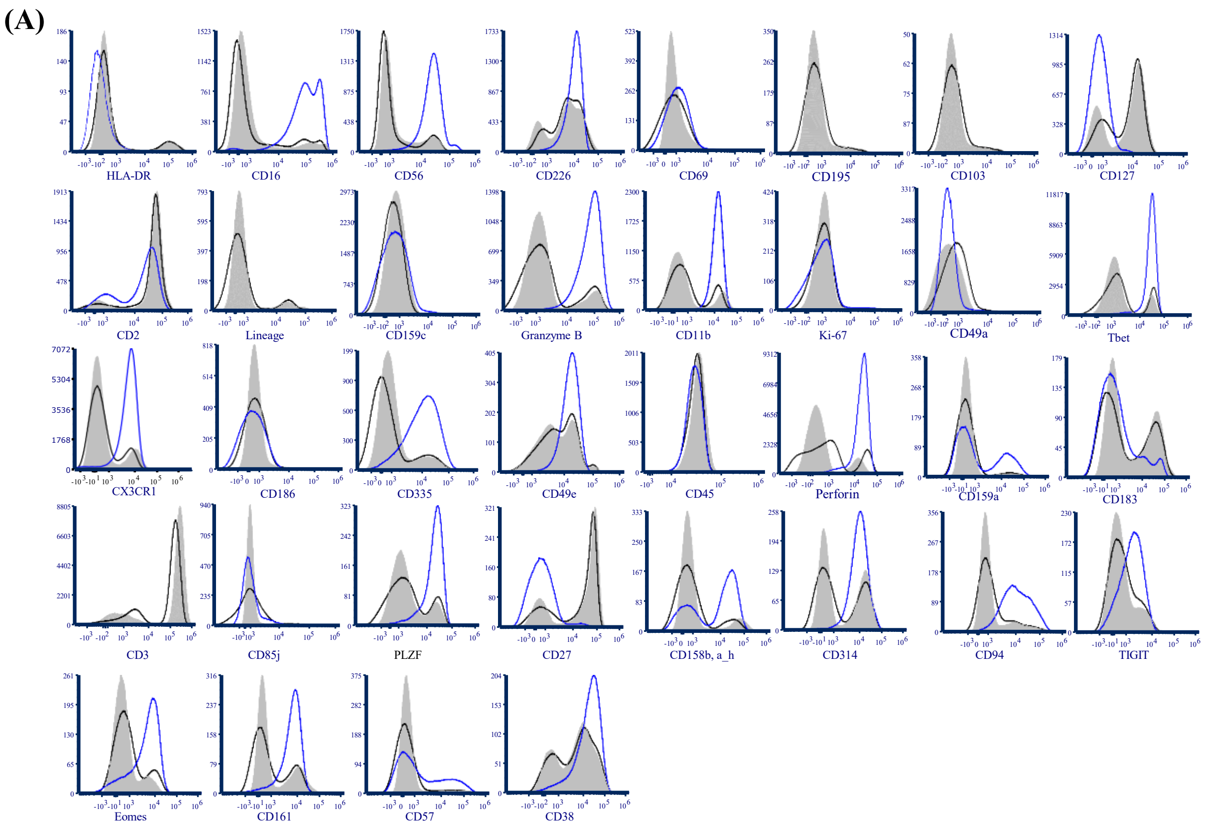

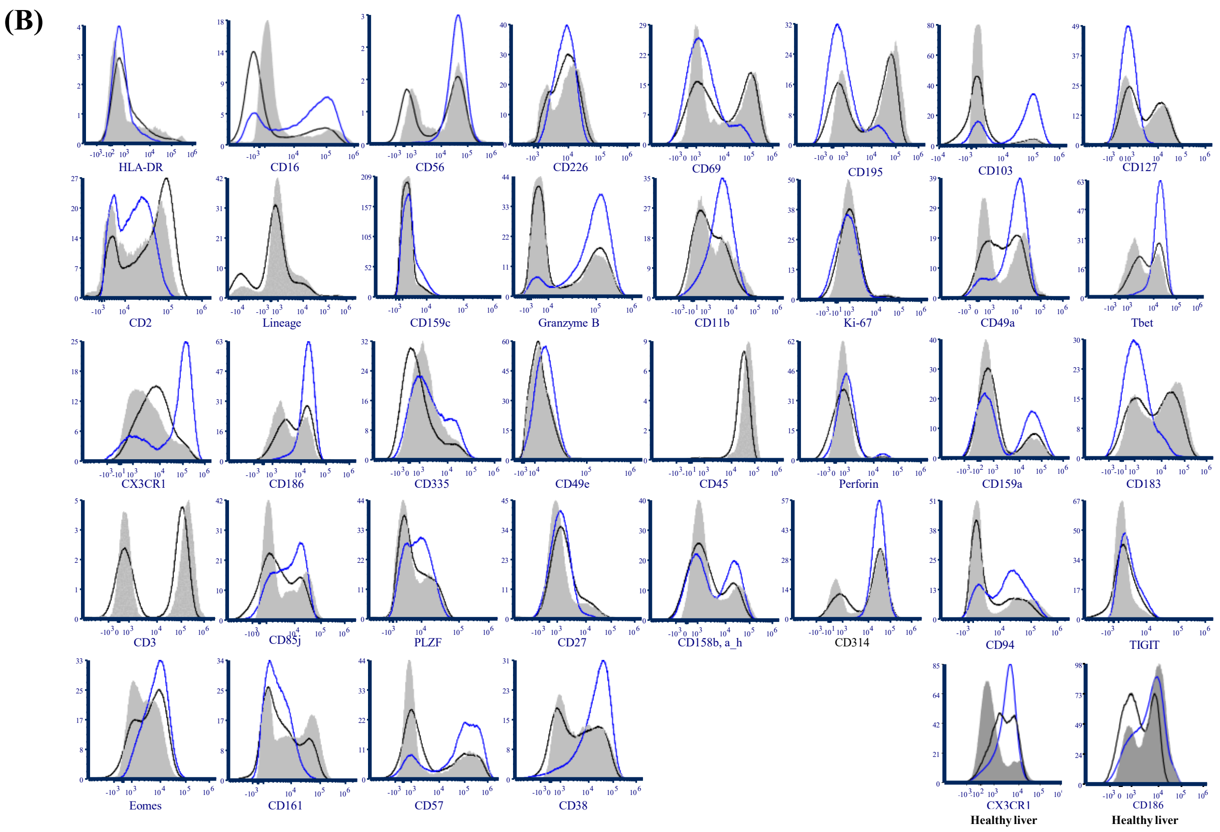

Supplemental Figure 9. Staining resolution of single stained vs MC sample.
Each marker was evaluated for optimal resolution in the MC staining. Staining patterns were compared between the RC and the MC sample from the same donor by means of histogram overlays. (**A**) Histogram overlays of RC (grey filled) and MC staining (bold black line) on PBMCs gated on either singlets/monocytes scatter or lymphocyte scatter (when applicable) or on total NK cells (bold blue line). (**B**) Histogram overlays of RC (grey filled) and MC staining (bold black line) on cells isolated from HCC liver and gated on either singlets/monocytes scatter or lymphocyte scatter (when applicable) or on total NK cells (bold blue line) or on total NK cells (bold blue line). For CX3CR1 and CD186 two examples are depicted: on cells from liver with HCC tumor or on cells from a HD liver (lower right).

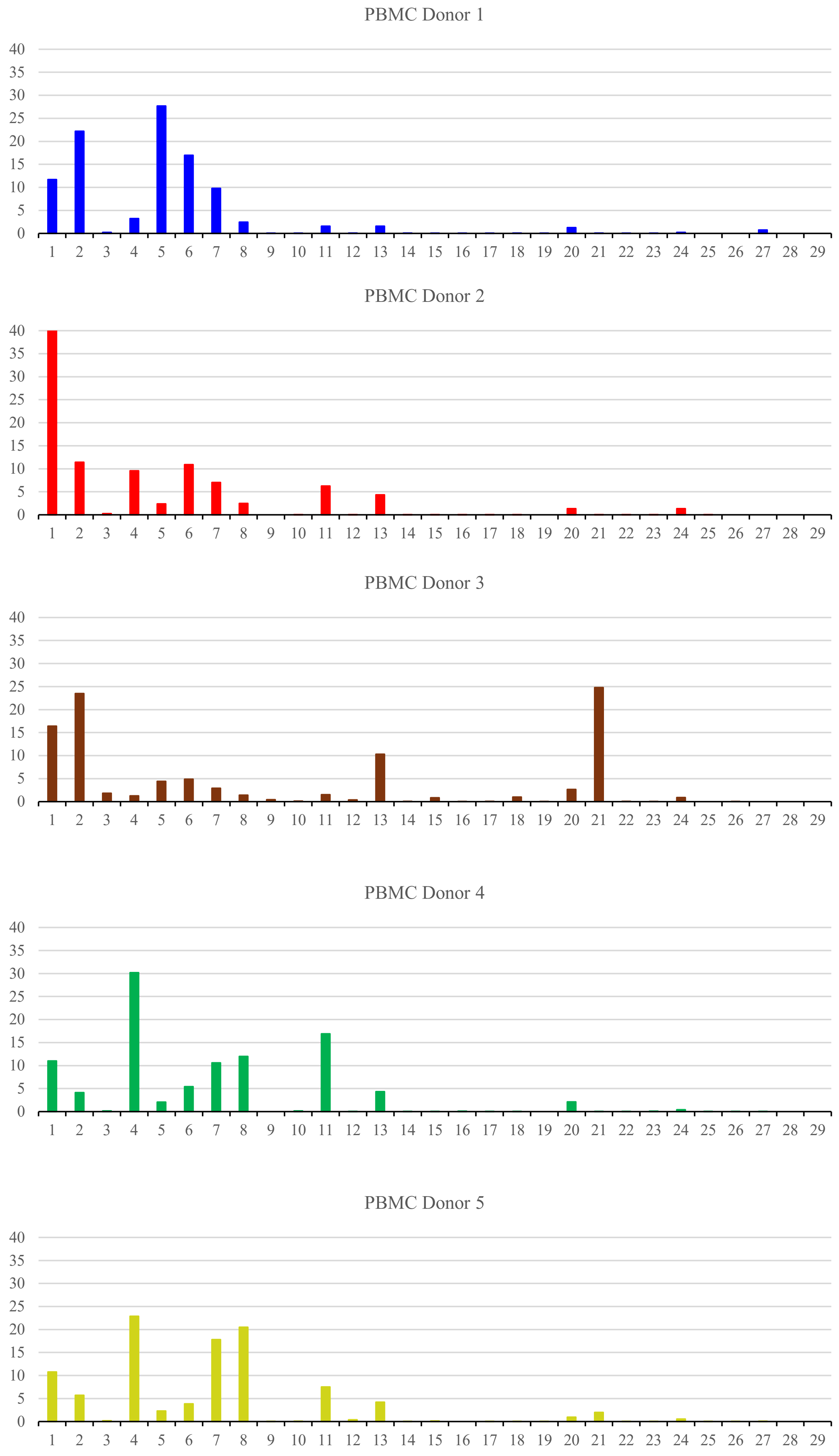
PBMC Donor 1
PBMC Donor 2
PBMC Donor 3
PBMC Donor 4
PBMC Donor 5
0 5 10 15 20 25 30 35 40
1 2 3 4 5 6 7 8 9 10 11 12 13 14 15 16 17 18 19 20 21 22 23 24 25 26 27 28 29

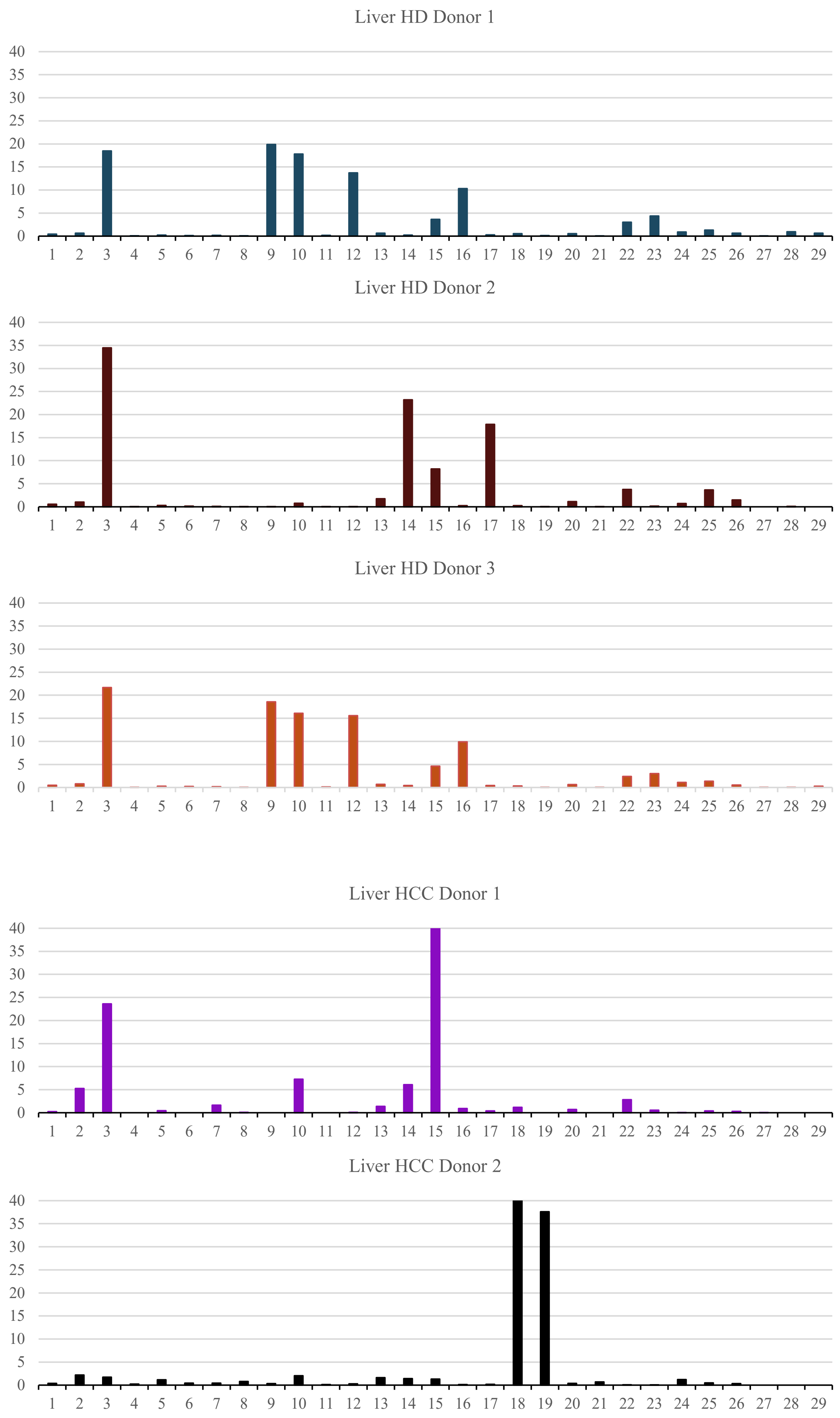

Supplemental Figure 10. Frequencies of Phenograph clusters identified.
Data were analyzed using the OMIQ platform with the analysis pipeline as described in the methods section. The frequencies of the Phenograph clusters identified are displayed as bargraphs per individual donor and sample type.

(A)

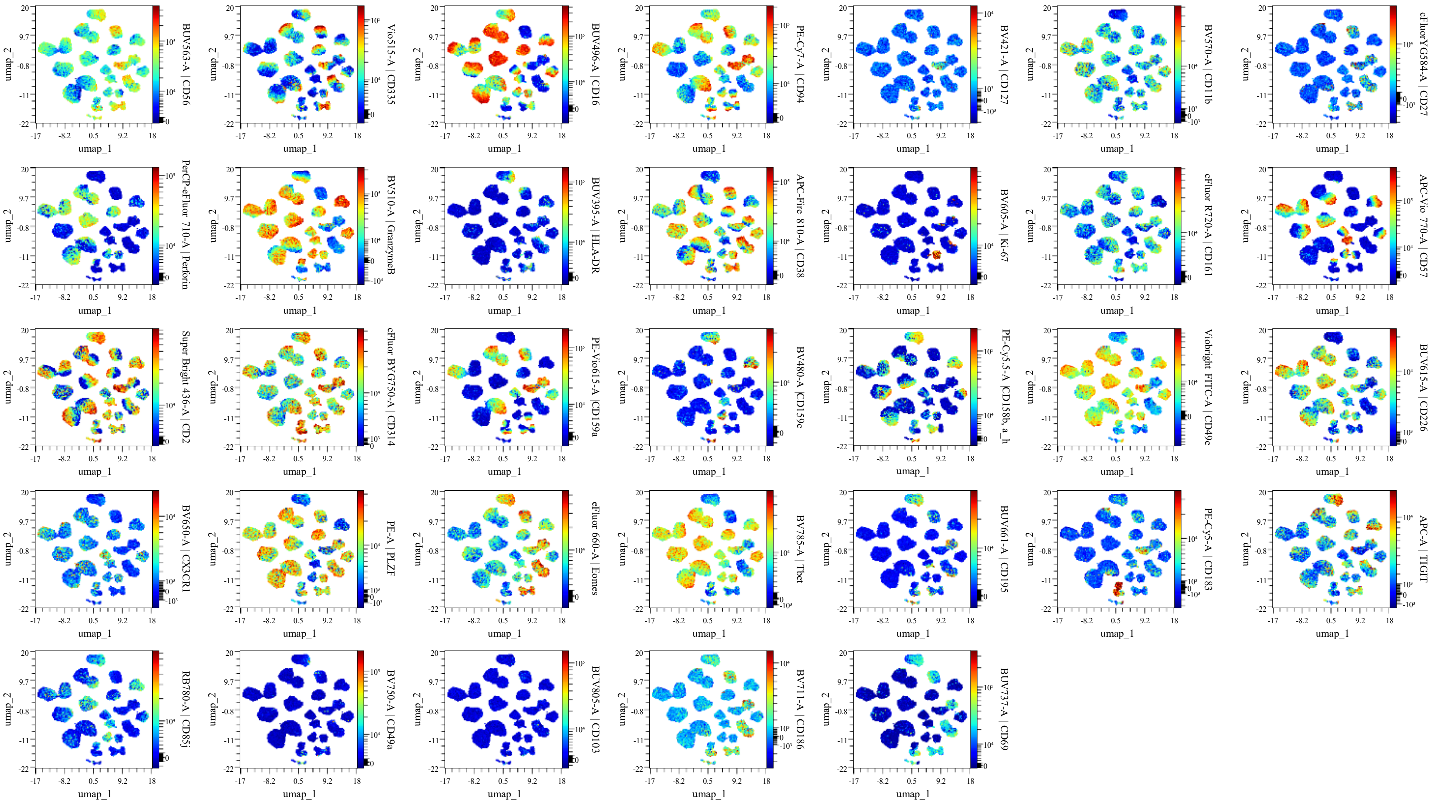
umap_1
umap_2
BUV563-A | CD56
Vio515-A | CD335
BUV496-A | CD16
PE-Cy7-A | CD94
BV421-A | CD127
BV570-A | CD11b
eFluorYG584-A | CD27
PerCP-eFluor 710-A | Perforin
BV510-A | GranzymeB
BUV395-A | HLA-DR
APC-Fire 810-A | CD38
BV605-A | Ki-67
cFluor R720-A | CD161
APC-Vio 770-A | CD57
Super Bright 436-A | CD2
cFluor BYG750-A | CD314
PE-Vio615-A | CD159a
BV480-A | CD159c
PE-Cy5.5-A | CD158b, a_h
Viobright FITC-A | CD49e
BUV615-A | CD226
BV650-A | CX3CR1
PE-A | PLZF
eFluor 660-A | Eomes
BV785-A | Tbet
BUV661-A | CD195
PE-Cy5-A | CD183
APC-A | TIGIT
RB780-A | CD85j
BV750-A | CD49a
BUV805-A | CD103
BV711-A | CD186
BUV737-A | CD69

(B)

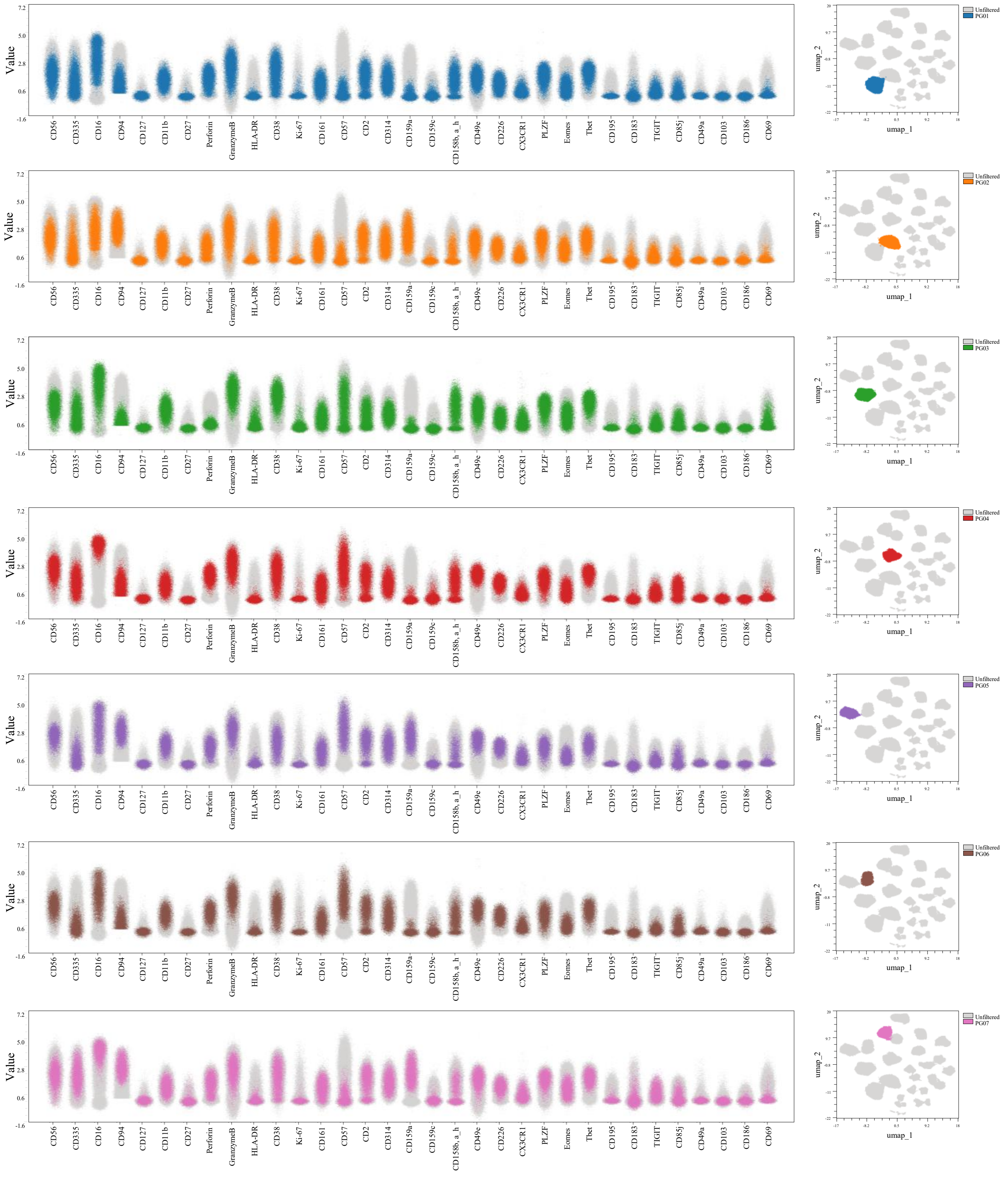
Value
CD56
CD335
CD16
CD94
CD127
CD11b
CD27
Perforin
GranzymeB
HLA-DR
CD38
Ki-67
CD161
CD57
CD2
CD314
CD159a
CD159c
CD158b, a_h
CD49e
CD226
CX3CR1
PLZF
Eomes
Tbet
CD195
CD183
TIGIT
CD85j
CD49a
CD103
CD186
CD69
umap_1
umap_2
Unfiltered
PG01
PG02
PG03
PG04
PG05
PG06
PG07

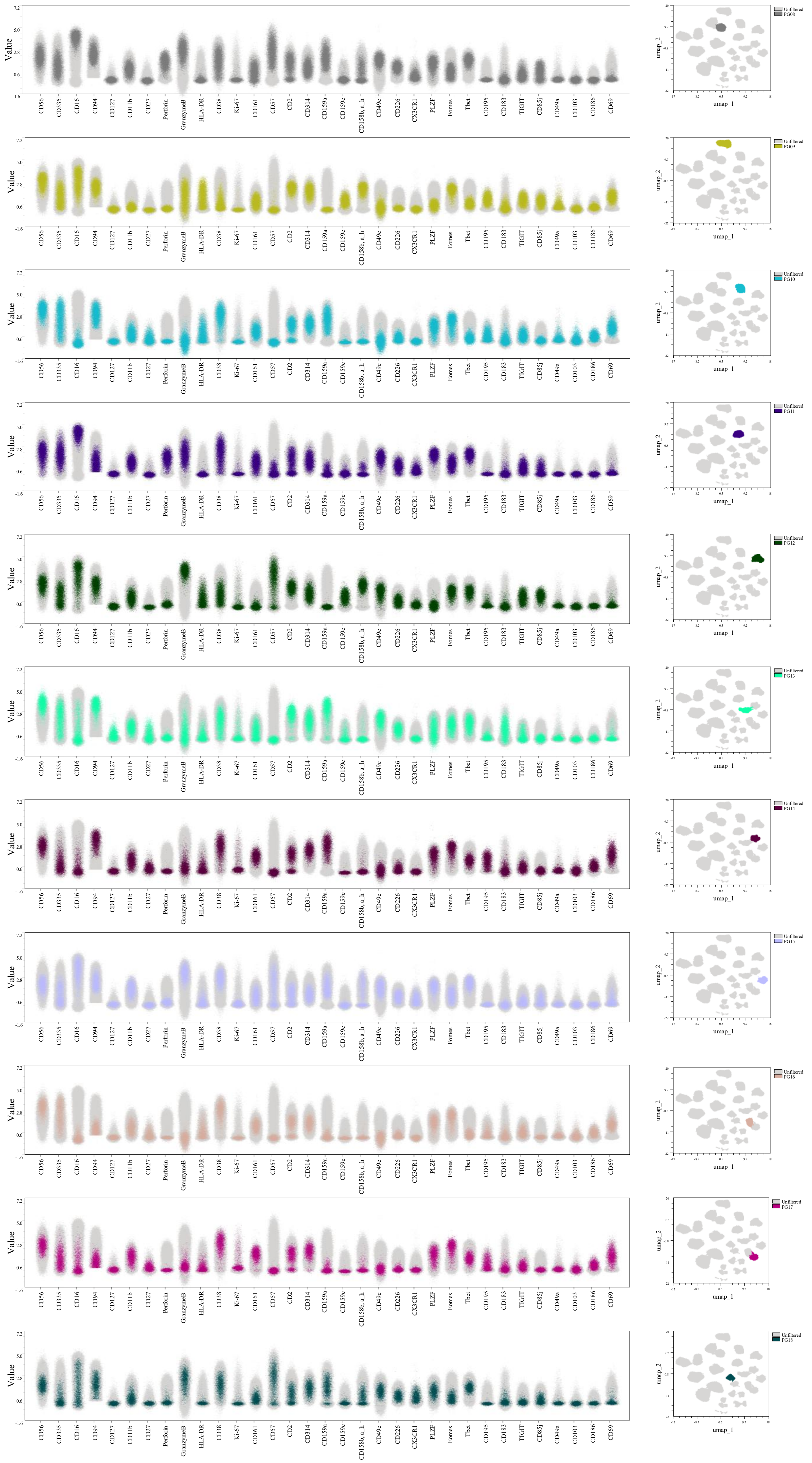
Value
7.2
5.0
2.8
0.6
-1.6
CD56
CD335
CD16
CD94
CD127
CD11b
CD27
Perforin
GranzymeB
HLA-DR
CD38
Ki-67
CD161
CD57
CD2
CD314
CD159a
CD159c
CD158b, a_h
CD49e
CD226
CX3CR1
PLZF
Eomes
Tbet
CD195
CD183
TIGIT
CD85j
CD49a
CD103
CD186
CD69
umap_2
umap_1
Unfiltered
PG08
PG09
PG10
PG11
PG12
PG13
PG14
PG15
PG16
PG17
PG18

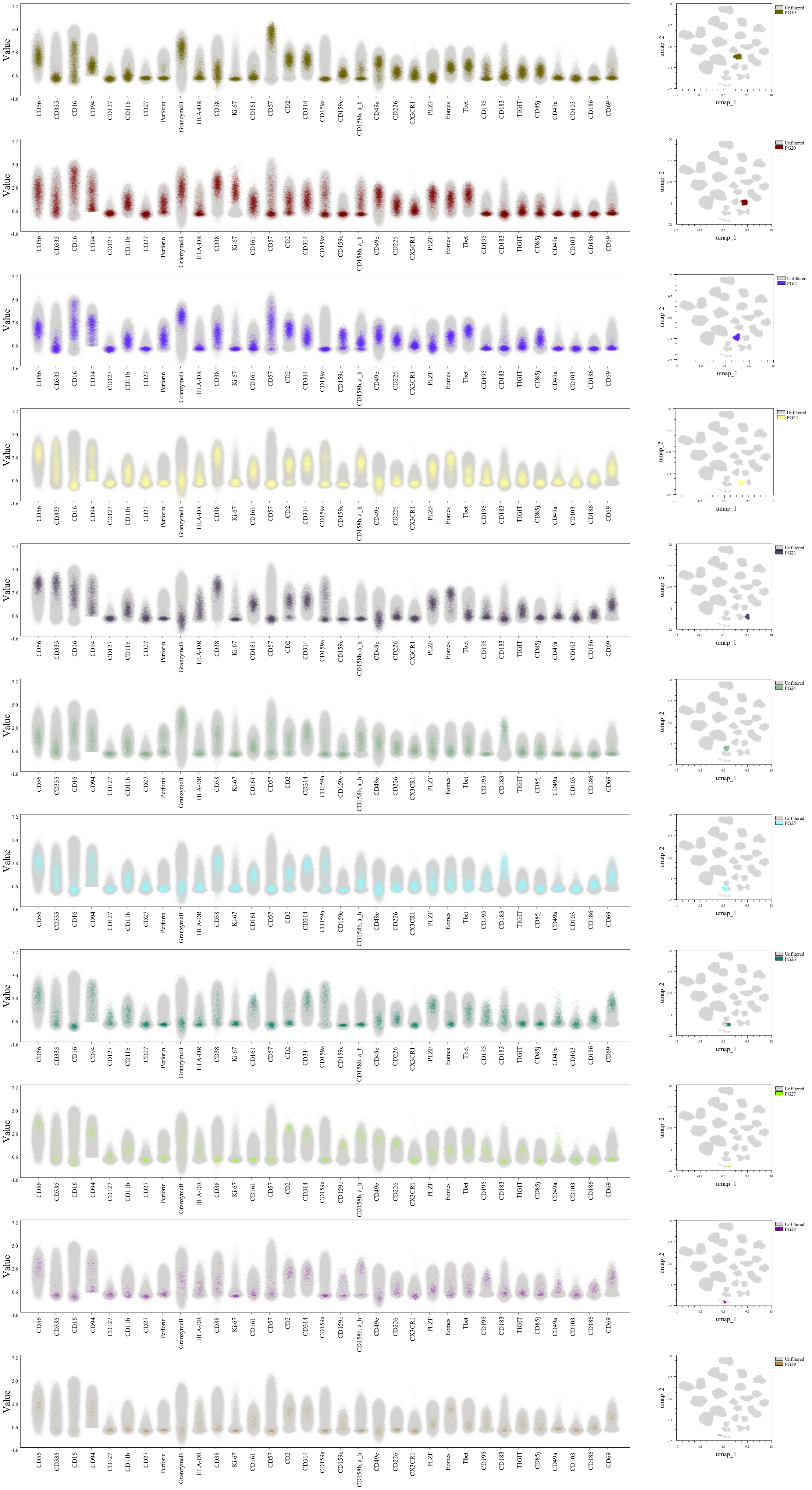
Value
CD56
CD335
CD16
CD94
CD127
CD11b
CD27
Perforin
GranzymeB
HLA-DR
CD38
Ki-67
CD161
CD57
CD2
CD314
CD159a
CD159c
CD158b, a_h
CD49e
CD226
CX3CR1
PLZF
Eomes
Tbet
CD195
CD183
TIGIT
CD85j
CD49a
CD103
CD186
CD69
umap_1
umap_2
Unfiltered
PG19
PG20
PG21
PG22
PG23
PG24
PG25
PG26
PG27
PG28
PG29

Supplemental Figure 11. Verification of all NK cells clusters

Identity verification of each Phenograph metacluster was done in several manners. By **(A)** UMAP color-continuous scatterplots that show the intensity of expression of each NK cell marker. Marker expression intensity is indicated by the scale bar to the right of each plot, where red is high, and blue is low. By **(B)** overlay scatter plots for each cluster which display the arcsinh transformation value for each critical marker used to define the metaclusters. For each cluster, UMAP (right) and overlay scatterplots (left) display 2 layers, in grey unfiltered events and each cluster in the corresponding color shown in the clustering map.

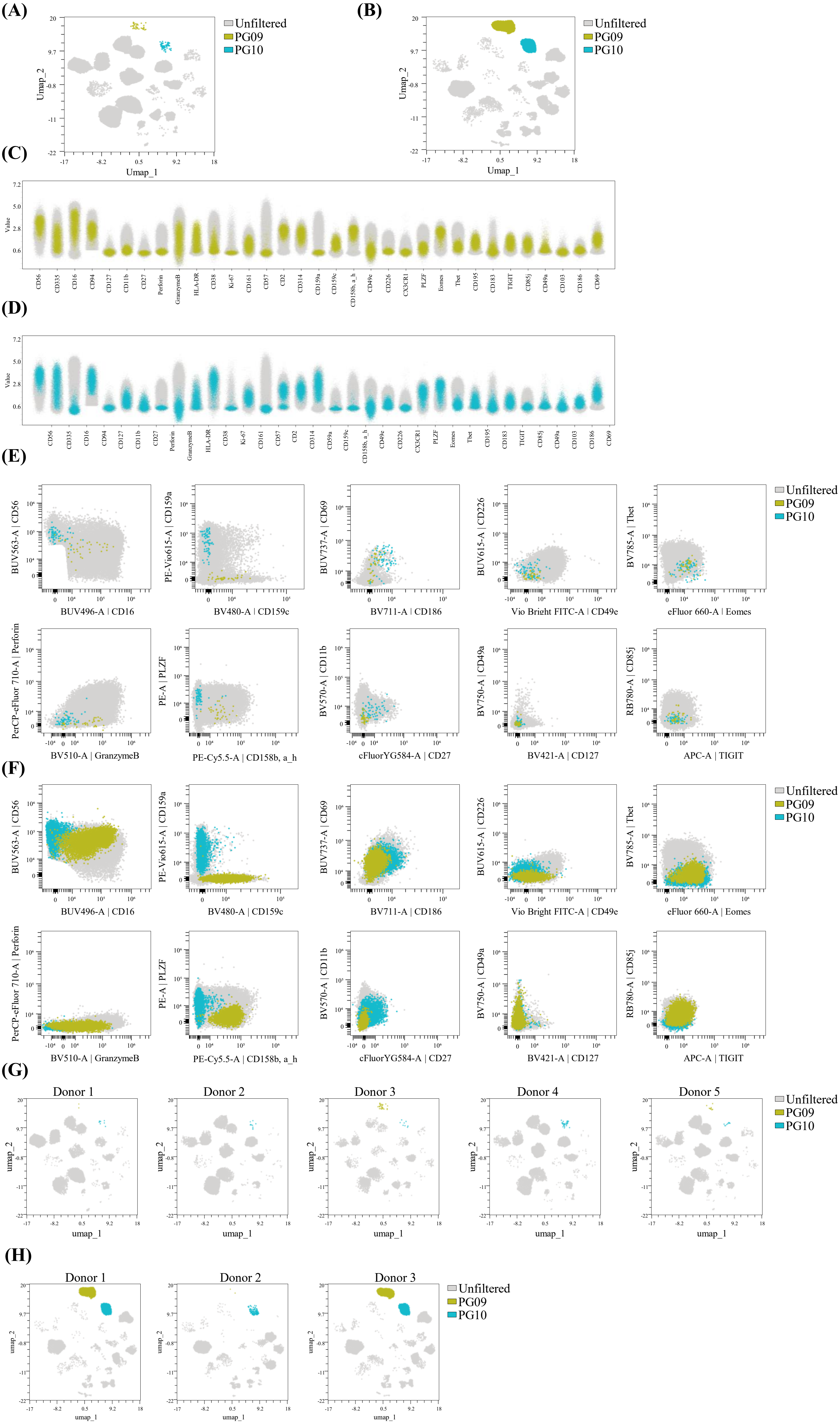
(A)
(B)
Unfiltered
PG09
PG10
Umap_2
Umap_1
(C)
Value
CD56 CD335 CD16 CD94 CD127 CD11b CD27 Perforin GranzymeB HLA-DR CD38 Ki-67 CD161 CD57 CD2 CD314 CD159a CD159c CD158b, a_h CD49e CD226 CX3CR1 PLZF Eomes Tbet CD195 CD183 TIGIT CD85j CD49a CD103 CD186 CD69
(D)
Value
(E)
BUV563-A | CD56
BUV496-A | CD16
PE-Vio615-A | CD159a
BV480-A | CD159c
BUV737-A | CD69
BV711-A | CD186
BUV615-A | CD226
Vio Bright FITC-A | CD49e
BV785-A | Tbet
eFluor 660-A | Eomes
PerCP-eFluor 710-A | Perforin
BV510-A | GranzymeB
PE-A | PLZF
PE-Cy5.5-A | CD158b, a_h
BV570-A | CD11b
cFluorYG584-A | CD27
BV750-A | CD49a
BV421-A | CD127
RB780-A | CD85j
APC-A | TIGIT
(F)
(G)
Donor 1
Donor 2
Donor 3
Donor 4
Donor 5
umap_2
umap_1
(H)

Supplemental Figure 12. Detailed verification of cluster 9 and cluster 10.
Verification of clusters 9 and 10 was performed by **(A)** superimposing the color-coded clusters (green; cluster 9, blue: cluster 10) on the concatenated UMAPs (grey; unfiltered) of PBMCs and (**B)** HD liver samples. Displaying the expression levels of all NK cell markers in (**C)** cluster 9 and (**D)** cluster 10 by superimposing the color-coded clusters on all concatenated files (grey; unfiltered). Generating biexponential plots of the color-coded clusters with markers unique and/or differentially expressed between the two clusters as indicated in the heatmap and superimposed on (**E)** concatenated PBMCs and **(F)** concatenated HD liver samples . Superimposing the color-coded clusters on the UMAP per individual donor in (**G)** PBMC and (**H)** HD liver samples to confirm tissue-specificity and donor-dependency of each cluster.
